# Persisting Reductions in Cannabis, Opioid, and Stimulant Misuse After Naturalistic Psychedelic Use: An Online Survey

**DOI:** 10.3389/fpsyt.2019.00955

**Published:** 2020-01-22

**Authors:** Albert Garcia-Romeu, Alan K. Davis, Earth Erowid, Fire Erowid, Roland R. Griffiths, Matthew W. Johnson

**Affiliations:** ^1^ Department of Psychiatry and Behavioral Sciences, Johns Hopkins University School of Medicine, Baltimore, MD, United States; ^2^ College of Social Work, The Ohio State University, Columbus, OH, United States; ^3^ Erowid Center, Grass Valley, CA, United States; ^4^ Department of Neuroscience, Johns Hopkins University School of Medicine, Baltimore, MD, United States

**Keywords:** psychedelics, hallucinogens, psilocybin, lysergic acid diethylamide (LSD), addiction, opioid, cannabis, stimulant

## Abstract

**Background:**

Observational data and preliminary studies suggest serotonin 2A agonist psychedelics may hold potential in treating a variety of substance use disorders (SUDs), including opioid use disorder (OUD).

**Aims:**

The study aim was to describe and analyze self-reported cases in which naturalistic psychedelic use was followed by cessation or reduction in other substance use.

**Methods:**

An anonymous online survey of individuals reporting cessation or reduction in cannabis, opioid, or stimulant use following psychedelic use in non-clinical settings.

**Results:**

Four hundred forty-four respondents, mostly in the USA (67%) completed the survey. Participants reported 4.5 years of problematic substance use on average before the psychedelic experience to which they attributed a reduction in drug consumption, with 79% meeting retrospective criteria for severe SUD. Most reported taking a moderate or high dose of LSD (43%) or psilocybin-containing mushrooms (29%), followed by significant reduction in drug consumption. Before the psychedelic experience 96% met SUD criteria, whereas only 27% met SUD criteria afterward. Participants rated their psychedelic experience as highly meaningful and insightful, with 28% endorsing psychedelic-associated changes in life priorities or values as facilitating reduced substance misuse. Greater psychedelic dose, insight, mystical-type effects, and personal meaning of experiences were associated with greater reduction in drug consumption.

**Conclusions:**

While these cross-sectional and self-report methods cannot determine whether psychedelics caused changes in drug use, results suggest the potential that psychedelics cause reductions in problematic substance use, and support additional clinical research on psychedelic-assisted treatment for SUD.

## Introduction

Substance misuse is a leading preventable cause of morbidity and mortality (1, 2), and contributed to over 63,000 drug overdose deaths in the US in 2016 (3). An estimated 23.3 million Americans have met *Diagnostic and Statistical Manual of Mental Disorders 5^th^ Ed*. (DSM-5; 4) criteria for a substance use disorder (SUD) regarding a drug besides alcohol or tobacco in their lifetime (5). Cannabis, opioids, and cocaine constitute the greatest proportion of these diagnoses (5). Recent trends have shown increased adult use of cannabis (6–8), opioids (9–11), and stimulant drugs (12, 13), and associated adverse public health outcomes (3).

Though cannabis use among those age 12–17 has largely decreased in recent years (6, 14), adults have shown greater use as more states have approved medical or recreational accessibility (8, 15). Concurrently, cannabis related emergency room visits (16) and prevalence of cannabis use disorder have risen (8). The United States has recently seen unprecedented levels of opioid misuse and overdose deaths, including a notable increase in prescription opioid misuse between 2001 and 2013 (17), and over 42,000 opioid-related deaths in 2016 (3). Additionally, recent increases in cocaine and other stimulant use (13, 18–20) have contributed to a substantial number of hospitalizations (21, 22) and deaths (3).

Available SUD treatments typically exhibit limited success with most patients not achieving long-term abstinence (23–26). Medications for opioid use disorder (OUD) include the agonist treatments methadone and buprenorphine, and the opioid antagonist naltrexone (27). However, many people who use opioids are unable or unwilling to access these treatments or do not adhere to them consistently enough to achieve long-term improvement (28–30). There are no approved pharmacotherapies for cannabis (31) and stimulant use disorders (32), and with the exception of contingency management (33, 34), behavioral therapies generally have modest efficacy for treating SUDs (35, 36). Thus, the current public health landscape highlights an urgent need for novel, innovative strategies for treating SUDs.

Use of serotonin 2A (5-HT2A) agonist psychedelics such as lysergic acid diethylamide (LSD), psilocybin-containing mushrooms (hereafter referred to as psilocybin), peyote, and the dimethyltryptamine (DMT) containing admixture ayahuasca in both naturalistic and clinical settings have been implicated in decreased substance misuse (37–48). The strongest evidence is for LSD in the treatment of alcoholism, with six randomized studies showing an aggregated statistically significant effect for LSD improving outcomes in meta-analysis (49).

An early study in 74 male parolees with a history of chronic heroin use examined a 4- to 6-week residential treatment program involving roughly 5 weeks of preparatory therapy in conjunction with a single high-dose administration of LSD (300–450 µg), compared with treatment as usual outpatient care involving weekly group therapy (46). The LSD treatment was well tolerated among this sample, which was largely African American (76%) and with relatively low education (mean of 8.6 years). Biologically verified continuous abstinence was significantly greater in the LSD than control conditions at 6 month (32% vs. 8%) and 12 month (25% vs 5%) follow-ups (46). Epidemiological data from the 2008–2013 National Survey on Drug Use and Health showed lifetime serotonin 2A agonist psychedelic use was associated with 27% reduced risk of past year opioid dependence and 40% reduced risk of past year opioid abuse when controlling for relevant covariates (43). Preliminary observational data have shown significant reductions in cocaine use in a small sample (*n =* 6) after participation in a ceremonial ayahuasca retreat geared toward addressing substance misuse (47). Pilot clinical research currently underway has also found promising early results of psilocybin-assisted treatment in people with cocaine use disorder (50, 51). In addition to these preliminary clinical findings, anecdotal reports further corroborate potential benefits of psychedelics in people with various substance use issues (e.g., 52).

We have previously published findings on individuals who self-reported reductions in tobacco (53), and alcohol misuse (40) attributed to naturalistic psychedelic use. However, instances in which people experienced a marked reduction in problematic cannabis, opioid, or stimulant use following ingestion of a psychedelic have not been systematically documented to date. Therefore, the current study sought to characterize instances in which individuals experienced a reduction in cannabis, opioid, or stimulant use after taking a psychedelic in a non-clinical setting. We hypothesized that greater improvements in substance misuse would be associated with greater mystical-type effects of the psychedelic experience consistent with preliminary clinical data (54, 55).

## Materials and Methods

This study was conducted as a cross-sectional, anonymous (i.e., no name or IP address recorded) online survey hosted on SurveyMonkey between October 2015 and August 2017. Study advertisements were posted on social media and on websites devoted to drug discussion, education, or research such as Erowid Center (erowid.org) and the Multidisciplinary Association for Psychedelic Studies (maps.org). Ads sought individuals who had “overcome alcohol^1^ or drug addiction after using psychedelics,” and took interested individuals to a page detailing introductory information regarding the study aims, participation requirements (e.g., filling out a survey), and study inclusion criteria. Inclusion criteria were: (1) at least 18 years of age, (2) able to speak, read, and write English fluently, (3) self-identified as having had problematic cannabis, opioid, or stimulant use, and (4) had used a serotonin 2A agonist psychedelic^2^ outside of a research or medical setting, followed by reduction or cessation of subsequent cannabis, opioid, or stimulant use. This study used purposive sampling (56) to specifically seek out people who had experienced improvements in substance use after psychedelic use for two reasons. First, to better characterize these individuals and their experiences, and second, as a preliminary step towards designing and studying psychedelic-assisted interventions for SUDs in clinical settings. People who indicated that they met inclusion criteria, understood the study procedures, and were willing to voluntarily participate were able to begin the survey. Individuals who read the introductory information and then chose to complete the survey were considered to have provided informed consent. Participants were not financially compensated for completing the survey. The study was approved by an Institutional Review Board of the Johns Hopkins University School of Medicine.

### Measures

Information on participant demographics and drug use history were collected. Participants' drug use was assessed retrospectively in the periods before and after the psychedelic experience to which they attributed their reduction or cessation in drug use (hereafter referred to as “reference psychedelic experience”). This included ratings of distress related to drug use prior to the reference psychedelic experience, overall duration of drug misuse, use of medication or other SUD treatments before and after the reference psychedelic experience, age of first drug use, and lifetime presence of other mental health diagnoses.^3^

Participants provided data on the reference psychedelic experience, including the psychedelic used and approximate dose, type of setting where the experience occurred, intention for self-administering the psychedelic, and any adverse effects or other behavioral changes attributed to the reference psychedelic experience. Participants were asked about possible mechanisms of change attributed to their psychedelic-associated reductions in drug use. Participants also provided ratings of withdrawal symptom severity after the reference psychedelic experience relative to prior attempts to reduce or stop drug use. Further information on the reference psychedelic experience and related changes in drug use patterns was gathered using assessments described below. Participants were asked to identify a specific drug or class of drugs among cannabis, opioids, and stimulants, that was the primary substance of abuse that they reduced or stopped after psychedelic use, and about which they answered specifically targeted questions.

Because this survey was conducted concurrently for people reporting psychedelic-associated reductions in alcohol (40), cannabis, opioid, and stimulant use, some of the measures used here were originally designed and validated to probe alcohol use, and were adapted for this survey to assess other drug use and craving. This was done so that scores on given assessments could be meaningfully compared across drug classes, rather than compared across a number of disparate measures of consumption and/or craving. Participants completed two iterations of a modified version of the Drug Use Disorders Identification Test-Consumption (DUDIT-C), the DSM-5 Substance Use Disorder Symptom Checklist, and a modified version of the Alcohol Urge Questionnaire (AUQ), each asking specifically about the primary drug/class of interest (i.e., cannabis, opioids, or stimulants). In the first iteration, participants were asked about their drug use in the year prior to their reference psychedelic experience. In the second, they responded regarding their drug use in the time since the reference psychedelic experience.

#### DUDIT-C

The DUDIT is an 11-item assessment designed to screen for problematic drug use (57), which largely parallels the 10-item Alcohol Use Disorder Identification Test (AUDIT) developed by the World Health Organization to assess alcohol misuse (58). The first three items of the AUDIT probe frequency of drinking, quantity of alcohol use, and frequency of heavy use, and are often used to provide an abbreviated measure of alcohol consumption called the AUDIT-Consumption or AUDIT-C (59, 60). For this survey we administered a modified version of the DUDIT asking specifically about frequency of drug use, quantity used, and frequency of heavy use regarding the specific drug of choice identified by the participant (i.e., cannabis, opioids, or stimulants) to provide an overall score of drug consumption we identify here as DUDIT-C.

#### DSM-5 Substance Use Disorder Symptom Checklist

This checklist was modified to assess DSM-5 symptoms for past and current cannabis, opioid, and stimulant use disorder (4, 61). Participants endorsed whether each of the 11 diagnostic criteria for SUD were true or false based on their drug use in the year before their reference psychedelic experience, and in the time since the reference psychedelic experience. According to DSM-5 criteria, presence of 2–3 symptoms indicates a mild, four to five symptoms indicate a moderate, and six or more symptoms indicate a severe SUD (4).

#### Drug Urge Questionnaire (DUQ)

This instrument is a modified version of the eight-item Alcohol Urge Questionnaire (AUQ; 62). The AUQ is a validated alcohol craving measure that assesses three domains: (1) desire to drink; (2) expectation of positive effects from drinking; and (3) inability to resist drinking when alcohol is accessible, with scores ranging from 8 to 56, and higher scores indicating greater craving. For this study, items were modified to ask about craving for the specific drug of choice (i.e., cannabis, opioids, or stimulants), rather than alcohol.

#### Mystical Experience Questionnaire (MEQ30)

The MEQ30 is a validated 30-item questionnaire designed to assess mystical-type subjective effects of psychedelics (63–66). There are four major dimensions of the MEQ30: (1) mystical, including feelings of unity, sacredness, and noetic quality (i.e., direct knowledge or insight); (2) positive mood (e.g., awe, joy); (3) transcendence of time and space; and (4) ineffability. Participants completed the MEQ30 regarding their reference psychedelic experience. A “complete” mystical experience was defined by ≥60% of the maximum possible score on each of the four subscales of the MEQ30 (63).

#### Ratings of Persisting Effects

The personal meaning, psychological challenge, psychological insight, spiritual significance, and change in well-being or life satisfaction attributed to the reference psychedelic experience were rated by respondents (40, 67, 68). Participants rated personal meaning, psychological challenge, and psychological insight on a scale from 1 to 8 (1 = no more than routine, everyday experiences; 7 = among the five most meaningful/challenging/insightful experiences of my life; and 8 = the single most meaningful/challenging/insightful experience of my life). Spiritual significance was rated on a scale from 1 to 6 (1 = not at all; 5 = among the five most spiritually significant experiences of my life; 6 = the single most spiritually significant experience of my life). Change in well-being or life satisfaction was rated on a scale from −3 (decreased very much) to 0 (no change) to +3 (increased very much).

### Data Analyses

First, descriptive statistics of background and demographic characteristics, history of psychedelic use and characteristics of psychedelic session, substance use and history of treatment, substance withdrawal symptoms, and psychiatric history were calculated. Next, all study variables were subjected to chi-square and one-way analysis of variance tests (with between-subject factor for type of substance) to examine whether there were any differences in study variables as a function of the type of substance (cannabis, opioids, stimulants) affected by the psychedelic experience.

DUDIT-C change scores (post-score minus pre-score) were examined to assess how much each participant's overall substance use had changed from pre- to post-psychedelic experience. Pearson correlation coefficients were calculated to examine the degree to which DUDIT-C change scores were associated with primary study variables (substance, age, country of residence, mean age at time of psychedelic experience, dose of psychedelic, mystical experiences, insight experiences, personal meaning of psychedelic experience, pre-DUDIT-C, substance distress prior to experience, substance craving prior to experience, post-DUDIT-C, age of first substance use). These analyses were conducted using SPSS software v.24 (69).

**Figure 1:**
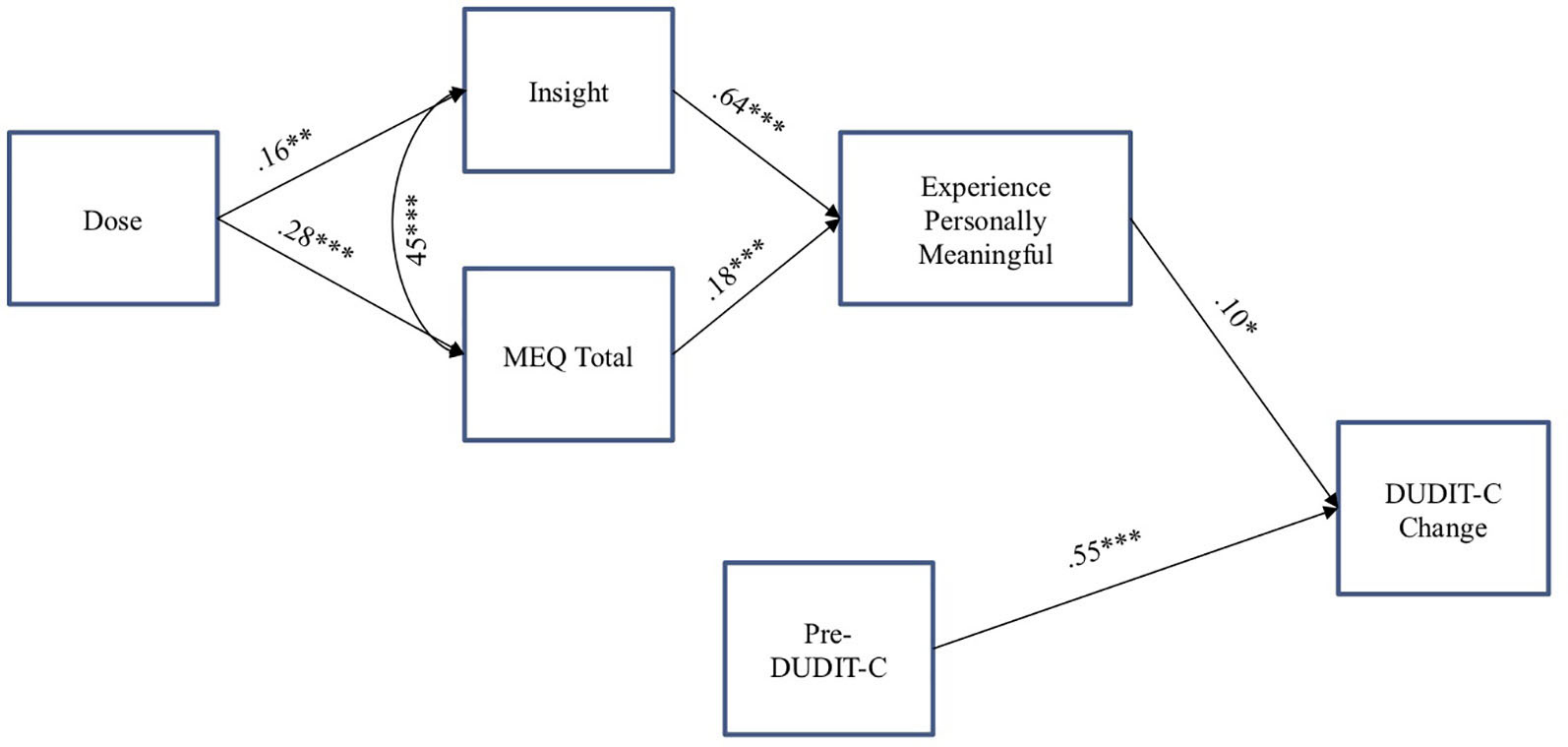
Path analysis examining predictors of substance consumption change score from pre- to post-psychedelic experience among individuals meeting criteria for risky substance use while controlling for the positive association between acute insight and mystical experiences. *p < .05; **p < .01; ***p < .001. DUDIT-C = Drug Use Disorders Identification Test—Consumption. ME, Mystical Experience Questionnaire.

Finally, a path analysis was conducted to examine a model of substance use change associated with a psychedelic experience. The model included (1) Pre-DUDIT-C as a predictor of DUDIT-C change score, (2) dose of the psychedelic as a predictor of acute mystical and insight experiences during psychedelic session, (3) insight and mystical experiences as predictors of ratings of personal meaning associated with the psychedelic session, and (4) personal meaning as a predictor of DUDIT-C change score (see **Figure 1**). We also controlled for the intercorrelation of age with DUDIT-C change score and the intercorrelation of acute mystical and insightful experiences. We conducted this path analysis using maximum likelihood with robust standard errors in MPlus software v.7.0 (70).

## Results

### Respondent Characteristics

During data collection (October, 2015 through August, 2017), 3,987 people clicked a recruitment advertisement and started filling out the survey. Of these, 2,556 met all inclusion criteria, provided informed consent, and initiated a response regarding cannabis, opioids, or stimulants. Among these, a total of 630 individuals completed the full survey regarding their use of one of these three classes of substances. Of those that completed the entire survey, 186 respondents were excluded because their reference psychedelic experience occurred within 3 months of filling out the survey, thus limiting the ability to assess lasting change in substance use on the modified DUDIT-C (71). The final sample was comprised of 444 adults. Demographics are presented in **Table 1**. The majority were white (82.4%), male (79.1%), and from the U.S. (66.9%), with a mean age of 28.4 (*SD* = 10.6). Of these, 166 reported they experienced a change in their cannabis use, 155 reported a change in opioid use, and 123 reported a change in stimulant use, following a psychedelic experience. It took participants a median duration of 1 h to complete the survey (inter-quartile range: 0 h 43 min to 1 h 38 min).

**Table 1:** Demographic characteristics, substance use, and mental health history in the sample (N = 444) and in each substance-specific subsample.

	Total sample (N = 444)	Cannabis (n = 166)	Opioids (n = 155)	Stimulants (n = 123)	Post-hoc
**Demographics**					
Age***	28.4 (10.6)	25.2 (7.8)	30.6 (11.8)	29.9 (11.0)	C < O = S
Female sex	93 (20.9%)	31 (18.7%)	35 (22.6%)	27 (22.0%)	
White	365 (82.4%)	136 (81.9%)	131 (84.5%)	98 (80.3%)	
Hispanic	38 (8.6%)	13 (7.8%)	14 (9.0%)	11 (8.9%)	
Single/not married	254 (57.2%)	105 (63.3%)	90 (58.1%)	59 (48.0%)	
United States resident***	297 (66.9%)	84 (50.6%)	123 (79.4%)	90 (73.2%)	C < O = S
**Education**					
Did not complete high school/GED	21 (4.7%)	11 (6.6%)	6 (3.9%)	4 (3.3%)	
High school/GED	75 (16.9%)	33 (19.9%)	18 (11.6%)	24 (19.5%)	
Some college	183 (41.2%)	61 (36.7%)	75 (48.4%)	47 (38.2%)	
College graduate	88 (19.8%)	30 (18.1%)	30 (19.4%)	28 (22.8%)	
Some grad school or graduate	77 (17.3%)	31 (18.7%)	26 (16.8%)	20 (16.3%)	
**Income**					
0–19.9K	137 (31.2%)	58 (35.6%)	43 (27.7%)	36 (29.8%)	
20–39.9K	106 (24.1%)	35 (21.5%)	44 (28.4%)	27 (22.3%)	
40–59.9K	64 (14.6%)	28 (17.2%)	23 (14.8%)	13 (10.7%)	
60–99.9K	67 (15.3%)	20 (12.3%)	24 (15.5%)	23 (19.0%)	
100K+	65 (14.8%)	22 (13.5%)	21 (13.5%)	22 (18.2%)	
**Substance use variables and SUD diagnosis**					
Substance distress***	2.6 (1.6)	1.8 (1.2)	2.9 (1.3)	3.2 (2.1)	C < O = S
Pre-DUDIT C	8.0 (2.5)	8.4 (2.1)	8.0 (2.6)	7.7 (2.7)	
Pre-DSM5					
No SUD	19 (4.3%)	9 (5.4%)	5 (3.2%)	5 (4.1%)	
Mild SUD	29 (6.5%)	11 (6.6%)	7 (4.5%)	11 (8.9%)	
Moderate SUD	47 (10.6%)	22 (13.3%)	12 (7.7%)	13 (10.6%)	
Severe SUD	349 (78.6%)	124 (74.7%)	131 (84.5%)	94 (76.4%)	
Pre-DUQ (Craving)***	40.7 (10.4)	36.6 (9.7)	45.1 (9.6)	40.7 (10.2)	C < S < O
Post-DUDIT C***	2.6 (2.8)	3.7 (2.8)	1.8 (2.5)	2.1 (2.5)	C > S = O
Post-DSM5					
No SUD	323 (72.7%)	109 (65.7%)	117 (75.5%)	97 (78.9%)	
Mild SUD	62 (14.0%)	25 (15.1%)	22 (14.2%)	15 (12.2%)	
Moderate SUD	24 (5.4%)	14 (8.4%)	3 (1.9%)	7 (5.7%)	
Severe SUD	35 (7.9%)	18 (10.8%)	13 (8.4%)	4 (3.3%)	
Post DUQ (Craving)	16.1 (8.9)	16.4 (9.0)	16.2 (9.6)	15.4 (7.8)	
Years of having a substance use problem	4.5 (5.3)	3.9 (4.8)	5.4 (5.8)	4.4 (5.2)	
Age of first use***	17.2 (4.4)	15.9 (2.3)	18.5 (5.4)	17.2 (4.7)	C < S < O
DUDIT-C Change Score***	-5.4 (3.2)	-4.7 (2.9)	-6.2 (3.4)	-5.6 (3.2)	C < O
**History of mental health conditions**					
Any mental health disorder	391 (88.1%)	141 (84.9%)	142 (91.6%)	108 (87.8%)	
Anxiety disorder	277 (62.4%)	102 (61.4%)	104 (67.1%)	71 (57.7%)	
Eating disorder	49 (11.0%)	21 (12.7%)	15 (9.7%)	13 (10.6%)	
Impulse control disorder	31 (7.0%)	7 (4.2%)	13 (8.4%)	11 (8.9%)	
Mood disorder	276 (62.2%)	104 (62.7%)	89 (57.4%)	83 (67.5%)	
Personality disorder	65 (14.6%)	28 (16.9%)	12 (7.7%)	25 (20.3%)	
Psychotic disorder	30 (6.8%)	8 (4.8%)	9 (5.8%)	13 (10.6%)	
Substance use disorder***	266 (59.9%)	73 (44.0%)	122 (78.7%)	71 (57.7%)	C = S < O

All values shown are Mean (SD), except where % is noted to indicate n (%). GED, General Education Diploma; DUDIT-C, Drug Use Disorders Identification Test – Consumption; DSM5, Diagnostic and Statistical Manual of Mental Disorders; 5th Edition; SUD, Substance Use Disorder; DUQ, Drug Urge Questionnaire. ***p < .001.

### Substance Use, Mental Health, and Treatment History Prior to Psychedelic Experience

**Table 1** shows data regarding participant history of substance use and mental health. Prior to their reference psychedelic experience, 95.7% met criteria for a SUD (severe: 78.6%, moderate: 10.6%, mild: 6.5%). Of the total sample, a minority did not meet DSM-5 criteria for SUD (4.3%) but reported prior problematic use. Mean substance use score on the retrospective DUDIT-C was 8.0 (*SD* = 2.5), suggesting respondents had a history of heavy substance use, including notable substance use-related consequences before their reference psychedelic experience (recommended AUDIT-C cutoffs for problematic use are ≥4 for men and ≥3 for women; 59). Respondents had been experiencing substance use problems for mean of 4.5 (*SD* = 5.3) years, had been using their primary substance since the mean age of 17 (*SD* = 4.4), and the mean reported distress associated with their substance use was between “a moderate amount” and “a lot” (*M* = 2.6/4, *SD* = 1.6). Mode responses regarding lifetime psychedelic use ranged from “never used” for peyote (85%), San Pedro (82%), mescaline (80%), ayahuasca (79%), morning glory seeds (70%), and DMT (pure compound; 52%), to 2–5 lifetime psilocybin uses (22%), and 11–20 lifetime LSD uses (17%). Large proportions of the sample had been diagnosed with an anxiety disorder (62%), mood disorder (62%), or a SUD not otherwise specified (60%). **Table 2** shows SUD treatment history. The majority of participants (59%) had received no treatment for their substance use prior to the reference psychedelic experience, with some having sought treatment *via* counseling (26%), self-help (17%), or support group (16%).

**Table 2:** Substance Use Disorder (SUD) treatment history in the sample (N = 444) and in each substance specific subsample.

	Total sample (N = 444)	Cannabis (n = 166)	Opioids (n = 155)	Stimulants (n = 123)	Post-hoc
**SUD treatment history prior to psychedelic session**					
None***	262 (59.0%)	122 (73.5%)	63 (40.6%)	77 (62.6%)	C = S > O
Treatment center/detox***	59 (13.3%)	7 (4.2%)	41 (26.5%)	11 (8.9%)	C = S < O
Counseling***	115 (25.9%)	24 (14.5%)	64 (41.3%)	27 (22.0%)	C = S < O
Phone counseling	13 (2.9%)	1 (.6%)	7 (4.5%)	5 (4.1%)	
Website counseling	30 (6.8%)	6 (3.6%)	17 (11.0%)	7 (5.7%)	
Hypnosis	7 (1.6%)	0 (0.0%)	7 (4.5%)	0 (0.0%)	
Acupuncture	17 (3.8%)	2 (1.2%)	11 (7.1%)	4 (3.3%)	
Support group***	72 (16.2%)	9 (5.4%)	42 (27.1%)	21 (17.1%)	C < O = S
Self-help	74 (16.7%)	17 (10.2%)	35 (22.6%)	22 (17.9%)	
Spiritual practice	61 (13.7%)	17 (10.2%)	27 (17.4%)	17 (13.8%)	
Medications (for opioid group only)					
Methadone	–	–	24 (15.5%)	–	
Naltrexone	–	–	8 (5.2%)	–	
Buprenorphine	–	–	35 (22.6%)	–	
**SUD treatment history following psychedelic session**					
None***	281 (63.3%)	129 (77.7%)	73 (47.1%)	79 (64.2%)	C > S > O
Treatment center/detox	16 (3.6%)	2 (1.2%)	8 (5.2%)	6 (4.9%)	
Counseling***	55 (12.4%)	9 (5.4%)	34 (21.9%)	12 (9.8%)	C = S < O
Phone counseling	4 (.9%)	0 (0.0%)	3 (1.9%)	1 (.8%)	
Website counseling	8 (1.8%)	4 (2.4%)	3 (1.9%)	1 (.8%)	
Hypnosis	4 (.9%)	0 (0.0%)	2 (1.3%)	2 (1.6%)	
Acupuncture	6 (1.4)	0 (0.0%)	4 (2.6%)	2 (1.6%)	
Support group	30 (6.8%)	5 (3.0%)	20 (12.9%)	5 (4.1%)	
Self-help***	36 (8.1%)	6 (3.6%)	25 (16.1%)	5 (4.1%)	C = S < O
Medications (for opioid group only)					
Methadone	–	–	5 (3.2%)	–	
Naltrexone	–	–	6 (3.9%)	–	
Buprenorphine	–	–	11 (7.1%)	–	
Spiritual practice	71 (16.0%)	20 (12.0%)	36 (23.2%)	15 (12.2%)	

SUD, Substance Use Disorder. ***p < .001.

### Reference Psychedelic Experience

**Table 3** shows data regarding the reference psychedelic experience. Approximately three quarters of the sample reported using either LSD (43%) or psilocybin (29%) in the psychedelic experience that contributed to a change in substance misuse. Respondents reported using a moderate (47%), high (33%), or very high (12%) dose and most reported that at least 1 year had passed since their experience (70%), with 20% reporting 6 or more years since their experience. As **Table 3** shows, most respondents had their reference psychedelic experience in their home (59%), with the intention for psychological (61%) or spiritual (41%) exploration. Notably, only 14% reported that they intended to reduce/quit their problematic substance use through using the psychedelic substance. Although most participants did not report an explicit intention to change their substance use, 28% of respondents attributed a change in their life priorities or values to their reference psychedelic experience, which was the most commonly reported mechanism for how the psychedelic experience helped change their substance use.

**Table 3:** Psychedelic experience locations, intentions, variables, beliefs, and behavioral changes in the sample (N = 444) and in each substance specific subsample.

	Total sample (N = 444)	Cannabis (n = 166)	Opioids (n = 155)	Stimulants (n = 123)	Post-hoc
**Location of psychedelic experience**					
Home	260 (58.6%)	98 (59.0%)	93 (60.0%)	69 (56.1%)	
Party	37 (8.3%)	20 (12.0%)	8 (5.2%)	9 (7.3%)	
Public place	30 (6.8%)	14 (8.4%)	8 (5.2%)	8 (6.5%)	
Concert	34 (7.7%)	9 (5.4%)	12 (7.7%)	13 (10.6%)	
Nature	162 (36.5%)	69 (41.6%)	48 (31.0%)	45 (36.6%)	
Religious	45 (10.1%)	19 (11.4%)	14 (9.0%)	12 (9.8%)	
Other	34 (7.7%)	8 (4.8%)	16 (10.4%)	10 (8.1%)	
**Intention for psychedelic experience**					
No serious intention, other people were using	14 (3.2%)	7 (4.2%)	5 (3.2%)	2 (1.6%)	
Curiosity	73 (16.4%)	33 (19.9%)	24 (15.5%)	16 (13.0%)	
Recreation	231 (52.0%)	105(63.3%)	69 (44.5%)	57 (46.3%)	
Psychological self-exploration	269 (60.6%)	97 (58.4%)	94 (60.6%)	78 (63.4%)	
Explore spirituality or the sacred	180 (40.5%)	67 (40.4%)	65 (41.9%)	48 (39.0%)	
To reduce/quit using substance***	60 (13.5%)	7 (4.2%)	32 (20.6%)	21 (17.1%)	C < O = S
**Psychedelic experience variables**					
Psilocybin	129 (29.1%)	51 (30.7%)	43 (27.7%)	35 (28.5%)	
LSD	192 (43.2%)	82 (49.4%)	61 (39.4%)	49 (39.8%)	
Other (e.g., DMT, mescaline)	123 (27.7%)	33 (19.9%)	51 (32.9%)	39 (31.7%)	
Psychedelic dose					
Very low	3 (.7%)	0 (0.0%)	2 (1.3%)	1 (.8%)	
Low	33 (7.4%)	22 (13.3%)	4 (2.6%)	7 (5.7%)	
Moderate	208 (46.8%)	80 (48.2%)	67 (43.2%)	61 (49.6%)	
High	146 (32.9%)	52 (31.3%)	58 (37.4%)	36 (29.3%)	
Very high	54 (12.2%)	12 (7.2%)	24 (15.5%)	18 (14.6%)	
Mean age at time of experience***	23.7 (7.8)	21.9 (6.0)	25.0 (8.7)	24.7 (8.3)	C < O = S
Time since experience					
4–6 months	72 (16.2%)	32 (19.3%)	18 (11.6%)	22 (17.9%)	
7–12 months	63 (14.2%)	32 (19.3%)	17 (11.0%)	14 (11.4%)	
1–2 years	112 (25.2%)	52 (31.3%)	34 (21.9%)	26 (21.1%)	
3–5 years	108 (24.3%)	30 (18.1%)	47 (30.3%)	31 (25.2%)	
6–10 years	50 (11.3%)	12 (7.2%)	23 (14.8%)	15 (12.2%)	
More than 10 years	39 (8.8%)	8 (4.8%)	16 (10.3%)	15 (12.2%)	
MEQ total mean (SD)***	66.8 (20.7)	63.0 (21.4)	70.8 (20.6)	66.9 (19.1)	C < O = S
MEQ complete mystical experience	178 (40.1%)	58 (34.9%)	75 (48.4%)	45 (36.6%)	
PEQ—personally meaningful	5.2 (1.4)	5.1 (1.5)	5.4 (1.4)	5.1 (1.4)	
PEQ—spiritual significance	3.2 (1.4)	3.0 (1.4)	3.3 (1.4)	3.1 (1.4)	
PEQ—challenging	3.8 (2.3)	4.1 (2.2)	3.7 (2.4)	3.4 (2.2)	
PEQ—psychological insight	5.1 (1.7)	5.1 (1.5)	5.1 (1.9)	5.0 (1.6)	
PEQ—change in well-being / life satisfaction	2.5 (1.0)	2.3 (1.3)	2.7 (0.7)	2.5 (0.9)	C < O = S
**Proportion ranked each reason as most important for drug use reduction**					
Increased belief in ability to quit	88 (19.8%)	26 (15.7%)	39 (25.2%)	23 (18.7%)	
Reducing stress involved with quitting	35 (7.9%)	12 (7.2%)	15 (9.7%)	8 (6.5%)	
Reframing quitting as a spiritual task	58 (13.1%)	16 (9.6%)	27 (17.4%)	15 (12.2%)	
Changing life priorities or values	126 (28.4%)	51 (30.7%)	34 (21.9%)	41 (33.3%)	
Increased delayed gratification	83 (18.7%)	39 (23.5%)	25 (16.1%)	19 (15.4%)	
Increased ability to cope with craving	40 (9.0%)	14 (8.4%)	13 (8.4%)	13 (10.6%)	
**Other behavioral changes after psychedelic experience**					
None	23 (5.2%)	6 (3.6%)	9 (5.8%)	8 (6.5%)	
Reduced/quit other drugs	251 (56.5%)	90 (54.2%)	98 (63.2%)	63 (51.2%)	
Started using other drugs	41 (9.2%)	14 (8.4%)	16 (10.3%)	11 (8.9%)	
Improved diet	261 (58.8%)	95 (57.2%)	98 (63.2%)	68 (55.3%)	
Worsened diet	12 (2.7%)	4 (2.4%)	4 (2.6%)	4 (3.3%)	
Increased exercise	255 (57.4%)	89 (53.6%)	93 (60.0%)	73 (59.3%)	
Decreased exercise	11 (2.5%)	8 (4.8%)	2 (1.3%)	1 (0.8%)	
Improved relationships	343 (77.3%)	123 (74.1%)	129 (83.2%)	91 (74.0%)	
Worsened relationships	25 (5.6%)	12 (7.2%)	7 (4.5%)	6 (4.9%)	
Improved career	252 (56.8%)	92 (55.4%)	91 (58.7%)	69 (56.1%)	
Worsened career	23 (5.2%)	10 (6.0%)	9 (5.8%)	4 (3.3%)	

LSD, Lysergic acid diethylamide; DMT, N,N-Dimethyltryptamine; MEQ, Mystical Experience Questionnaire; PEQ, Persisting Effects Questionnaire. ***p < .001.

Participant MEQ30 scores were 67% of maximum total score on average, with about 40% of respondents meeting criteria for a “complete mystical experience.” Overall, 76% of respondents rated their reference psychedelic experience among the top 10 most personally meaningful of their lives; 45% rated it among the top 10 most psychologically challenging of their lives; and 71% rated it among the top 10 most psychologically insightful experiences of their lives. Approximately one-half of the sample (51%) rated the reference psychedelic experience among the top 5 most spiritually significant experiences of their lives, and 69% said their sense of well-being or life satisfaction had increased “very much” as a result of the experience and/or contemplation of it. Two individuals (0.5%) reported strong negative change to well-being or life satisfaction attributed to the reference psychedelic experience. One of these described developing “acute HPPD, hallucinogenic perception persistence disorder [*sic*]” after taking LSD and reported ongoing reduction in cannabis use afterwards. Details regarding the other person who reported strong negative change in well-being are included in the *Adverse Effects* section below.

### Adverse Effects

A majority of respondents (81%) reported no persisting adverse effects from their reference psychedelic experience; 9% reported possible adverse effects (i.e., they were unsure whether there were any adverse effects) and 10% reported definite adverse effects. Those reporting possible or definite adverse effects largely rated them as not severe or slightly severe (59% of the 19% who reported possible or definite adverse effects; e.g., transient paranoia, anxiety). Five individuals (1.1% of the total sample) reported adverse effects rated as extremely severe. Among these five individuals, two reported decreased well-being or life satisfaction related to the reference psychedelic experience (#3, moderately and #4, strongly). Four of the five extreme adverse reactions were in cannabis users, with the remaining (#1) occurring in a stimulant user.

The five extremely severe adverse effects were described as, (1) “The psychedelic experience had me convinced I am heterosexual when actually I am bisexual.” (2) “Night terrors, paranoia. hallucinations; both visual and auditory, feeling like I'm leaving my body, losing my sanity. Many more; these persisted for years.” (3) “Again, the bad trip gave the panic disorder and caused me massive generalized anxiety for half a decade to come. Only with abstinence from cannabis and hallucinogens, tons of medication and therapy for 6 years have I been able to come out on top from this condition of absolute existential dread triggered by the mushroom experience.” (4) “After this overdose, smoking weed gave me painful and disorienting brain zaps. These reduced in severity over approximately 2 weeks and changed into anxiety…. I'm not sure why I even kept smoking, it was a terrible experience but I think I was depressed from the overall after affects and still needed some sort of escape (weed had always been my favorite escape).” (5) “Had nightmares for 6 months and lived in constant fear of death, experienced tactile hallucinations and heard voices for months. Took a long time to process the shame that came through this experience. It's all been beautifully necessary, however.”

Among these individuals reporting extreme adverse reactions, one reported prior history of depression and obsessive-compulsive disorder, one reported history of anxiety, mood, personality, and oppositional defiant disorders, one reported a history of anxiety and attention deficit hyperactivity disorders, one reported a history of anxiety, mood, eating, and personality disorders, and one reported a history of anxiety, mood, personality, and psychotic disorders. Thus, all these individuals reported some mental health conditions that may have been related to or contributed to adverse effects. However, because the survey did not probe whether these issues developed before or after the reference psychedelic experience, no causal attributions can be inferred from the present data.

### Substance-Specific Differences in Demographics and Other Variables

As shown in **Tables 1**–**3**, few differences were found between cannabis, opioid, and stimulant using groups on demographic variables, substance use and treatment history, and psychedelic-related variables. When differences were found it was frequently the cannabis-using group that was different from the other substance use groups. For example, cannabis users were significantly younger and fewer of them were from the United States, compared to opioid and stimulant users. Additionally, cannabis users had lower mean ratings of substance-related distress, substance craving prior to the reference psychedelic experience, age of first primary substance use, and DUDIT-C change scores compared to opioid and stimulant users. When examining the proportion of respondents who received SUD treatment prior to and following the psychedelic experience, smaller proportions of cannabis and stimulant users had sought treatment including detoxification and counseling, compared to opioid users, but a larger proportion of them had engaged in self-help prior to the psychedelic experience. Furthermore, a larger proportion of opioid users sought treatment following the reference psychedelic experience, and more of them had been previously diagnosed with a substance use disorder, compared to cannabis or stimulant users. Cannabis users also had significantly lower MEQ30 total scores, and ratings of change in well-being or life satisfaction, than opioid users.

### Substance-Specific Withdrawal Symptoms

**Table 4** shows several withdrawal symptoms were endorsed by roughly two-thirds of the cannabis-using subsample, including depression (68%), craving (66%), and insomnia (66%). Despite experiencing these withdrawal symptoms, many of these respondents (range = 45%–75%) reported that these symptoms were “less severe” or “much less severe” after the reference psychedelic experience compared to prior quit attempts. Although less frequently reported, many respondents endorsed experiencing anxiety (60%), difficulty concentrating (60%), restlessness (57%), irritability (57%), and fatigue (52%). Most reported that the symptom severity was the same or less/much less severe compared to prior quit attempts. Of particular interest, craving appeared to be dampened in those who had previously experienced this withdrawal symptom, with 56% reporting that their cannabis craving was much less severe after the reference psychedelic experience compared to prior quit attempts.

**Table 4:** Withdrawal severity after psychedelic-associated cannabis cessation or reduction in comparison with previous quit attempts. (n = 166).

Withdrawal Symptom	n^a^	Symptom Severity				
		Much less severe	Less severe	Same	More severe	Much more severe
		n (%)	n (%)	n (%)	n (%)	n (%)
Lack of appetite	75	18 (24.0%)	13 (17.3%)	**31 (41.3%)**	12 (16.0%)	1 (1.3%)
Fatigue	87	24 (27.6%)	20 (23.0%)	**30 (34.5%)**	7 (8.0%)	6 (6.9%)
Headaches	70	19 (27.1%)	15 (21.4%)	**25 (35.7%)**	11 (15.7%)	0 (.0%)
Drowsiness	72	19 (26.4%)	16 (22.2%)	**24 (33.3%)**	11 (15.3%)	2 (2.8%)
Fever	24	4 (16.7%)	2 (8.3%)	**17 (70.8%)**	0 (.0%)	1 (4.2%)
Nausea	34	8 (23.5%)	6 (17.6%)	**17 (50.0%)**	3 (1.8%)	0 (.0%)
Tremors	41	11 (26.8%)	6 (14.6%)	**16 (39.0%)**	6 (14.6%)	2 (4.9%)
Increased heart rate	45	11 (24.4%)	10 (22.2%)	**16 (35.6%)**	4 (8.9%)	4 (8.9%)
Chills	35	9 (25.7%)	4 (11.4%)	**16 (45.7%)**	5 (14.3%)	1 (2.9%)
Seizures	18	3 (16.7%)	1 (5.6%)	**14 (77.8%)**	0 (.0%)	0 (.0%)
Hallucinations	30	4 (13.3.%)	3 (10.0%)	**13 (43.3%)**	7 (23.3%)	3 (10.0%)
Cravings	110	**62 (56.4%)**	20 (18.2%)	17 (15.5%)	6 (5.5%)	5 (4.5%)
Depression	113	**45 (39.8%)**	23 (20.4%)	20 (17.7%)	15 (13.3%)	10 (8.8%)
Confusion	70	**23 (32.9%)**	13 (18.6%)	17 (24.3%)	9 (12.9%)	8 (11.4%)
Heart pounding	49	**17 (34.7%)**	4 (8.2%)	16 (32.7%)	7 (14.3%)	5 (10.2%)
Difficulty concentrating	100	**32 (32.0%)**	25 (25.0%)	22 (22.0%)	10 (10.0%)	11 (11.0%)
Irritability	94	**30 (31.9%)**	27 (28.7%)	17 (18.1%)	15 (18.1%)	5 (5.3%)
Insomnia	110	**32 (29.1%)**	17 (15.5%)	26 (23.6%)	21 (19.1%)	14 (12.7%)
Restlessness	95	**27 (28.4%)**	22 (23.2%)	23 (24.2%)	17 (17.9%)	6 (6.3%)
Anxiety	100	**28 (28.0%)**	27 (27.0%)	20 (20.0%)	12 (12.0%)	13 (13.0%)

a Sample size varies by symptom (range = 18–113), as some participants had never experienced particular withdrawal symptoms. Percentages were calculated based on the number of individuals who reported a particular withdrawal symptom. Modal responses shown in bold type.

**Table 5** shows approximately three quarters of the opioid-using subsample reported the following withdrawal symptoms after the reference psychedelic experience: depression (77%), irritability (76%), craving (75%), fatigue (74%), muscle aches (72%), insomnia (72%), restlessness (72%), anxiety (71%), and difficulty concentrating (70%). Despite experiencing these withdrawal symptoms, large proportions (range = 49%–75%) rated these symptoms as “less severe” or “much less severe” after the reference psychedelic experience compared to prior quit attempts. Similar to cannabis-using respondents, craving seemed to be attenuated among opioid users who had previously experienced this withdrawal symptom, with 75% reporting that their opioid craving was less or much less severe after the reference psychedelic experience compared to prior quit attempts.

**Table 5:** Withdrawal severity after psychedelic-associated opioid cessation or reduction in comparison with previous quit attempts. (n = 155).

Withdrawal Symptom	n^a^	Symptom Severity				
		Much less severe	Less severe	Same	More severe	Much more severe
		n (%)	n (%)	n (%)	n (%)	n (%)
Lacrimation	92	26 (28.3%)	17 (18.5%)	**39 (42.4%)**	7 (7.6%)	3 (3.3%)
Rhinorrhea	93	24 (25.8%)	20 (21.5%)	**39 (41.9%)**	7 (7.5%)	3 (3.2%)
Fever	72	25 (34.7%)	11 (15.3%)	**28 (38.9%)**	6 (8.3%)	2 (2.8%)
Muscle aches	112	34 (30.4%)	21 (18.8%)	**43 (38.4%)**	7 (6.3%)	7 (6.3%)
Diarrhea	94	32 (34.0%)	17 (18.1%)	**36 (38.3%)**	4 (4.3%)	5 (5.3%)
Headaches	100	32 (32.0%)	19 (19.0%)	**37 (37.0%)**	6 (6.0%)	6 (6.0%)
Heart pounding	100	26 (26.0%)	21 (21.0%)	**37 (37.0%)**	8 (8.0%)	8 (8.0%)
Drowsiness	106	24 (22.6%)	24 (22.6%)	**39 (36.8%)**	10 (9.4%)	9 (8.5%)
Chills	107	35 (32.7%)	24 (22.4%)	**37 (34.6%)**	4 (3.7%)	7 (6.5%)
Insomnia	111	34 (30.6%)	21 (18.9%)	**37 (33.3%)**	7 (6.3%)	12 (10.8%)
Increased heart rate	100	30 (30.0%)	23 (23.0%)	**33 (33.0%)**	9 (9.0%)	5 (5.0%)
Restlessness	111	30 (27.0%)	**33 (29.7%)**	33 (29.7%)	6 (5.4%)	9 (8.1%)
Fatigue	115	32 (27.8%)	**33 (28.7%)**	33 (28.7%)	9 (7.8%)	8 (7.0%)
Cravings	116	**58 (50.0%)**	29 (25.0%)	15 (12.9%)	6 (5.2%)	8 (6.9%)
Irritability	118	**53 (44.9%)**	21 (17.8%)	28 (23.7%)	8 (6.8%)	8 (6.8%)
Depression	120	**53 (44.2%)**	31 (25.8%)	19 (15.8%)	9 (7.5%)	8 (6.7%)
Anxiety	110	**44 (40.0%)**	26 (23.6%)	24 (21.8%)	8 (7.3%)	8 (7.3%)
Seizures	33	**13 (39.4%)**	4 (12.1%)	12 (36.4%)	1 (3.0%)	3 (9.1%)
Nausea	98	**34 (34.7%)**	25 (25.5%)	29 (29.6%)	4 (4.1%)	6 (6.1%)
Tremors	90	**31 (34.4%)**	19 (21.1%)	27 (30.0%)	10 (11.1%)	3 (3.3%)
Lack of appetite	107	**34 (31.8%)**	25 (23.4%)	32 (29.9%)	10 (9.3%)	6 (5.6%)
Difficulty concentrating	109	**33 (30.3%)**	24 (22.0%)	33 (30.3%)	10 (9.2%)	9 (8.3%)

a Sample size varies by symptom (range = 33–120), as some participants had never experienced particular withdrawal symptoms. Percentages were calculated based on the number of individuals who reported a particular withdrawal symptom. Modal responses shown in bold type.

**Table 6** shows more than three quarters of the stimulant-using sample reported the following withdrawal symptoms after the reference psychedelic experience: depression (84%), irritability (79%), craving (77%), anxiety (77%), and difficulty concentrating (76%). Despite experiencing these withdrawal symptoms, large proportions (range = 53%–65%) reported that these symptoms were “less severe” or “much less severe” after the reference psychedelic experience compared to prior quit attempts. Similar to cannabis- and opioid-using respondents, craving seemed to be attenuated among stimulant users who had previously experienced this withdrawal symptom, with 65% reporting that their stimulant craving was less or much less severe compared to prior quit attempts.

**Table 6:** Withdrawal severity after psychedelic-associated stimulant cessation or reduction in comparison with previous quit attempts. (n = 123).

Withdrawal Symptom	n^a^	Symptom Severity				
		Much less severe	Less severe	Same	More severe	Much more severe
		n (%)	n (%)	n (%)	n (%)	n (%)
Fever	46	8 (17.4%)	9 (19.6%)	**26 (56.5%)**	1 (2.2%)	2 (4.3%)
Heart pounding	73	16 (21.9%)	21 (28.8%)	**29 (39.7%)**	4 (5.5%)	3 (4.1%)
Psychomotor retardation	75	20 (26.7%)	23 (30.7%)	**27 (36.0%)**	4 (5.3%)	1 (1.3%)
Increased appetite	87	15 (17.2%)	19 (21.8%)	**31 (35.6%)**	17 (19.5%)	5 (5.7%)
Drowsiness	87	17 (19.5%)	23 (26.4%)	**30 (34.5%)**	12 (13.8%)	5 (5.7%)
Unpleasant dreams	70	13 (18.6%)	22 (31.4.%)	**23 (32.9%)**	7 (10.0%)	5 (7.1%)
Increased heart rate	77	18 (23.4%)	24 (23.4%)	**25 (32.5%)**	8 (10.4%)	2 (2.6%)
Psychomotor agitation	69	19 (27.5%)	18 (26.1%)	**21 (30.4%)**	8 (11.6%)	3 (4.3%)
Difficulty concentrating	93	24 (25.8%)	25 (26.9%)	**28 (30.1%)**	10 (10.8%)	6 (6.5%)
Headaches	83	20 (24.1%)	22 (26.5%)	**24 (28.9%)**	11 (13.3%)	6 (7.2%)
Restlessness	89	19 (21.3%)	**31 (34.8%)**	20 (22.5%)	14 (15.7%)	5 (5.6%)
Confusion	68	16 (23.5%)	**23 (33.8%)**	22 (32.4%)	7 (10.3%)	0 (.0%)
Irritability	97	27 (27.8%)	**31 (32.0%)**	18 (18.6%)	15 (15.5%)	6 (6.2%)
Fatigue	88	20 (22.7%)	**26 (29.5%)**	26 (29.5%)	12 (13.6%)	4 (4.5%)
Insomnia	87	17 (19.5%)	**24 (27.6%)**	24 (27.6%)	13 (14.9%)	9 (10.3%)
Cravings	95	**40 (42.1%)**	22 (23.2%)	18 (18.9%)	8 (8.4%)	7 (7.4%)
Anxiety	95	**33 (34.7%)**	30 (31.6%)	16 (16.8%)	11 (11.6%)	5 (5.3%)
Depression	103	**35 (34.0%)**	29 (28.2%)	17 (16.5%)	13 (12.6%)	9 (8.7%)

a Sample size varies by symptom (range = 46–103), as some participants had never experienced particular withdrawal symptoms. Percentages were calculated based on the number of individuals who reported a particular withdrawal symptom. Modal responses shown in bold type.

### Substance Consumption Following the Psychedelic Experience

Over 70% of participants (n = 331) reported that they had greatly reduced or quit using their primary substance following their reference psychedelic experience as evidenced by an average DUDIT-C change score of −5.4 (*SD =* 3.2; range = 4 to −12). Though 95.7% met SUD criteria before the reference psychedelic experience, only 27.3% met criteria for a SUD in the time since their reference psychedelic experience. Small proportions continued to meet criteria for mild (14%), moderate (5%), and severe (8%) SUDs. Overall, the average post-DUDIT-C score (*M =* 2.6; *SD =* 2.8) suggested that most respondents were no longer using substances above the threshold for which he/she would be considered a risky substance user based on established cutoffs for the AUDIT-C (≥4 for males, ≥3 for females; 59). Additionally, most participants (63%) did not seek other treatment for substance use after their reference psychedelic experience, but smaller proportions noted they engaged in a spiritual practice (16%), had received counseling (12%), or attended a support group (7%).

### Path Analysis

**Table 7** shows Pearson correlations among variables. As shown in the table, greater decreases in consumption as quantified by DUDIT-C change scores were significantly associated with greater age, ratings of the experience as personally meaningful and insightful, pre-DUDIT-C scores, and intensity of substance use distress. Aside from significant correlations with DUDIT-C change scores, clusters of variables within the overall matrix that were significantly positively correlated included mystical and persisting effects of the reference psychedelic experience (e.g., greater MEQ30 scores associated with greater meaning and insight), and substance use variables (e.g., greater pre-DUQ craving associated with greater substance-related distress).

**Table 7:** Correlation among study variables.

		1	2	3	4	5	6	7	8	9	10	11	12	13
1	DUDIT-C change score		**0.19**	−0.07	**0.19**	**0.42**	**0.56**	−**0.67**	−0.01	**0.19**	0.12	**0.18**	**0.17**	0.09
2	Age			−0.06	0.00	0.03	0.11	−0.13	**0.36**	**0.74**	0.00	−0.07	0.00	0.03
3	Country				−0.03	−0.13	0.00	0.09	0.03	−0.02	−0.13	−0.02	−0.07	0.02
4	Substance distress					**0.30**	0.06	−**0.17**	0.15	0.05	0.09	0.15	0.07	0.05
5	Pre-DUQ Craving						**0.50**	−0.05	−0.04	0.04	**0.26**	**0.26**	**0.31**	0.16
6	Pre DUDIT-C							**0.24**	−0.10	0.14	0.09	0.12	0.14	0.11
7	Post DUDIT-C								−0.08	−0.10	−0.06	−0.10	−0.08	−0.01
8	Age of first use									**0.42**	−0.12	−0.06	−0.04	−0.03
9	Mean age at time of experience										0.04	−0.03	0.02	−0.01
10	MEQ Mean											**0.48**	**0.49**	**0.28**
11	Insight												**0.73**	**0.17**
12	Meaning													**0.18**
13	Dose													

Bolded values are significant correlations at p < .001 (conservative alpha). DUDIT-C, Drug Use Disorders Identification Test – Consumption; DUQ, Drug Urge Questionnaire; MEQ, Mystical Experience Questionnaire.

Based on previously published survey data among individuals reporting reductions in alcohol consumption after taking a serotonin 2A agonist psychedelic (40), and informed by the present correlation data on variables associated with change in DUDIT-C substance use scores, a path analysis was conducted examining a proposed model to explain the effect of psychedelic consumption on problematic substance use reduction (**Figure 1**). While controlling for the positive association between acute insight and mystical experiences, greater substance consumption prior to the reference psychedelic experience (pre-DUDIT-C) was directly related to greater change in substance use (DUDIT-C change score). Higher doses of the psychedelic substance were directly related to higher intensity of acute mystical and insight experiences during the psychedelic session, both of which were directly related to greater personal meaning of the experience. Moreover, higher ratings of personal meaning were directly related to greater DUDIT-C change score. Two indirect effects were also found between greater intensity of acute mystical effects [*β = .*02, SE = .01, *p* < .05, 95% CI (.00,.03)] and insight [*β = .*07, SE = .03, *p* < .05, 95% CI (.01,.11)] on higher DUDIT-C change score *via* higher ratings of personal meaning. Model fit was good, *X* ^2^ (7, N = 444) = 10.13, *p = .*181; root-mean-square error of approximation = .03 [CI (.00,.07)], standardized root-mean-square residual = .040, and Tucker-Lewis index = .99.

## Discussion

The current study provides data on 444 individuals who self-reported reductions in cannabis, opioid, and stimulant misuse after taking a psychedelic drug in a non-clinical setting. The majority of respondents retrospectively reported meeting DSM-5 criteria for severe SUD before their psychedelic experience, whereas in the time since that experience, the majority no longer met criteria for any SUD. Most of the respondents claimed lasting reductions in their substance use for over 1 year after using a psychedelic, consistent with persisting benefits observed in laboratory studies with psilocybin (54, 55, 72–74). Serious adverse effects, though relatively rare, were reported and included both ongoing perceptual disturbances described as hallucinogen persisting perception disorder (HPPD; 75), and persisting psychotic symptoms such as paranoia and hallucinations. These were more common among individuals reporting reductions in cannabis use after the reference psychedelic experience, possibly related to observed associations between cannabis use and psychosis (76). Despite adverse events being rare, these data highlight the potential risks of psychedelic use in naturalistic settings by individuals who have not received medical screening or preparation, as is common practice in clinical trials involving psychedelic administration (77). A minority of the present sample (range = 2.5–9.2%) reported negative impacts on overall life adjustment, including increased use of other drugs (**Table 3**), indicating some cases in which outcomes may have been mixed or otherwise undesirable. Such cases warrant further study to examine what factors may be associated with these challenges.

The findings of the present study are limited by the nature of the anonymous, retrospective self-report data collected, which cannot be verified, and are subject to participant self-selection and recall bias. The cross-sectional design does not allow for causal inferences to be derived from the findings, nor is this study able to provide any information regarding the overall prevalence of psychedelic-associated reductions in other substance use. The purposive sampling used in the current study specifically sought out people reporting positive outcomes regarding substance misuse after naturalistic psychedelic use to characterize these cases, therefore data were not explicitly collected on instances where psychedelic use led to no change or exacerbation of drug misuse. This method limits our ability to generalize these findings across all psychedelic users with other substance misuse issues (e.g., 78, 79), but provides valuable information for designing future psychedelic-assisted treatments for SUD. Additionally, because the survey sought to assess changes in drug use across several pharmacological classes, modified versions of alcohol assessments (AUDIT-C and AUQ) were used, which have not been validated for use in this manner. Due to these limitations, the current data should be interpreted with caution. However, taken in combination with preliminary clinical findings (46, 49, 54, 80) and previous anonymous survey studies (40, 55), these results further bolster the potential utility of serotonergic psychedelics as aids in the treatment of addiction.

Congruent with findings from prior surveys on individuals reporting reductions in tobacco (55) and alcohol consumption (40) after naturalistic psychedelic use, the current sample reported cravings for their primary problematic substance to be less or much less severe than previous attempts to reduce or stop using (**Tables 4**–**6**). While the veracity and underpinnings of such psychedelic-associated craving reductions remain uncertain, that these patterns of responses are stable across several unrelated drug classes is noteworthy and points to a potential mechanism by which psychedelics may help reduce subsequent substance misuse. Although lifetime psychedelic use was queried, we did not collect the information necessary to make any chronological inference regarding whether reference psychedelic experiences that were closer to initial psychedelic use were more or less likely to impact other substance misuse, a question that remains for future research.

Participants also reported less severity of anxiety and depression symptoms after the reference psychedelic experience compared with other attempts to reduce their substance use. A growing body of literature has shown persisting anxiolytic effects of psilocybin (81–83) and LSD (84), and antidepressant effects of psychedelics including psilocybin (85–87) and ayahuasca (88). Furthermore, data suggest ayahuasca's antidepressant effects are associated with post-acute modulation of cortisol (89) and brain-derived neurotrophic factor (BDNF; 90), shedding light on possible biological mechanisms of psychedelics' lasting mood effects. In turn, reductions in anxiety and depressed mood may also help individuals remain abstinent from drugs in the post-acute “after-glow” period by improving their outlook and ability to manage withdrawal (91, 92).

Additionally, participants endorsed changes in life priorities or values, increased belief in their ability to abstain, and increased ability to delay gratification, as among the most important reasons their psychedelic experience impacted other substance use. These data are in agreement with prior surveys of people reporting psychedelic-associated reductions in tobacco (55) and alcohol consumption (40), and are in accordance with hypotheses regarding psychedelic-related changes in values, self-efficacy, and decision-making as relevant psychological mechanisms for addiction treatment (93–96). As in prior surveys on psychedelic-associated reductions in alcohol consumption (40) participants reported high levels of personal meaning, psychological insight, and mystical-type effects, which were associated with higher psychedelic dose and greater reported change in drug consumption after the psychedelic experience. Thus, the psychological impact of these experiences and acute subjective drug effects seem to play an important role in facilitating subsequent change in substance misuse as observed in pilot studies of psilocybin-assisted interventions for tobacco (55, 97) and alcohol dependence (54).

Preclinical data are further elucidating our understanding of psychedelics' biological mechanisms, with recent findings showing serotonergic psychedelics can promote structural and functional neural plasticity (98), and have potent anti-inflammatory effects (99), which may be correlated with observed therapeutic benefits. Animal models suggest diverse anti-addictive properties of serotonergic psychedelics for alcohol (100, 101) as well as other drugs of abuse. Ayahuasca has been shown to reduce amphetamine self-administration in adolescent rats and normalize amphetamine related locomotor behavior (102). Vargas-Perez and colleagues found a single administration of the serotonin 2A agonist psychedelic 4-AcO-DMT (103) prevented development of opioid and nicotine dependence and blunted withdrawal response in rats and mice (104). Together, these data suggest serotonin 2A psychedelics may hold considerable potential as novel therapeutics in treating various SUDs.

Although medications for opioid use disorder exist, the present opioid overdose rates indicate the need for different treatment avenues (3, 29, 30). For cannabis (31) and stimulant (32) use disorders there are no approved medications at present and limited treatment options, underscoring the necessity for new treatments and approaches. Psychedelic-assisted interventions for addictions may offer an attractive alternative to current treatment models in that they may result in lasting change in substance misuse after only one or a few psychedelic administration sessions (e.g., 55). Importantly, serotonin 2A psychedelics are not themselves physiologically addictive (105), yet they seemingly enhance processes often targeted by accepted addiction treatments such as insight, self-efficacy, and spirituality, which may underlie these lasting effects (93, 94, 96). While challenges remain for the development of psychedelics as medications (106, 107), converging evidence reveals a compelling signal of efficacy. Given the current public health landscape and state of addiction treatment (1, 3), this potential demands rigorous clinical research efforts and federal funding. Although psychedelics might not be a “magic bullet” to solve the pervasive issues of substance misuse and addiction, they may well constitute a much-needed addition to our current armamentarium of medication-assisted treatment for SUDs.

## Data Availability Statement

The datasets for this article are not publicly available. Data may be made available on a case by case basis at the discretion of the Principal Investigator.

## Ethics Statement

The studies involving human participants were reviewed and approved by Johns Hopkins University School of Medicine Institutional Review Board. Written informed consent to participate in this study was not required as per local legislation and national guidelines.

## Author Contributions

AG-R made substantial contributions to the conception and design of the study, the acquisition and interpretation of the data, and the drafting of the manuscript. AD made substantial contributions to the conception and design of the study, the analysis and interpretation of the data, and the drafting of the manuscript. EE made substantial contributions to the design of the study, participant recruitment, and made critical revisions to the manuscript. FE made substantial contributions to the design of the study, participant recruitment, and made critical revisions to the manuscript. RG made substantial contributions to the conception and design of the study and made critical revisions to the manuscript. MJ made substantial contributions to the conception and design of the study, the acquisition and interpretation of the data, and made critical revisions to the manuscript. All authors approved the final version of this manuscript and agree to be accountable for all aspects of the work.

## Funding 

Funding for this research was provided by the Heffter Research Institute, and by Tim Ferriss, Matt Mullenweg, Craig Nerenberg, Blake Mycoskie, and the Steven and Alexandra Cohen Foundation. Support for AG-R and AD was provided by National Institute on Drug Abuse Grant T32DA07209. Support for RG was provided in part by NIDA Grant R01DA003889.

## Conflict of Interest

RG is on the board of directors of the Heffter Research Institute.

The remaining authors declare that the research was conducted in the absence of any commercial or financial relationships that could be construed as a potential conflict of interest.

## Footnotes

1 Data on alcohol were previously published elsewhere. See (40).

2 i.e., psilocybin [magic] mushrooms, LSD, morning glory seeds, mescaline, peyote or San Pedro cactus, DMT, or Ayahuasca.

3 A copy of the survey questionnaire is available online (**Supplementary Material**).

## References

[1] Degenhardt L Hall W Extent of illicit drug use and dependence, and their contribution to the global burden of disease. Lancet (2012) 379(9810):55–70. 10.1016/S0140-6736(11)61138-0 22225671

[2] Rudd RA Aleshire N Zibbell JE Gladden RM Increases in drug and opioid overdose deaths—United States, 2000–2014. Am J Transplant (2016) 16(4):1323–7. 10.1111/ajt.13776 26720857

[3] Seth P Scholl L Rudd RA Bacon S Overdose deaths involving opioids, cocaine, and psychostimulants—united states, 2015–2016. Morb Mortal Weekly Rep (2018) 67(12):349–58. 10.15585/mmwr.mm6712a1 PMC5877356 29596405

[4] American Psychiatric Association Diagnostic and Statistical Manual of Mental Disorders (DSM-5®). Washington, DC: American Psychiatric Pub. (2013).

[5] Grant BF Saha TD Ruan WJ Goldstein RB Chou SP Jung J Epidemiology of DSM-5 drug use disorder. JAMA Psychiatry (2016) 73(1):39–47. 10.1001/jamapsychiatry.2015.2132 26580136 PMC5062605

[6] Azofeifa A Mattson ME Schauer G McAfee T Grant A Lyerla R National estimates of marijuana use and related indicators—national survey on drug use and health, United States, 2002–2014. MMWR Surveillance Summaries (2016) 65(No. SS-11):1–25. 10.15585/mmwr.ss6511a1 27584586

[7] Grucza RA Agrawal A Krauss MJ Cavazos-Rehg PA Bierut LJ Recent trends in the prevalence of marijuana use and associated disorders in the United States. JAMA Psychiatry (2016) 73(3):300–1. 10.1001/jamapsychiatry.2015.3111 PMC5407184 26864618

[8] Hasin DS Saha TD Kerridge BT Goldstein RB Chou SP Zhang H Prevalence of marijuana use disorders in the United States between 2001-2002 and 2012-2013. JAMA Psychiatry (2015) 72(12):1235–42. 10.1001/jamapsychiatry.2015.1858 PMC5037576 26502112

[9] Han B Compton WM Blanco C Crane E Lee J Jones CM Prescription Opioid Use, Misuse, and Use Disorders in U.S. Adults: 2015 National Survey on Drug Use and Health. Ann Internal Med (2017b) 167(5):293. 10.7326/M17-0865 28761945

[10] Martins SS Sarvet A Santaella-Tenorio J Saha T Grant BF Hasin DS Changes in US lifetime heroin use and heroin use disorder: prevalence from the 2001-2002 to 2012-2013 National Epidemiologic Survey on Alcohol and Related Conditions. JAMA Psychiatry (2017) 74(5):445–55. 10.1001/jamapsychiatry.2017.0113 PMC5470460 28355458

[11] Schepis TS McCabe SE Trends in older adult nonmedical prescription drug use prevalence: Results from the 2002–2003 and 2012–2013 National Survey on Drug Use and Health. Addictive Behav (2016) 60:219–22. 10.1016/j.addbeh.2016.04.020 PMC4884514 27163188

[12] Han B Jones CM Blanco C Compton WM National trends in and correlates of nonmedical use of prescription stimulants, nonmedical use frequency, and Use Disorders. J Clin Psychiatry (2017a) 78(9):e1250–8. 10.4088/JCP.17m11760 29045771

[13] John WS Wu L-T Trends and correlates of cocaine use and cocaine use disorder in the United States from 2011 to 2015. Drug Alcohol Depend (2017) 180:376–84. 10.1016/j.drugalcdep.2017.08.031 PMC5828499 28961544

[14] Johnson RM Fairman B Gilreath T Xuan Z Rothman EF Parnham T Past 15-year trends in adolescent marijuana use: Differences by race/ethnicity and sex. Drug Alcohol Depend (2015) 155:8–15. 10.1016/j.drugalcdep.2015.08.025 26361714 PMC4582007

[15] Hasin DS Sarvet AL Cerdá M Keyes KM Stohl M Galea S US adult illicit cannabis use, cannabis use disorder, and medical marijuana laws: 1991-1992 to 2012-2013. JAMA Psychiatry (2017) 74(6):579–88. 10.1001/jamapsychiatry.2017.0724 PMC5539836 28445557

[16] Zhu H Wu L-T Trends and correlates of cannabis-involved emergency department visits: 2004 to 2011. J Addict Med (2016) 10(6):429. 27574753 10.1097/ADM.0000000000000256 PMC5083207

[17] Saha TD Kerridge BT Goldstein RB Chou SP Zhang H Jung J Nonmedical prescription opioid use and DSM-5 nonmedical prescription opioid use disorder in the United States. J Clin Psychiatry (2016) 77(6):772. 10.4088/JCP.15m10386 27337416 PMC5555044

[18] Kerridge BT Chou SP Pickering RP Ruan WJ Huang B Jung J Changes in the prevalence and correlates of cocaine use and cocaine use disorder in the United States, 2001–2002 and 2012–2013. Addictive Behav (2019) 90:250–7. 10.1016/j.addbeh.2018.11.005 30471553

[19] Piper BJ Ogden CL Simoyan OM Chung DY Caggiano JF Nichols SD Trends in use of prescription stimulants in the United States and Territories, 2006 to 2016. PloS One (2018) 13(11):e0206100. 10.1371/journal.pone.0206100 30485268 PMC6261411

[20] Substance Abuse and Mental Health Services Administration Results from the 2017 National Survey on Drug Use and Health: Detailed Tables. Rockville, MD: Center for Behavioral Health Statistics and Quality (2018).

[21] Chen L-Y Crum RM Strain EC CalebAlexander G Kaufmann C Mojtabai R Prescriptions, nonmedical use, and emergency department visits involving prescription stimulants. J Clin Psychiatry (2016) 77(3):e297–304. 10.4088/JCP.14m09291 PMC5903919 26890573

[22] Saxena A Rubens M Das S Ramamoorthy V McGranaghan P Veledar E Abstract 17208: Trends in hospitalizations due to cocaine-induced acute Myocardial Infarction: Results From National Inpatient Sample, 2005-2014. Circulation (2018) 138(Suppl_1):A17208–8. 10.1161/circ.138.suppl_1.17208

[23] Anglin MD Hser Y-I Grella CE Drug addiction and treatment careers among clients in the Drug Abuse Treatment Outcome Study (DATOS). Psychol Addictive Behav (1997) 11(4):308–23. 10.1037/0893-164X.11.4.308

[24] Hubbard RL Marsden ME Rachal JV Harwood HJ Cavanaugh ER Ginzburg HM Drug abuse treatment: A national study of effectiveness. Chapel Hill, NC: University of North Carolina Press (1989).

[25] Simpson DD Joe GW Broome KM A national 5-year follow-up of treatment outcomes for cocaine dependence. Arch Gen Psychiatry (2002) 59(6):538–44. 10.1001/archpsyc.59.6.538 12044196

[26] Simpson DD Joe GW Brown BS Treatment retention and follow-up outcomes in the Drug Abuse Treatment Outcome Study (DATOS). Psychol Addictive Behav (1997) 11(4):294. 10.1037/0893-164X.11.4.294

[27] Connery HS Medication-assisted treatment of opioid use disorder: review of the evidence and future directions. Harvard Rev Psychiatry (2015) 23(2):63–75. 10.1097/HRP.0000000000000075 25747920

[28] Jones CM Campopiano M Baldwin G McCance-Katz E National and state treatment need and capacity for opioid agonist medication-assisted treatment. Am J Public Health (2015) 105(8):e55–63. 10.2105/AJPH.2015.302664 PMC4504312 26066931

[29] Saloner B Karthikeyan S Changes in substance abuse treatment use among individuals with opioid use disorders in the United States, 2004-2013. JAMA (2015) 314(14):1515–7. 10.1001/jama.2015.10345 26462001

[30] Volkow ND Frieden TR Hyde PS Cha SS Medication-assisted therapies—tackling the opioid-overdose epidemic. New Engl J Med (2014) 370(22):2063–6. 10.1056/NEJMp1402780 24758595

[31] Brezing CA Levin FR The current state of pharmacological treatments for cannabis use disorder and withdrawal. Neuropsychopharmacology (2018) 43(1):173–94. 10.1038/npp.2017.212 PMC5719115 28875989

[32] Chan B Kondo K Ayers C Freeman M Montgomery J Paynter R (2018). Pharmacotherapy for Stimulant Use Disorders: A Systematic Review. Washington, DC: Dept. of Veterans Affairs Retrieved from http://www.ncbi.nlm.nih.gov/books/NBK536789/. 30715830

[33] Prendergast M Podus D Finney J Greenwell L Roll J Contingency management for treatment of substance use disorders: a meta-analysis. Addiction (2006) 101(11):1546–60. 10.1111/j.1360-0443.2006.01581.x 17034434

[34] Stitzer ML Vandrey R Contingency management: utility in the treatment of drug abuse disorders. Clin Pharmacol Ther (2008) 83(4):644–7. 10.1038/sj.clpt.6100508 18305456

[35] Gates PJ Sabioni P Copeland J Foll BL Gowing L Psychosocial interventions for cannabis use disorder. Cochrane Database Syst Rev (2016)(5):CD005336. 10.1002/14651858.CD005336.pub4 27149547 PMC4914383

[36] Knapp WP Soares B Farrell M Silva de Lima M Psychosocial interventions for cocaine and psychostimulant amphetamines related disorders. Cochrane Database Syst Rev (2007) 3:CD003023. 10.1002/14651858.CD003023.pub2 17636713

[37] Barbosa PCR Mizumoto S Bogenschutz MP Strassman RJ Health status of ayahuasca users. Drug Test Anal (2012) 4(7–8):601–9. 10.1002/dta.1383 22761152

[38] Blum K Futterman SFL Pascarosa P Peyote, a potential ethnopharmacologic agent for alcoholism and other drug dependencies: possible biochemical rationale. Clin Toxicol (1977) 11(4):459–72. 10.3109/15563657708988210 201426

[39] Fábregas JM González D Fondevila S Cutchet M Fernández X Barbosa PCR Assessment of addiction severity among ritual users of ayahuasca. Drug Alcohol Depend (2010) 111(3):257–61. 10.1016/j.drugalcdep.2010.03.024 20554400

[40] Garcia-Romeu A Davis AK Erowid F Erowid E Griffiths RR Johnson MW Cessation and reduction in alcohol consumption and misuse after psychedelic use. J Psychopharmacol (2019) 33(9):1088–101. 10.1177/0269881119845793 31084460

[41] Halpern JH Hallucinogens in the treatment of alcoholism and other addictions. In: . Psychedelic medicine: New evidence for hallucinogenic substances as treatments, vol. 2 . Praeger Publishers/Greenwood Publishing Group: Westport, CT, US (2007). p. 1–14.

[42] Halpern JH Sherwood AR Passie T Blackwell KC Ruttenber AJ Evidence of health and safety in American members of a religion who use a hallucinogenic sacrament. Med Sci Monitor (2008) 14(8):SR15–22. 18668010

[43] Pisano VD Putnam NP Kramer HM Franciotti KJ Halpern JH Holden SC The association of psychedelic use and opioid use disorders among illicit users in the United States. J Psychopharmacol (2017) 31(5):606–13. 10.1177/0269881117691453 28196428

[44] Prue B Indigenous supports for recovery from alcoholism and drug abuse: The Native American Church. J Ethnic Cult Diversity Soc Work (2013) 22(3–4):271–87. 10.1080/15313204.2013.843138

[45] Rios MDde Grob CS Baker JR Hallucinogens and Redemption. J Psychoactive Drugs (2002) 34(3):239–48. 10.1080/02791072.2002.10399960 12422934

[46] Savage C McCabe OL Residential psychedelic (LSD) therapy for the narcotic addict: a controlled study. Arch Gen Psychiatry (1973) 28(6):808–14. 10.1001/archpsyc.1973.01750360040005 4575166

[47] Thomas G Lucas P Capler NR Tupper KW Martin G Ayahuasca-assisted therapy for addiction: results from a preliminary observational study in Canada. Curr Drug Abuse Rev (2013) 6(1):30–42. 10.2174/15733998113099990003 23627784

[48] Winkelman M Psychedelics as medicines for substance abuse rehabilitation: evaluating treatments with LSD, Peyote, Ibogaine and Ayahuasca. Curr Drug Abuse Rev (2014) 7(2):101–16. 10.2174/1874473708666150107120011 25563446

[49] Krebs TS Johansen P al-Ø Lysergic acid diethylamide (LSD) for alcoholism: Meta-analysis of randomized controlled trials. J Psychopharmacol (2012) 26(7):994–1002. 10.1177/0269881112439253 22406913

[50] Hendricks PS *Psilocybin treatment of cocaine use disorder* . In: . Presented at the College on Problems of Drug Dependence 80th Annual Scientific Meeting, San Diego, CA. Brentwood, TN: The College on Problems of Drug Dependence (2018).

[51] Johnson MW Psychedelics in the treatment of addiction. In: Grob CS, editor. Handbook of Medical Hallucinogens. Guilford Press: New York, NY (In Press).

[52] Roes T (2014). I Quit Smoking After an LSD Trip. New York, NY: Vice Media Retrieved July 25, 2019, from Vice website: https://www.vice.com/sv/article/5gkq4x/quit-smoking-with-lsd-876.

[53] Johnson MW Garcia-Romeu A Johnson PS Griffiths RR An online survey of tobacco smoking cessation associated with naturalistic psychedelic use. J Psychopharmacol (2017) 31(7):841–50. 10.1177/0269881116684335 PMC6753943 28095732

[54] Bogenschutz MP Forcehimes AA Pommy JA Wilcox CE Barbosa PCR Strassman RJ Psilocybin-assisted treatment for alcohol dependence: a proof-of-concept study. J Psychopharmacol (2015) 29(3):289–99. 10.1177/0269881114565144 25586396

[55] Johnson MW Garcia-Romeu A Griffiths RR Long-term follow-up of psilocybin-facilitated smoking cessation. Am J Drug Alcohol Abuse (2017) 43(1):55–60. 27441452 10.3109/00952990.2016.1170135 PMC5641975

[56] Etikan I Musa SA Alkassim RS Comparison of convenience sampling and purposive sampling. Am J Theor Appl Stat (2016) 5(1):1–4. 10.11648/j.ajtas.20160501.11

[57] Berman AH Bergman H Palmstierna T Schlyter F Evaluation of the drug use disorders identification test (DUDIT) in criminal justice and detoxification settings and in a Swedish population sample. Eur Addict Res (2005) 11(1):22–31. 10.1159/000081413 15608468

[58] Saunders JB Aasland OG Babor TF De la Fuente JR Grant M Development of the alcohol use disorders identification test (AUDIT): WHO collaborative project on early detection of persons with harmful alcohol consumption-II. Addiction (1993) 88(6):791–804. 10.1111/j.1360-0443.1993.tb02093.x 8329970

[59] Bradley KA DeBenedetti AF Volk RJ Williams EC Frank D Kivlahan DR AUDIT-C as a brief screen for alcohol misuse in primary care. Alcohol: Clin Exp Res (2007) 31(7):1208–17. 10.1111/j.1530-0277.2007.00403.x 17451397

[60] Bush K Kivlahan DR McDonell MB Fihn SD Bradley KA The AUDIT alcohol consumption questions (AUDIT-C): an effective brief screening test for problem drinking. Arch Internal Med (1998) 158(16):1789–95. 10.1001/archinte.158.16.1789 9738608

[61] Hudziak JJ Helzer JE Wetzel MW Kessel KB McGee B Janca A The use of the DSM-III-R Checklist for initial diagnostic assessments. Compr Psychiatry (1993) 34(6):375–83. 10.1016/0010-440x(93)90061-8 8131381

[62] Bohn MJ Krahn DD Staehler BA Development and initial validation of a measure of drinking urges in abstinent alcoholics. Alcohol: Clin Exp Res (1995) 19(3):600–6. 10.1111/j.1530-0277.1995.tb01554.x 7573780

[63] Barrett FS Johnson MW Griffiths RR Validation of the revised Mystical Experience Questionnaire in experimental sessions with psilocybin. J Psychopharmacol (2015) 29(11):1182–90. 10.1177/0269881115609019 PMC5203697 26442957

[64] Davis AK Barsuglia JP Lancelotta R Grant RM Renn E The epidemiology of 5-methoxy-N, N-dimethyltryptamine (5-MeO-DMT) use: Benefits, consequences, patterns of use, subjective effects, and reasons for consumption. J Psychopharmacol (2018) 32(7):779–92. 10.1177/0269881118769063 PMC6248886 29708042

[65] Liechti ME Dolder PC Schmid Y Alterations of consciousness and mystical-type experiences after acute LSD in humans. Psychopharmacology (2017) 234(9–10):1499–510. 10.1007/s00213-016-4453-0 PMC5420386 27714429

[66] MacLean KA Leoutsakos J-MS Johnson MW Griffiths RR Factor analysis of the mystical experience questionnaire: A study of experiences occasioned by the hallucinogen psilocybin. J Sci Stud Relig (2012) 51(4):721–37. 10.1111/j.1468-5906.2012.01685.x PMC3539773 23316089

[67] Griffiths RR Richards WA McCann U Jesse R Psilocybin can occasion mystical-type experiences having substantial and sustained personal meaning and spiritual significance. Psychopharmacology (2006) 187(3):268–83. 10.1007/s00213-006-0457-5 16826400

[68] Griffiths RR Johnson MW Richards WA Richards BD McCann U Jesse R Psilocybin occasioned mystical-type experiences: Immediate and persisting dose-related effects. Psychopharmacology (2011) 218(4):649–65. 10.1007/s00213-011-2358-5 PMC3308357 21674151

[69] IBM Corp. Released 2016 IBM SPSS Statistics for Windows, Version 24.0. Armonk, NY: IBM Corp.

[70] Muthén LK Muthén BO 1998–2017. Mplus User’s Guide. Muthén & Muthén: Los Angeles, CA (2017).

[71] Canagasaby A Vinson DC Screening for hazardous or harmful drinking using one or two quantity–frequency questions. Alcohol Alcohol (2005) 40(3):208–13. 10.1093/alcalc/agh156 15797883

[72] Garcia-Romeu A Griffiths R W Johnson M Psilocybin-occasioned mystical experiences in the treatment of tobacco addiction. Curr Drug Abuse Rev (2014a) 7(3):157–64. 10.2174/1874473708666150107121331 PMC4342293 25563443

[73] Griffiths RR Richards WA Johnson MW McCann UD Jesse R Mystical-type experiences occasioned by psilocybin mediate the attribution of personal meaning and spiritual significance 14 months later. J Psychopharmacol (2008) 22(6):621–32. 10.1177/0269881108094300 PMC3050654 18593735

[74] MacLean KA Johnson MW Griffiths RR Mystical experiences occasioned by the hallucinogen psilocybin lead to increases in the personality domain of openness. J Psychopharmacol (2011) 25(11):1453–61. 10.1177/0269881111420188 PMC3537171 21956378

[75] Halpern JH Pope HG Hallucinogen persisting perception disorder: What do we know after 50 years? Drug Alcohol Depend (2003) 69(2):109–19. 10.1016/S0376-8716(02)00306-X 12609692

[76] Gage SH Hickman M Zammit S Association between cannabis and psychosis: epidemiologic evidence. Biol Psychiatry (2016) 79(7):549–56. 10.1016/j.biopsych.2015.08.001 26386480

[77] Johnson MW Richards WA Griffiths RR Human hallucinogen research: guidelines for safety. J Psychopharmacol (2008) 22(6):603–20. 10.1177/0269881108093587 PMC3056407 18593734

[78] Barratt MJ Ferris JA Lenton S Hidden populations, online purposive sampling, and external validity: Taking off the blindfold. Field Methods (2015) 27(1):3–21.

[79] Topp L Barker B Degenhardt L The external validity of results derived from ecstasy users recruited using purposive sampling strategies. Drug Alcohol Depend (2004) 73(1):33–40. 14687957 10.1016/j.drugalcdep.2003.09.001

[80] Johnson MW Garcia-Romeu A Cosimano MP Griffiths RR Pilot study of the 5-HT2AR agonist psilocybin in the treatment of tobacco addiction. J Psychopharmacol (2014) 28(11):983–92. 10.1177/0269881114548296 PMC4286320 25213996

[81] Griffiths RR Johnson MW Carducci MA Umbricht A Richards WA Richards BD Psilocybin produces substantial and sustained decreases in depression and anxiety in patients with life-threatening cancer: a randomized double-blind trial. J Psychopharmacol (2016) 30(12):1181–97. 10.1177/0269881116675513 PMC5367557 27909165

[82] Grob CS Danforth AL Chopra GS Hagerty M McKay CR Halberstadt AL Pilot study of psilocybin treatment for anxiety in patients with advanced-stage cancer. Arch Gen Psychiatry (2011) 68(1):71–8. 10.1001/archgenpsychiatry.2010.116 20819978

[83] Ross S Bossis A Guss J Agin-Liebes G Malone T Cohen B Rapid and sustained symptom reduction following psilocybin treatment for anxiety and depression in patients with life-threatening cancer: A randomized controlled trial. J Psychopharmacol (2016) 30(12):1165–80. 10.1177/0269881116675512 PMC5367551 27909164

[84] Gasser P Holstein D Michel Y Doblin R Yazar-Klosinski B Passie T Safety and efficacy of lysergic acid diethylamide-assisted psychotherapy for anxiety associated with life-threatening diseases. J Nervous Ment Dis (2014) 202(7):513. 10.1097/NMD.0000000000000113 PMC4086777 24594678

[85] Carhart-Harris RL Bolstridge M Rucker J Day CM Erritzoe D Kaelen M Psilocybin with psychological support for treatment-resistant depression: an open-label feasibility study. Lancet Psychiatry (2016) 3(7):619–27. 10.1016/S2215-0366(16)30065-7 27210031

[86] Carhart-Harris RL Roseman L Bolstridge M Demetriou L Pannekoek JN Wall MB Psilocybin for treatment-resistant depression: FMRI-measured brain mechanisms. Sci Rep (2017) 7(1):13187. 29030624 10.1038/s41598-017-13282-7 PMC5640601

[87] Carhart-Harris RL Bolstridge M Day CMJ Rucker J Watts R Erritzoe DE Psilocybin with psychological support for treatment-resistant depression: six-month follow-up. Psychopharmacology (2018) 235(2):399–408. 29119217 10.1007/s00213-017-4771-x PMC5813086

[88] Palhano-Fontes F Barreto D Onias H Andrade KC Novaes MM Pessoa JA Rapid antidepressant effects of the psychedelic ayahuasca in treatment-resistant depression: a randomized placebo-controlled trial. Psychol Med (2019) 49(4):655–63. 10.1017/S0033291718001356 PMC6378413 29903051

[89] Galvão ACM Almeida RN Silva ES Freire FAM Palhano-Fontes F Onias H Cortisol modulation by ayahuasca in patients with treatment resistant depression and healthy controls. Front Psychiatry (2018) 9:185. 10.3389/fpsyt.2018.00185 29867608 PMC5952178

[90] Almeida RNde Galvão AC de M da Silva FS Silva EA dos S Palhano-Fontes F Maia-de-Oliveira JP Modulation of Serum Brain-Derived Neurotrophic Factor by a Single Dose of Ayahuasca: Observation From a Randomized Controlled Trial. Front Psychol (2019) 10:1–13. 10.3389/fpsyg.2019.01234 31231276 PMC6558429

[91] Majić T Schmidt TT Gallinat J Peak experiences and the afterglow phenomenon: when and how do therapeutic effects of hallucinogens depend on psychedelic experiences? J Psychopharmacol (2015) 29(3):241–53. 10.1177/0269881114568040 25670401

[92] Pahnke WN Kurland AA Unger S Savage C Grof S The experimental use of psychedelic (LSD) psychotherapy. Jama (1970) 212(11):1856–63. 10.1001/jama.1970.03170240060010 5467681

[93] Bogenschutz MP Johnson MW Classic hallucinogens in the treatment of addictions. Prog In Neuropsychopharmacol Biol Psychiatry (2016) 64:250–8. 10.1016/j.pnpbp.2015.03.002 25784600

[94] Bogenschutz MP Pommy JM Therapeutic mechanisms of classic hallucinogens in the treatment of addictions: from indirect evidence to testable hypotheses. Drug Test Anal (2012) 4(7–8):543–55. 10.1002/dta.1376 22761106

[95] Johnson MW Bickel WK Baker F Moore BA Badger GJ Budney AJ Delay discounting in current and former marijuana-dependent individuals. Exp Clin Psychopharmacol (2010) 18(1):99–107. 10.1037/a0018333 20158299 PMC2874198

[96] Ross S Serotonergic hallucinogens and emerging targets for addiction pharmacotherapies. Psychiatr Clinics (2012) 35(2):357–74. 10.1016/j.psc.2012.04.002 22640760

[97] Garcia-Romeu A Griffiths R W Johnson M Psilocybin-occasioned mystical experiences in the treatment of tobacco addiction. Curr Drug Abuse Rev (2014b) 7(3):157–64. 10.2174/1874473708666150107121331 PMC4342293 25563443

[98] Ly C Greb AC Cameron LP Wong JM Barragan EV Wilson PC Psychedelics Promote Structural and Functional Neural Plasticity. Cell Rep (2018) 23(11):3170–82. 10.1016/j.celrep.2018.05.022 PMC6082376 29898390

[99] Flanagan TW Nichols CD Psychedelics as anti-inflammatory agents. Int Rev Psychiatry (2018) 30(4):363–75. 10.1080/09540261.2018.1481827 30102081

[100] Cata-Preta EG Serra YA Moreira-Junior E da C Reis HS Kisaki ND Ayahuasca and its DMT-and β-carbolines–containing ingredients block the expression of ethanol-induced conditioned place preference in mice: Role of the treatment environment. Front In Pharmacol (2018) 9:1–14. 10.3389/fphar.2018.00561 PMC5986901 29896106

[101] Oppong-Damoah A Curry KE Blough BE Rice KC Murnane KS Effects of the synthetic psychedelic 2, 5-dimethoxy-4-iodoamphetamine (DOI) on ethanol consumption and place conditioning in male mice. Psychopharmacology (2019) 236(12):3567–78. 10.1007/s00213-019-05328-7 PMC6895420 31309240

[102] Godinho AF Silva MC Kawashima JD Horta DF Anselmo F De Fraia D Ayahuasca modifies amphetamine self ingestion and modifies anxiety and locomotor activity in adolescent rats. Electron J Biol (2017) 13(2):159–65.

[103] Nichols DE Frescas S Improvements to the synthesis of psilocybin and a facile method for preparing the O-acetyl prodrug of psilocin. Synthesis (1999) 1999(06):935–8.

[104] Vargas-Perez H Grieder TE Ting-A-Kee R Maal-Bared G Chwalek M van der Kooy D A single administration of the hallucinogen, 4-acetoxy-dimethyltryptamine, prevents the shift to a drug-dependent state and the expression of withdrawal aversions in rodents. Eur J Neurosci (2017) 45(11):1410–7. 10.1111/ejn.13572 28378435

[105] Johnson MW Griffiths RR Hendricks PS Henningfield JE The abuse potential of medical psilocybin according to the 8 factors of the Controlled Substances Act. Neuropharmacology (2018) 142:143–66. 10.1016/j.neuropharm.2018.05.012. PMC6791528 29753748

[106] Noorani T Making psychedelics into medicines: The politics and paradoxes of medicalization. J Psychedelic Stud (2019) 1–6. 10.1556/2054.2019.018

[107] Sellers EM Leiderman DB Psychedelic drugs as therapeutics: no illusions about the challenges. Clin Pharmacol Ther (2018) 103(4):561–4. 10.1002/cpt.776 28836272

