# Supplementary material for: Persisting Reductions in Cannabis, Opioid, and Stimulant Misuse After Naturalistic Psychedelic Use: An Online Survey
Source: Front Psychiatry. 2020 Jan 22;10:955. doi: 10.3389/fpsyt.2019.00955 (PMC6987443; doi:10.3389/fpsyt.2019.00955)
Supplement: Supplementary file 1 [file DataSheet_1.pdf]

Johns Hopkins Survey on Psychedelic Use and Addiction

Thank you for your interest in this questionnaire study on the potential relationship between psychedelic experiences and reduction or cessation of addictive behaviors. This research is being conducted by scientists at Johns Hopkins University School of Medicine and has been approved by the Johns Hopkins University Institutional Review Board (IRB).

Our Previous Research

Our research team has conducted surveys characterizing positive and challenging experiences after taking psychedelics in nonlaboratory settings, as well as instances in which taking a psychedelic was associated with a cessation or reduction in cigarette smoking. We've also studied both positive and psychologically difficult experiences, as well as headaches, after giving psilocybin to volunteers in our laboratory.

Purpose of This Study

The goal of this survey is to learn more about whether use of psychedelic drugs may be associated with reduction or cessation of addictive behaviors. We want to characterize people's experiences in non-laboratory settings in which taking a psychedelic may have led to reducing or quitting alcohol or another drug. For the purposes of this survey, we will be asking specifically about individuals who have quit or reduced using alcohol, opioids (heroin, morphine, hydrocodone, oxycodone, codeine, Oxycontin, Vicodin, Percocet, etc.), stimulants (cocaine, methamphetamine, amphetamine, methylphenidate, Adderall, Ritalin, Dexedrine, etc.), or cannabis after a psychedelic experience. For example, if you had an alcohol abuse problem that improved after a psychedelic experience, you would be a good candidate for this study. For the purposes of this survey, a psychedelic experience can refer to an experience with psilocybin (magic) mushrooms, LSD, morning glory seeds, mescaline, peyote cactus, San Pedro cactus, DMT, Ayahuasca, or MDMA (ecstasy).

Inclusion Criteria

You are invited to participate in this survey if you fulfill all of the four criteria listed below.

1) You are at least 18 years old.
2) You read and write English fluently.
3) You have taken a psychedelic drug outside of an official university or hospital study, and
4) You experienced a reduction in addictive behavior (alcohol or other drug use) after taking a psychedelic.

What the Study Entails

Participation in this study involves filling out an online survey that will take approximately 35 minutes. You will be required to complete the survey in one sitting. At the conclusion of the main survey you will be invited to participate in an optional extension of the survey that will ask some more detailed questions and take about 10 minutes to complete in addition to the main survey. *These additional questions are completely optional, and you are free to accept or decline to respond to these as you choose.*

Why should I participate?

We believe that this study is scientifically important. We would like you to participate because we need to collect responses from many different people. You may find this survey interesting. There is also a chance that you will be bored. Although there is no monetary compensation for participation, you will be making a unique and important contribution to science.

Is my participation confidential?

Yes. We do not collect identifying information such as your name, email address, or IP address. Your anonymous responses will be seen and analyzed by Johns Hopkins staff or representatives. To further protect the confidentiality of participants, the results of most questions will be presented in aggregate. We may quote from your textual responses. However, if you provide specific identifying information, we will edit your responses to protect your confidentiality.

What kind of information will I be providing?

At the beginning of the survey, you will be asked questions about your background, alcohol and drug use history. Psychological questionnaires are also included to assess relationships between constructs of interest like personality, and their relevance to addiction and/or drug use. Finally, we will be collecting detailed information about your alcohol or other drug use patterns before and after the psychedelic experience, as well as information on the nature of your psychedelic experience, and why it may have led to a change in your alcohol or other drug use.

What will become of the results from this study?

The researchers intend to publish the results from this study in the scientific literature and to present results at scientific meetings. We will also make the results publicly available by posting a notice of any scholarly publications on the website of the Council on Spiritual Practices (www.csp.org).

Your responses will not be used in this study if you do not complete the survey.

Your participation in this study is voluntary. Your completing the survey will serve as your consent to be in the study. Even after you begin the survey, you may stop answering the questions at any time. If you stop early, none of your responses will be used. At the end of the main survey, you will have a final opportunity to consent, or not, to all of your responses being submitted. At that time you will be invited to continue on to some additional questions that will assess your decision making process. These extra questions are optional. If you begin them then decide to quit before finishing, your responses to the earlier parts of the survey will already be recorded, and will still be used for the research study.

Johns Hopkins Survey on Psychedelic Use and Addiction

How do I start?

You can begin the survey by clicking 'Begin survey' at the bottom of this page. This survey will take approximately 35 minutes, with the option to respond to some additional questions at the end. You will be required to complete the survey in one sitting.

It is important that you complete the survey only once, and that you answer each question honestly and seriously. If you are not ready to complete the survey now, please return to this page at a time that is convenient for you.

You can exit the survey at any time by clicking the link in the upper right corner of your screen. If you exit the survey early, your responses will not be used.

During the survey, **please do not hit the "back" button** on your internet browser as it may erase your answers or prematurely terminate your session.

By clicking 'Begin survey' below, you affirm that:

- you have read the information above,
- you voluntarily agree to participate,
- you are at least 18 years old,
- you read and write English fluently,
- you have taken a psychedelic substance outside of an official university or hospital study, and
- you experienced a reduction in addictive behavior (alcohol or other drug use) after taking a psychedelic.

1. Please click below to begin or exit the survey.

◯ Begin survey

◯ Exit Survey

2. Have you completed this survey before?

◯ Yes

◯ No

3. Do you read, write, and speak English fluently?

◯ Yes

◯ No

4. Are you 18 years old or older?

◯ Yes

◯ No

72. What is your sex?

- ○ Male
- ○ Female

73. What is your gender?

- ○ Female
- ○ Male
- ○ Transgender (female to male)
- ○ Transgender (male to female)
- ○ Other (please specify)

74. What is your current age?

75. What is the highest level of education you have completed?

- ○ Some high school or less
- ○ High school diploma or equivalent
- ○ Some college
- ○ College degree
- ○ Some graduate/professional school
- ○ Graduate/professional degree

76. What is your marital status?

- ○ Single
- ○ In a committed relationship (not married)
- ○ Married
- ○ Separated
- ○ Divorced
- ○ Widowed

77. In what country do you currently live?

78. In which state do you currently reside?

79. Which of the following best describes your racial background?

- ◯ American Indian/Alaska Native (a person having origins in any of the original peoples of North, Central, and South America)
- ◯ Asian (a person having origins in any of the original peoples of the Far East, Southeast Asia, or the Indian subcontinent)
- ◯ Native Hawaiian or Pacific Islander (a person having origins in any of the original peoples of Hawaii, Guam, Samoa, or other Pacific Islands)
- ◯ Black or African American (a person having origins in any of the Black racial groups of Africa)
- ◯ White (a person having origins in any of the original peoples of Europe, the Middle East, or North Africa)
- ◯ Some other race
- ◯ More than one race
- ◯ Prefer not to answer

80. Are you Hispanic or Latino? ('Hispanic' or 'Latino' refers to a person of Cuban, Mexican, Puerto Rican, South or Central American, or other Spanish culture or origin, regardless of race).

- ◯ Yes
- ◯ No
- ◯ Prefer not to answer

81. What is your total annual household income (in US dollars)?

- ◯ Less than $10,000
- ◯ $10,000 to $19,999
- ◯ $20,000 to $29,999
- ◯ $30,000 to $39,999
- ◯ $40,000 to $49,999
- ◯ $50,000 to $59,999
- ◯ $60,000 to $69,999
- ◯ $70,000 to $79,999
- ◯ $80,000 to $89,999
- ◯ $90,000 to $99,999
- ◯ $100,000 to $149,999
- ◯ $150,000 to $249,999
- ◯ $250,000 or more

82. How would you classify your current occupation?

- ◯ Architecture and Engineering
- ◯ Arts, Design, Entertainment, Sports, and Media
- ◯ Building and Grounds Cleaning and Maintenance
- ◯ Business and Financial Operations
- ◯ Community and Social Service
- ◯ Computer and Mathematical
- ◯ Construction and Extraction
- ◯ Education, Training, and Library
- ◯ Farming, Fishing, and Forestry
- ◯ Food Preparation and Serving Related
- ◯ Healthcare Practitioners and Technical
- ◯ Healthcare Support
- ◯ Installation, Maintenance, and Repair
- ◯ Legal
- ◯ Life, Physical, and Social Science
- ◯ Management
- ◯ Office and Administrative Support
- ◯ Personal Care and Service
- ◯ Production
- ◯ Protective Service
- ◯ Sales and Related
- ◯ Student
- ◯ Transportation and Material Moving
- ◯ Unemployed
- ◯ Other (please specify)

83. Have you struggled with anxiety, depression, a substance dependence disorder, or another psychiatric disorder of some kind (e.g., schizophrenia) at any point in your life?

- ◯ Yes
- ◯ No

84. Please mark all psychiatric disorders you have struggled with or been diagnosed with.

- [ ] Anxiety Disorder (Social Anxiety, Panic, Phobia, PTSD, etc.)
- [ ] Eating Disorder (Anorexia, Bulimia, etc.)
- [ ] Impulse Control Disorder (Pyromania, Compulsive Gambling, etc.)
- [ ] Mood Disorder (Depression, Mania, Bipolar, etc.)
- [ ] Personality Disorder (Paranoid, Avoidant, Borderline, etc.)
- [ ] Psychotic Disorder (Schizophrenia, Schizoaffective, etc.)
- [ ] Substance-Related Disorder (Alcohol or Drug Dependence)
- [ ] Other (please specify)

85. Do you use any of the following medications or drugs regularly (at least once a week)? Select all that apply.

- [ ] alcohol
- [ ] “bath salt” drug products (MDPV, mephedrone, methylone, etc.)
- [ ] benzodiazepines (Xanax, Valium, Klonopin, etc.)
- [ ] caffeine (coffee, tea, soda, dark chocolate, Vivarin, No-Doz, etc.)
- [ ] cannabis (marijuana)
- [ ] cocaine
- [ ] dextromethorphan (DXM)
- [ ] hallucinogens (psilocybin, LSD, DMT, Ayahuasca, mescaline, etc.)
- [ ] hash oil, dabs, THC oil, cannabis extract oils, wax, etc.
- [ ] ketamine
- [ ] MDMA (ecstasy, Molly)
- [ ] methamphetamine
- [ ] opioids (heroin, morphine, hydrocodone, oxycodone, codeine, Oxycontin, Vicodin, Percocet, etc.)
- [ ] prescription antidepressants (Celexa, Cymbalta, Effexor, Prozac, Zoloft, etc.)
- [ ] prescription stimulants (amphetamine, methylphenidate, Adderall, Ritalin, Dexedrine, etc.)
- [ ] synthetic marijuana (K2, Spice, etc.)
- [ ] tobacco (nicotine)
- [ ] I do not use any of these medications or drugs regularly.
- [ ] other (please specify)

86. On approximately how many separate occasions have you taken each of these substances during your lifetime?

	# of uses
alcohol	
ayahuasca	
“bath salt” drug products (MDPV, mephedrone, methylone, etc.)	
cannabis (marijuana)	
cocaine	
DMT (pure compound)	
hash oil, dabs, THC oil, cannabis extract oils, wax, etc.	
LSD	
MDMA (ecstasy, Molly)	
mescaline (pure compound)	
methamphetamine	
morning glory seeds	
opioids (heroin, morphine, hydrocodone, oxycodone, codeine, Oxycontin, Vicodin, Percocet, etc.)	
peyote cactus	
presecription stimulants (Adderall, Ritalin, Dexedrine, etc.)	
psilocybin mushrooms	
San Pedro cactus	

87. How old were you when you first took a psychedelic substance? For the purposes of this survey, please count only the following substances: psilocybin (magic) mushrooms, LSD, morning glory seeds, mescaline, peyote cactus, San Pedro cactus, DMT, Ayahuasca, or MDMA (ecstasy, Molly).

88. When did you last use a psychedelic substance? For the purposes of this survey, please count only the following substances: psilocybin (magic) mushrooms, LSD, morning glory seeds, mescaline, peyote cactus, San Pedro cactus, DMT, Ayahuasca, or MDMA (ecstasy, Molly).

- In the past 24 hours
- In the past week
- In the past month
- In the past year
- In the past 5 years
- In the past 10 years
- More than 10 years ago

89. How much distress or dysfunction have you experienced during your lifetime as a result of your use of the following drugs? By distress or dysfunction, we mean any significant problems in your personal relationships, work, or home life that may have been caused by use of these substances.

	Distress / Dysfunction Rating
alcohol	
ayahuasca	
"bath salt" drug products (MDPV, mephedrone, methylone, etc.)	
cannabis (marijuana)	
cocaine	
DMT (pure compound)	
hash oil, dabs, THC oil, cannabis extract oils, wax, etc.	
LSD	
MDMA (ecstasy, Molly)	
mescaline (pure compound)	
methamphetamine	
morning glory seeds	
opioids (heroin, morphine, hydrocodone, oxycodone, codeine, Oxycontin, Vicodin, Percocet, etc.)	
peyote cactus	
presecription stimulants (Adderall, Ritalin, Dexedrine, etc.)	
psilocybin mushrooms	
San Pedro cactus	

For the next portion of the survey, we will be asking you to focus on a specific substance you deemed to be most problematic in your life (either alcohol, opioids, stimulants, or cannabis), and a particular psychedelic experience that seemed to help you stop or reduce your use of that substance.

If your psychedelic experience helped you to cut down or quit using several substances, you will be able to comment on that in later sections. However, for now we would like you to <u>focus on just one of these substances that you were able to reduce or stop using after a psychedelic experience: alcohol, opioids, stimulants, or cannabis.</u>

90. Which of these substances did you reduce or stop using after a psychedelic experience?

- ◯ alcohol
- ◯ opioids (heroin, morphine, hydrocodone, oxycodone, codeine, Oxycontin, Vicodin, Percocet, etc.)
- ◯ stimulants (cocaine, methamphetamine, amphetamine, methylphenidate, Adderall, Ritalin, Dexedrine, etc.)
- ◯ cannabis (marijuana)

For this section we would like you to <u>focus on just one psychedelic experience that you deemed to be most important in affecting a decrease in your alcohol use.</u> If you reduced your alcohol use after multiple psychedelic experiences, you will be able to provide more information on the substances, doses, and sequence of events in later sections.

91. How long ago did the psychedelic experience that led to your quitting or reducing alcohol occur?

- ◯ Less than 1 week
- ◯ 1 - 2 weeks
- ◯ 3 - 4 weeks
- ◯ 1 - 3 months
- ◯ 4 - 6 months
- ◯ 7 - 12 months
- ◯ 1 - 2 years
- ◯ 3 - 5 years
- ◯ 6 - 10 years
- ◯ 11 - 20 years
- ◯ More than 20 years

92. Please characterize your alcohol consumption after this psychedelic experience.

- ◯ Stopped drinking completely since the experience (Total abstinence from alcohol).
- ◯ Greatly reduced drinking since the experience.
- ◯ Reduced drinking somewhat since the experience.
- ◯ Stopped drinking completely for a period of time, then returned to drinking the same amount as before.
- ◯ Reduced drinking for a period of time, then returned to drinking the same amount as before.

Other (please specify)

93. How long did your alcohol reduction or abstinence last?

- Less than 1 week
- 1 - 2 weeks
- 3 - 4 weeks
- 1 - 3 months
- 4 - 6 months
- 7 - 12 months
- 1 - 2 years
- 3 - 5 years
- 6 - 10 years
- 11 - 20 years
- More than 20 years

94. Which substance led to the psychedelic experience associated with your quitting or reducing your alcohol use?

- psilocybin mushrooms
- LSD
- morning glory seeds
- mescaline (pure compound)
- peyote cactus
- San Pedro cactus
- DMT (pure compound)
- Ayahuasca
- MDMA (ecstasy, Molly)

Other (please specify)

95. What was the approximate dose ingested to the best of your knowledge?

- Very low dose
- Low dose
- Moderate dose
- High dose
- Very high dose

96. How old were you when this experience took place?

97. Where did this experience take place? (Check all that apply):

- [ ] At home
- [ ] At a party
- [ ] In a public place (e.g., shopping mall, movie theater, etc.)
- [ ] At a concert or festival
- [ ] Outdoors in nature
- [ ] In a religious or spiritual setting (e.g., ceremony, retreat, etc.)

Other (please specify)

98. Were you alone or with other people during the majority of this psychedelic session?

- ◯ Alone
- ◯ With 1 other person
- ◯ With a few other people (2 - 5)
- ◯ With a small group of people (6 - 20)
- ◯ At a medium-sized gathering (21 - 100)
- ◯ At a large gathering (more than 100 people)

99. What was your intention in this particular instance for taking a psychedelic? (Check all that apply):

- [ ] I took a psychedelic only because other people were, but I did not have a serious intention.
- [ ] Curiosity without any other serious intention.
- [ ] Recreational (e.g., to enjoy the experience, which may or may not involve a social recreational event such as a concert).
- [ ] A serious intention for psychological self-exploration (e.g., resolve a personal issue or to increase self-understanding).
- [ ] A serious intention to explore spirituality or the sacred (e.g., to contemplate God, as you understand that word, or the nature of ultimate reality, and so on).
- [ ] A premeditated intention to quit or reduce drinking alcohol.
- [ ] Other (please specify)

100. After your psychedelic experience, did you use any other methods to help maintain alcohol abstinence or reduction? (Check all that apply):

- ☐ None
- ☐ Detoxification at a treatment center
- ☐ Professional counseling or psychotherapy
- ☐ Counseling provided by a “Quit-line” telephone number DEL
- ☐ Counseling provided by a website (for example, WebMD)
- ☐ Hypnosis DEL
- ☐ Acupuncture DEL
- ☐ Support group DEL (Alcoholics Anonymous, etc.)
- ☐ Self-help manuals or books DEL
- ☐ Disulfiram (Antabuse) DEL
- ☐ Naltrexone (Revia)
- ☐ Acamprosate (Campral)
- ☐ Spiritual / religious practice (Church attendance, meditation, etc.)
- ☐ Other (please specify)

101. Please provide a brief description of the psychedelic experience that led to your quitting or reducing your alcohol use.

Looking back on the entirety of the psychedelic session that occurred prior to your alcohol reduction or cessation, please rate the degree to which at any time during that session you experienced the following phenomena. Answer each question according to your feelings, thoughts, and experiences at the time of the psychedelic session.

102. Loss of your usual sense of time.

- 0 – none; not at all
- 1 – so slight cannot decide
- 2 – slight
- 3 – moderate
- 4 – strong (equivalent in degree to any other strong experience)
- 5 – extreme (more than any other time in my life and stronger than 4)

103. Experience of amazement.

- 0 – none; not at all
- 1 – so slight cannot decide
- 2 – slight
- 3 – moderate
- 4 – strong (equivalent in degree to any other strong experience)
- 5 – extreme (more than any other time in my life and stronger than 4)

104. Sense that the experience cannot be described adequately in words.

- 0 – none; not at all
- 1 – so slight cannot decide
- 2 – slight
- 3 – moderate
- 4 – strong (equivalent in degree to any other strong experience)
- 5 – extreme (more than any other time in my life and stronger than 4)

105. Gain of insightful knowledge experienced at an intuitive level.

- 0 – none; not at all
- 1 – so slight cannot decide
- 2 – slight
- 3 – moderate
- 4 – strong (equivalent in degree to any other strong experience)
- 5 – extreme (more than any other time in my life and stronger than 4)

106. Feeling that you experienced eternity or infinity.

- 0 – none; not at all
- 1 – so slight cannot decide
- 2 – slight
- 3 – moderate
- 4 – strong (equivalent in degree to any other strong experience)
- 5 – extreme (more than any other time in my life and stronger than 4)

107. Experience of oneness or unity with objects and/or persons perceived in your surroundings.

- 0 – none; not at all
- 1 – so slight cannot decide
- 2 – slight
- 3 – moderate
- 4 – strong (equivalent in degree to any other strong experience)
- 5 – extreme (more than any other time in my life and stronger than 4)

108. Loss of your usual sense of space.

- ◯ 0 – none; not at all
- ◯ 1 – so slight cannot decide
- ◯ 2 – slight
- ◯ 3 – moderate
- ◯ 4 – strong (equivalent in degree to any other strong experience)
- ◯ 5 – extreme (more than any other time in my life and stronger than 4)

109. Feelings of tenderness and gentleness.

- ◯ 0 – none; not at all
- ◯ 1 – so slight cannot decide
- ◯ 2 – slight
- ◯ 3 – moderate
- ◯ 4 – strong (equivalent in degree to any other strong experience)
- ◯ 5 – extreme (more than any other time in my life and stronger than 4)

110. Certainty of encounter with ultimate reality (in the sense of being able to "know" and "see" what is really real at some point during your experience).

- ◯ 0 – none; not at all
- ◯ 1 – so slight cannot decide
- ◯ 2 – slight
- ◯ 3 – moderate
- ◯ 4 – strong (equivalent in degree to any other strong experience)
- ◯ 5 – extreme (more than any other time in my life and stronger than 4)

111. Feeling that you could not do justice to your experience by describing it in words.

- ◯ 0 – none; not at all
- ◯ 1 – so slight cannot decide
- ◯ 2 – slight
- ◯ 3 – moderate
- ◯ 4 – strong (equivalent in degree to any other strong experience)
- ◯ 5 – extreme (more than any other time in my life and stronger than 4)

112. Loss of usual awareness of where you were.

- 0 – none; not at all
- 1 – so slight cannot decide
- 2 – slight
- 3 – moderate
- 4 – strong (equivalent in degree to any other strong experience)
- 5 – extreme (more than any other time in my life and stronger than 4)

113. Feelings of peace and tranquility.

- 0 – none; not at all
- 1 – so slight cannot decide
- 2 – slight
- 3 – moderate
- 4 – strong (equivalent in degree to any other strong experience)
- 5 – extreme (more than any other time in my life and stronger than 4)

114. Sense of being "outside of" time, beyond past and future.

- 0 – none; not at all
- 1 – so slight cannot decide
- 2 – slight
- 3 – moderate
- 4 – strong (equivalent in degree to any other strong experience)
- 5 – extreme (more than any other time in my life and stronger than 4)

115. Freedom from the limitations of your personal self and feeling a unity or bond with what was felt to be greater than your personal self.

- 0 – none; not at all
- 1 – so slight cannot decide
- 2 – slight
- 3 – moderate
- 4 – strong (equivalent in degree to any other strong experience)
- 5 – extreme (more than any other time in my life and stronger than 4)

116. Sense of being at a spiritual height.

- 0 – none; not at all
- 1 – so slight cannot decide
- 2 – slight
- 3 – moderate
- 4 – strong (equivalent in degree to any other strong experience)
- 5 – extreme (more than any other time in my life and stronger than 4)

117. Experience of pure being and pure awareness (beyond the world of sense impressions).

- 0 – none; not at all
- 1 – so slight cannot decide
- 2 – slight
- 3 – moderate
- 4 – strong (equivalent in degree to any other strong experience)
- 5 – extreme (more than any other time in my life and stronger than 4)

118. Experience of ecstasy.

- 0 – none; not at all
- 1 – so slight cannot decide
- 2 – slight
- 3 – moderate
- 4 – strong (equivalent in degree to any other strong experience)
- 5 – extreme (more than any other time in my life and stronger than 4)

119. Experience of the insight that “all is One”.

- 0 – none; not at all
- 1 – so slight cannot decide
- 2 – slight
- 3 – moderate
- 4 – strong (equivalent in degree to any other strong experience)
- 5 – extreme (more than any other time in my life and stronger than 4)

120. Being in a realm with no space boundaries.

- 0 – none; not at all
- 1 – so slight cannot decide
- 2 – slight
- 3 – moderate
- 4 – strong (equivalent in degree to any other strong experience)
- 5 – extreme (more than any other time in my life and stronger than 4)

121. Experience of oneness in relation to an “inner world” within.

- 0 – none; not at all
- 1 – so slight cannot decide
- 2 – slight
- 3 – moderate
- 4 – strong (equivalent in degree to any other strong experience)
- 5 – extreme (more than any other time in my life and stronger than 4)

122. Sense of reverence.

- 0 – none; not at all
- 1 – so slight cannot decide
- 2 – slight
- 3 – moderate
- 4 – strong (equivalent in degree to any other strong experience)
- 5 – extreme (more than any other time in my life and stronger than 4)

123. Experience of timelessness.

- 0 – none; not at all
- 1 – so slight cannot decide
- 2 – slight
- 3 – moderate
- 4 – strong (equivalent in degree to any other strong experience)
- 5 – extreme (more than any other time in my life and stronger than 4)

124. You are convinced now, as you look back on your experience, that in it you encountered ultimate reality (i.e., that you “knew” and “saw” what was really real).

- 0 – none; not at all
- 1 – so slight cannot decide
- 2 – slight
- 3 – moderate
- 4 – strong (equivalent in degree to any other strong experience)
- 5 – extreme (more than any other time in my life and stronger than 4)

125. Feeling that you experienced something profoundly sacred and holy.

- 0 – none; not at all
- 1 – so slight cannot decide
- 2 – slight
- 3 – moderate
- 4 – strong (equivalent in degree to any other strong experience)
- 5 – extreme (more than any other time in my life and stronger than 4)

126. Awareness of the life or living presence in all things.

- 0 – none; not at all
- 1 – so slight cannot decide
- 2 – slight
- 3 – moderate
- 4 – strong (equivalent in degree to any other strong experience)
- 5 – extreme (more than any other time in my life and stronger than 4)

127. Experience of the fusion of your personal self into a larger whole.

- 0 – none; not at all
- 1 – so slight cannot decide
- 2 – slight
- 3 – moderate
- 4 – strong (equivalent in degree to any other strong experience)
- 5 – extreme (more than any other time in my life and stronger than 4)

128. Sense of awe or awesomeness.

- 0 – none; not at all
- 1 – so slight cannot decide
- 2 – slight
- 3 – moderate
- 4 – strong (equivalent in degree to any other strong experience)
- 5 – extreme (more than any other time in my life and stronger than 4)

129. Experience of unity with ultimate reality.

- 0 – none; not at all
- 1 – so slight cannot decide
- 2 – slight
- 3 – moderate
- 4 – strong (equivalent in degree to any other strong experience)
- 5 – extreme (more than any other time in my life and stronger than 4)

130. Feeling that it would be difficult to communicate your own experience to others who have not had similar experiences.

- 0 – none; not at all
- 1 – so slight cannot decide
- 2 – slight
- 3 – moderate
- 4 – strong (equivalent in degree to any other strong experience)
- 5 – extreme (more than any other time in my life and stronger than 4)

131. Feelings of joy.

- 0 – none; not at all
- 1 – so slight cannot decide
- 2 – slight
- 3 – moderate
- 4 – strong (equivalent in degree to any other strong experience)
- 5 – extreme (more than any other time in my life and stronger than 4)

The purpose of the following questions is to obtain your retrospective assessment of the psychedelic experience that led to your alcohol use reduction, <u>and your contemplation of that experience.</u> For all questions on this page, please choose the highest rating that applies.

132. How personally meaningful were the psychedelic experience that led to your alcohol use reduction, and your contemplation of that experience?

- ◯ No more than routine, everyday experiences
- ◯ Similar to meaningful experiences that occur on average once or more a week
- ◯ Similar to meaningful experiences that occur on average once a month
- ◯ Similar to meaningful experiences that occur on average once a year
- ◯ Similar to meaningful experiences that occur on average once every 5 years
- ◯ Among the 10 most meaningful experiences of my life
- ◯ Among the 5 most meaningful experiences of my life
- ◯ The single most meaningful experience of my life

133. Indicate the degree to which the psychedelic experience that led to your alcohol use reduction, and your contemplation of that experience, were spiritually significant to you.

- ◯ Not at all
- ◯ Slightly
- ◯ Moderately
- ◯ Very much
- ◯ Among the 5 most spiritually significant experiences of my life
- ◯ The single most spiritually significant experience of my life

134. How psychologically challenging was the most psychologically challenging portion of the psychedelic experience that led to your alcohol use reduction?

- ◯ No more than routine, everyday experiences
- ◯ Similar to difficult or challenging experiences that occur on average once or more a week
- ◯ Similar to difficult or challenging experiences that occur on average once a month
- ◯ Similar to difficult or challenging experiences that occur on average once a year
- ◯ Similar to difficult or challenging experiences that occur on average once every 5 years
- ◯ Among the 10 most difficult or challenging experiences of my life
- ◯ Among the 5 most difficult or challenging experiences of my life
- ◯ The single most difficult or challenging experience of my life

135. How psychologically insightful to you were the psychedelic experience that led to your alcohol use reduction, and your contemplation of that experience?

- ◯ No more than routine, everyday psychologically insightful experiences
- ◯ Similar to psychologically insightful experiences that occur on average once or more a week
- ◯ Similar to psychologically insightful experiences that occur on average once a month
- ◯ Similar to psychologically insightful experiences that occur on average once a year
- ◯ Similar to psychologically insightful experiences that occur on average once every 5 years
- ◯ Among the 10 most psychologically insightful experiences of my life
- ◯ Among the 5 most psychologically insightful experiences of my life
- ◯ The single most psychologically insightful experience of my life

136. Do you believe that the psychedelic experience that led to your alcohol use reduction, and your contemplation of that experience, have led to change in your current sense of personal well-being or life satisfaction?

- ◯ Strong positive change that I consider desirable
- ◯ Moderate positive change that I consider desirable
- ◯ Slight positive change that I consider desirable
- ◯ No change
- ◯ Slight negative change that I consider undesirable
- ◯ Moderate negative change that I consider undesirable
- ◯ Strong negative change that I consider undesirable

137. Please rank items in order of their importance to your psychedelic-associated alcohol abstinence or reduction.

Rank	Item	
☐	Strengthening your belief in your own ability to quit.	☐ Not applicable
☐	Reducing stress involved with quitting.	☐ Not applicable
☐	Reframing quitting as a spiritual task.	☐ Not applicable
☐	Changing life priorities or values, such that drinking was no longer more important than quitting.	☐ Not applicable
☐	Changing your orientation toward the future, so that long-term benefits outweighed immediate desires.	☐ Not applicable
☐	Increasing space between the experience of craving and taking action.	☐ Not applicable

138. Please describe any additional reasons (not listed above) that your psychedelic experience may have contributed to your quitting or reducing alcohol.

139. Did you experience any other behavioral changes after this psychedelic session? Check all that apply, and please provide any relevant details in the Comments box below.

- ☐ None
- ☐ Reduced or stopped using other drugs
- ☐ Started using other drugs more often / heavily
- ☐ Improved diet / nutrition
- ☐ Worsened diet / nutrition
- ☐ Increased physical activity / exercise
- ☐ Decreased physical activity / exercise
- ☐ Improved relationships with others
- ☐ Worsened relationships with others
- ☐ Improvements in career / work life
- ☐ Worsening of career / work life

Other / comments

140. Did you experience any persisting negative effects from this psychedelic experience? By persisting, we mean negative effects that lasted beyond the acute period of drug effects.

- ◯ Yes
- ◯ No
- ◯ Not sure

141. Please describe any negative or potentially negative persisting effects you may have experienced as a result of this psychedelic session.

142. Overall, how would you rate the severity of these negative effects?

- Not at all severe
- Slightly severe
- Moderately severe
- Very severe
- Extremely severe

The following questions pertain to your alcohol use in the year prior to your psychedelic experience. Your answers will remain confidential so please be honest. Select the options that best describe your answer to each question.

143. In the year prior to your psychedelic-associated alcohol use reduction, approximately how many alcoholic drinks did you consume per week?

144. In the year prior to your psychedelic-associated alcohol use reduction, how often did you have a drink containing alcohol?

- Never
- Monthly or less
- 2-4 times a month
- 2-3 times a week
- 4 or more times a week

145. In the year prior to your psychedelic-associated alcohol use reduction, how many drinks containing alcohol did you have on a typical day when you were drinking?

- 1 or 2
- 3 or 4
- 5 or 6
- 7 to 9
- 10 or more

146. During the year prior to your psychedelic-associated alcohol use reduction...

	Frequency
How often during the year prior to your psychedelic-associated alcohol use reduction did you have six or more drinks on one occasion?	
How often during the year prior to your psychedelic-associated alcohol use reduction did you find that you were not able to stop drinking once you had started?	
How often during the year prior to your psychedelic-associated alcohol use reduction did you fail to do what was normally expected of you because of drinking?	
How often during the year prior to your psychedelic-associated alcohol use reduction did you need a first drink in the morning to get yourself going after a heavy drinking session or to steady your nerves?	
How often during the year prior to your psychedelic-associated alcohol use reduction did you have a feeling of guilt or remorse after drinking?	
How often during the year prior to your psychedelic-associated alcohol use reduction were you unable to remember what happened the night before because of your drinking?	

147. During the year prior to your psychedelic-associated alcohol use reduction, were you or someone else injured because of your drinking?

- No
- Yes

148. During the year prior to your psychedelic-associated alcohol use reduction, was a relative, friend, doctor, or other health worker concerned about your drinking, or suggest that you should cut down?

- No
- Yes

149. In the year prior to your psychedelic-associated alcohol use reduction, please indicate whether each of the following was true or false regarding your alcohol use.

	True	False
In the year prior to my psychedelic experience, I often consumed alcohol in larger amounts or over a longer period than intended.	◯	◯
In the year prior to my psychedelic experience, I had a persistent desire or unsuccessful efforts to cut down or control alcohol use.	◯	◯
In the year prior to my psychedelic experience, I spent a great deal of time in activities necessary to obtain alcohol, use alcohol, or recover from its effects.	◯	◯
In the year prior to my psychedelic experience, I experienced craving, or a strong desire or urge to use alcohol.	◯	◯
In the year prior to my psychedelic experience, my recurrent alcohol use resulted in a failure to fulfill major role obligations at work, school, or home.	◯	◯
In the year prior to my psychedelic experience, I continued using alcohol despite having persistent or recurrent social or interpersonal problems caused or exacerbated by the effects of alcohol.	◯	◯
In the year prior to my psychedelic experience, I gave up or reduced important social, occupational, or recreational activities because of alcohol use.	◯	◯
In the year prior to my psychedelic experience, I engaged in recurrent alcohol use in situations in which it was physically hazardous.	◯	◯
In the year prior to my psychedelic experience, I continued using alcohol despite knowledge of having a persistent or recurrent physical or psychological problem that was likely to have been caused or exacerbated by alcohol.	◯	◯
In the year prior to my psychedelic experience, I experienced alcohol tolerance, as defined by either: A) a need for markedly increased amounts of alcohol to achieve intoxication or desired effect; or B) markedly diminished effect with continued use of the same amount of alcohol.	◯	◯
In the year prior to my psychedelic experience, I experienced alcohol withdrawal, as defined by either: A) the presence of these symptoms upon cessation of alcohol use: autonomic hyperactivity (e.g. sweating, high pulse), increased hand tremor, insomnia, nausea or vomiting, hallucinations or illusions, psychomotor agitation (e.g. pacing, wringing hands, etc.), anxiety, or seizures B) Alcohol was taken to relieve or avoid such withdrawal symptoms.	◯	◯

150. Listed below are questions that ask about your feelings about drinking <u>in the year prior to your psychedelic-associated alcohol use reduction.</u> The words "drinking" and "have a drink" refer to having a drink containing alcohol such as beer, wine, or liquor. Please indicate how much you would have agreed or disagreed with each of the following statements in the year before your psychedelic experience by selecting one response for each question between STRONGLY DISAGREE and STRONGLY AGREE. Please complete every item. <u>We are interested in how you were thinking or feeling generally during the year prior to your psychedelic-associated alcohol use reduction.</u>

	Response
All I would have wanted to do was have a drink.	
I would not have needed to have a drink.	
It would have been difficult to turn down a drink.	
Having a drink would have made things seem perfect.	
I would have wanted a drink so bad, I could almost taste it.	
Nothing would have tasted better than a drink.	
If I had the chance to have a drink, I don't think I would have drank it.	
I would have craved a drink.	

The following questions pertain to your alcohol use <u>in the time since your psychedelic experience.</u> Your answers will remain confidential so please be honest. Select the options that best describe your answer to each question.

151. In the period of time since your psychedelic-associated alcohol use reduction, approximately how many alcoholic drinks have you consumed per week?

152. In the period of time since your psychedelic-associated alcohol use reduction, how often did you have a drink containing alcohol?

- Never
- Monthly or less
- 2-4 times a month
- 2-3 times a week
- 4 or more times a week

153. In the period of time since your psychedelic-associated alcohol use reduction, how many drinks containing alcohol did you have on a typical day when you were drinking?

- 0, not applicable
- 1 or 2
- 3 or 4
- 5 or 6
- 7 to 9
- 10 or more

154. In the period of time since your psychedelic-associated alcohol use reduction...

	Frequency
How often in the period of time since your psychedelic experience did you have six or more drinks on one occasion?	
How often in the period of time since your psychedelic experience did you find that you were not able to stop drinking once you had started?	
How often in the period of time since your psychedelic experience did you fail to do what was normally expected of you because of drinking?	
How often in the period of time since your psychedelic experience did you need a first drink in the morning to get yourself going after a heavy drinking session or to steady your nerves?	
How often in the period of time since your psychedelic experience did you have a feeling of guilt or remorse after drinking?	
How often in the period of time since your psychedelic experience were you unable to remember what happened the night before because of your drinking?	

155. In the period of time since your psychedelic-associated alcohol use reduction, were you or someone else injured as a result of your drinking?

- No
- Yes

156. In the period of time since your psychedelic-associated alcohol use reduction, has a relative, friend, doctor, or other health worker been concerned about your drinking, or suggest that you should cut down?

- No
- Yes

157. In the time since your psychedelic-associated alcohol cessation or reduction, please indicate whether each of the following was true or false regarding your alcohol use.

	True	False
In the time since my psychedelic experience, I often consumed alcohol in larger amounts or over a longer period than intended.	◯	◯
In the time since my psychedelic experience, I had a persistent desire or unsuccessful efforts to cut down or control alcohol use.	◯	◯
In the time since my psychedelic experience, I spent a great deal of time in activities necessary to obtain alcohol, use alcohol, or recover from its effects.	◯	◯
In the time since my psychedelic experience, I experienced craving, or a strong desire or urge to use alcohol.	◯	◯
In the time since my psychedelic experience, my recurrent alcohol use resulted in a failure to fulfill major role obligations at work, school, or home.	◯	◯
In the time since my psychedelic experience, I continued using alcohol despite having persistent or recurrent social or interpersonal problems caused or exacerbated by the effects of alcohol.	◯	◯
In the time since my psychedelic experience, I gave up or reduced important social, occupational, or recreational activities because of alcohol use.	◯	◯
In the time since my psychedelic experience, I engaged in recurrent alcohol use in situations in which it is physically hazardous.	◯	◯
In the time since my psychedelic experience, I continued using alcohol despite knowledge of having a persistent or recurrent physical or psychological problem that is likely to have been caused or exacerbated by alcohol.	◯	◯
In the time since my psychedelic experience, I experienced alcohol tolerance, as defined by either: A) a need for markedly increased amounts of alcohol to achieve intoxication or desired effect; or B) markedly diminished effect with continued use of the same amount of alcohol.	◯	◯
In the time since my psychedelic experience, I experienced alcohol withdrawal, as defined by either: A) the presence of these symptoms upon cessation of alcohol use: autonomic hyperactivity (e.g. sweating, high pulse), increased hand tremor, insomnia, nausea or vomiting, hallucinations or illusions, psychomotor agitation (e.g. pacing, wringing hands, etc.), anxiety, or seizures B) Alcohol was taken to relieve or avoid such withdrawal symptoms.	◯	◯

158. Listed below are questions that ask about your feelings about drinking right now. The words "drinking" and "have a drink" refer to having a drink containing alcohol such as beer, wine, or liquor. Please indicate how much you agree or disagree with each of the following statements by selecting one response for each question between STRONGLY DISAGREE and STRONGLY AGREE. Please complete every item. We are interested in how you are thinking or feeling right now.

	Response
All I want to do now is have a drink.	
I do not need to have a drink right now.	
It would be difficult to turn down a drink this minute.	
Having a drink right now would make things seem perfect.	
I want a drink so bad I can almost taste it.	
Nothing would be better than a drink right now.	
If I had the chance to have a drink, I don't think I would drink it.	
I crave a drink right now.	

159. Have you had any alcoholic beverages...

	Yes	No
in the past 12 months?	◯	◯
in the past 6 months?	◯	◯
in the past month?	◯	◯
in the past week?	◯	◯
in the past 24 hours?	◯	◯

160. How old were you when you first started drinking alcohol?

161. How many years do you think you had a "drinking problem" before quitting or reducing your alcohol use?

Other (please specify)

162. Prior to your psychedelic experience, which of the following did you use in your efforts to quit or reduce drinking? (Check all that apply):

- ☐ None
- ☐ Detoxification at a treatment center
- ☐ Professional counseling or psychotherapy
- ☐ Counseling provided by a "Quit-line" telephone number DEL
- ☐ Counseling provided by a website (for example, WebMD)
- ☐ Hypnosis DEL
- ☐ Acupuncture DEL
- ☐ Alcoholics Anonymous (AA), or another support group DEL
- ☐ Self-help manuals or books DEL
- ☐ Disulfiram (Antabuse) DEL
- ☐ Naltrexone (Revia)
- ☐ Acamprosate (Campral)
- ☐ Spiritual / religious practice (Church attendance, meditation, etc.)

Other (please specify)

163. Prior to your psychedelic-associated alcohol use reduction, how many times in your life had you made a serious attempt to quit drinking (i.e., attempts lasting at least 1 day)?

164. Other than your psychedelic-associated alcohol use reduction, what was the longest period of time that you were able to refrain from drinking alcohol?

Other (please specify)

165. Some people report withdrawal symptoms (like the ones listed below) immediately upon stopping or reducing their regular alcohol use. After your psychedelic-associated alcohol use reduction, how serious were each of the following problems <u>in comparison to your previous quit attempts?</u>

	Not Applicable	Much Less Severe	Less Severe	Same	More Severe	Much More Severe
Lack of appetite	◯	◯	◯	◯	◯	◯
Tremors	◯	◯	◯	◯	◯	◯
Nausea and/or Vomiting	◯	◯	◯	◯	◯	◯
Tactile disturbances (itching, numbness, 'pins and needles' sensations)	◯	◯	◯	◯	◯	◯
Increased Heart rate	◯	◯	◯	◯	◯	◯
Headaches	◯	◯	◯	◯	◯	◯
Drowsiness	◯	◯	◯	◯	◯	◯
Depression or low mood	◯	◯	◯	◯	◯	◯
Confusion	◯	◯	◯	◯	◯	◯
Fatigue	◯	◯	◯	◯	◯	◯
Insomnia	◯	◯	◯	◯	◯	◯
Difficulty concentrating	◯	◯	◯	◯	◯	◯
Heart pounding, or sweating	◯	◯	◯	◯	◯	◯
Irritability	◯	◯	◯	◯	◯	◯
Restlessness	◯	◯	◯	◯	◯	◯
Hallucinations	◯	◯	◯	◯	◯	◯
Seizures	◯	◯	◯	◯	◯	◯
Anxiety	◯	◯	◯	◯	◯	◯
Fever	◯	◯	◯	◯	◯	◯
Alcohol craving	◯	◯	◯	◯	◯	◯

Comments

166. Would you say your quitting or reducing alcohol was the result of a single or multiple psychedelic sessions?

- ◯ Single Session
- ◯ Multiple Sessions

167. If your alcohol cessation or reduction was the result of multiple psychedelic sessions, please briefly describe the process, including substances ingested, number of sessions, and length of time between sessions.

For this section we would like you to focus on just one psychedelic experience that you deemed to be most important in affecting a decrease in your opioid use. If you reduced your opioid use after multiple psychedelic experiences, you will be able to provide more information on the substances, doses, and sequence of events in later sections.

168. How long ago did the psychedelic experience that led to your quitting or reducing opioids occur?

- Less than 1 week
- 1 - 2 weeks
- 3 - 4 weeks
- 1 - 3 months
- 4 - 6 months
- 7 - 12 months
- 1 - 2 years
- 3 - 5 years
- 6 - 10 years
- 11 - 20 years
- More than 20 years

169. Please characterize your opioid use after this psychedelic experience.

- Stopped using completely since the experience (Total abstinence from opioids).
- Greatly reduced using opioids since the experience.
- Reduced using opioids somewhat since the experience.
- Stopped using opioids completely for a period of time, then returned to using the same amount as before.
- Reduced using opioids for a period of time, then returned to using the same amount as before.

Other (please specify)

170. How long did your opioid reduction or abstinence last?

- ◯ Less than 1 week
- ◯ 1 - 2 weeks
- ◯ 3 - 4 weeks
- ◯ 1 - 3 months
- ◯ 4 - 6 months
- ◯ 7 - 12 months
- ◯ 1 - 2 years
- ◯ 3 - 5 years
- ◯ 6 - 10 years
- ◯ 11 - 20 years
- ◯ More than 20 years

171. Which substance led to the psychedelic experience associated with your quitting or reducing your opioid use?

- ◯ psilocybin mushrooms
- ◯ LSD
- ◯ morning glory seeds
- ◯ mescaline (pure compound)
- ◯ peyote cactus
- ◯ San Pedro cactus
- ◯ DMT (pure compound)
- ◯ Ayahuasca
- ◯ MDMA (ecstasy, Molly)

Other (please specify)

172. What was the approximate dose ingested to the best of your knowledge?

- ◯ Very low dose
- ◯ Low dose
- ◯ Moderate dose
- ◯ High dose
- ◯ Very high dose

173. How old were you when this experience took place?

174. Where did this experience take place? (Check all that apply):

- [ ] At home
- [ ] At a party
- [ ] In a public place (e.g., shopping mall, movie theater, etc.)
- [ ] At a concert or festival
- [ ] Outdoors in nature
- [ ] In a religious or spiritual setting (e.g., ceremony, retreat, etc.)

Other (please specify)

175. Were you alone or with other people during the majority of this psychedelic session?

- ◯ Alone
- ◯ With 1 other person
- ◯ With a few other people (2 - 5)
- ◯ With a small group of people (6 - 20)
- ◯ At a medium-sized gathering (21 - 100)
- ◯ At a large gathering (more than 100 people)

176. What was your intention in this particular instance for taking a psychedelic? (Check all that apply):

- [ ] I took a psychedelic only because other people were, but I did not have a serious intention.
- [ ] Curiosity without any other serious intention.
- [ ] Recreational (e.g., to enjoy the experience, which may or may not involve a social recreational event such as a concert).
- [ ] A serious intention for psychological self-exploration (e.g., resolve a personal issue or to increase self-understanding).
- [ ] A serious intention to explore spirituality or the sacred (e.g., to contemplate God, as you understand that word, or the nature of ultimate reality, and so on).
- [ ] A premeditated intention to quit or reduce your opioid use.
- [ ] Other (please specify)

177. After your psychedelic experience, did you use any other methods to help maintain opioid abstinence or reduction? (Check all that apply):

- [ ] None
- [ ] Detoxification at a treatment center
- [ ] Professional counseling or psychotherapy
- [ ] Counseling provided by a “Quit-line” telephone number DEL
- [ ] Counseling provided by a website (for example, WebMD)
- [ ] Hypnosis DEL
- [ ] Acupuncture DEL
- [ ] Narcotics Anonymous (NA), or another support group
- [ ] Self-help manuals or books DEL
- [ ] Methadone (Methadose) DEL
- [ ] Naltrexone (Revia)
- [ ] Buprenorphine (Suboxone)
- [ ] Spiritual / religious practice (Church attendance, meditation, etc.)
- [ ] Other (please specify)

178. Please provide a brief description of the psychedelic experience that led to your quitting or reducing your opioid use.

Looking back on the entirety of the psychedelic session that occurred prior to your opioid reduction or cessation, please rate the degree to which at any time during that session you experienced the following phenomena. Answer each question according to your feelings, thoughts, and experiences at the time of the psychedelic session.

179. Loss of your usual sense of time.

- 0 – none; not at all
- 1 – so slight cannot decide
- 2 – slight
- 3 – moderate
- 4 – strong (equivalent in degree to any other strong experience)
- 5 – extreme (more than any other time in my life and stronger than 4)

180. Experience of amazement.

- 0 – none; not at all
- 1 – so slight cannot decide
- 2 – slight
- 3 – moderate
- 4 – strong (equivalent in degree to any other strong experience)
- 5 – extreme (more than any other time in my life and stronger than 4)

181. Sense that the experience cannot be described adequately in words.

- 0 – none; not at all
- 1 – so slight cannot decide
- 2 – slight
- 3 – moderate
- 4 – strong (equivalent in degree to any other strong experience)
- 5 – extreme (more than any other time in my life and stronger than 4)

182. Gain of insightful knowledge experienced at an intuitive level.

- 0 – none; not at all
- 1 – so slight cannot decide
- 2 – slight
- 3 – moderate
- 4 – strong (equivalent in degree to any other strong experience)
- 5 – extreme (more than any other time in my life and stronger than 4)

183. Feeling that you experienced eternity or infinity.

- 0 – none; not at all
- 1 – so slight cannot decide
- 2 – slight
- 3 – moderate
- 4 – strong (equivalent in degree to any other strong experience)
- 5 – extreme (more than any other time in my life and stronger than 4)

184. Experience of oneness or unity with objects and/or persons perceived in your surroundings.

- 0 – none; not at all
- 1 – so slight cannot decide
- 2 – slight
- 3 – moderate
- 4 – strong (equivalent in degree to any other strong experience)
- 5 – extreme (more than any other time in my life and stronger than 4)

185. Loss of your usual sense of space.

- 0 – none; not at all
- 1 – so slight cannot decide
- 2 – slight
- 3 – moderate
- 4 – strong (equivalent in degree to any other strong experience)
- 5 – extreme (more than any other time in my life and stronger than 4)

186. Feelings of tenderness and gentleness.

- 0 – none; not at all
- 1 – so slight cannot decide
- 2 – slight
- 3 – moderate
- 4 – strong (equivalent in degree to any other strong experience)
- 5 – extreme (more than any other time in my life and stronger than 4)

187. Certainty of encounter with ultimate reality (in the sense of being able to "know" and "see" what is really real at some point during your experience).

- 0 – none; not at all
- 1 – so slight cannot decide
- 2 – slight
- 3 – moderate
- 4 – strong (equivalent in degree to any other strong experience)
- 5 – extreme (more than any other time in my life and stronger than 4)

188. Feeling that you could not do justice to your experience by describing it in words.

- 0 – none; not at all
- 1 – so slight cannot decide
- 2 – slight
- 3 – moderate
- 4 – strong (equivalent in degree to any other strong experience)
- 5 – extreme (more than any other time in my life and stronger than 4)

189. Loss of usual awareness of where you were.

- 0 – none; not at all
- 1 – so slight cannot decide
- 2 – slight
- 3 – moderate
- 4 – strong (equivalent in degree to any other strong experience)
- 5 – extreme (more than any other time in my life and stronger than 4)

190. Feelings of peace and tranquility.

- 0 – none; not at all
- 1 – so slight cannot decide
- 2 – slight
- 3 – moderate
- 4 – strong (equivalent in degree to any other strong experience)
- 5 – extreme (more than any other time in my life and stronger than 4)

191. Sense of being "outside of" time, beyond past and future.

- 0 – none; not at all
- 1 – so slight cannot decide
- 2 – slight
- 3 – moderate
- 4 – strong (equivalent in degree to any other strong experience)
- 5 – extreme (more than any other time in my life and stronger than 4)

192. Freedom from the limitations of your personal self and feeling a unity or bond with what was felt to be greater than your personal self.

- 0 – none; not at all
- 1 – so slight cannot decide
- 2 – slight
- 3 – moderate
- 4 – strong (equivalent in degree to any other strong experience)
- 5 – extreme (more than any other time in my life and stronger than 4)

193. Sense of being at a spiritual height.

- 0 – none; not at all
- 1 – so slight cannot decide
- 2 – slight
- 3 – moderate
- 4 – strong (equivalent in degree to any other strong experience)
- 5 – extreme (more than any other time in my life and stronger than 4)

194. Experience of pure being and pure awareness (beyond the world of sense impressions).

- 0 – none; not at all
- 1 – so slight cannot decide
- 2 – slight
- 3 – moderate
- 4 – strong (equivalent in degree to any other strong experience)
- 5 – extreme (more than any other time in my life and stronger than 4)

195. Experience of ecstasy.

- 0 – none; not at all
- 1 – so slight cannot decide
- 2 – slight
- 3 – moderate
- 4 – strong (equivalent in degree to any other strong experience)
- 5 – extreme (more than any other time in my life and stronger than 4)

196. Experience of the insight that “all is One”.

- 0 – none; not at all
- 1 – so slight cannot decide
- 2 – slight
- 3 – moderate
- 4 – strong (equivalent in degree to any other strong experience)
- 5 – extreme (more than any other time in my life and stronger than 4)

197. Being in a realm with no space boundaries.

- 0 – none; not at all
- 1 – so slight cannot decide
- 2 – slight
- 3 – moderate
- 4 – strong (equivalent in degree to any other strong experience)
- 5 – extreme (more than any other time in my life and stronger than 4)

198. Experience of oneness in relation to an "inner world" within.

- 0 – none; not at all
- 1 – so slight cannot decide
- 2 – slight
- 3 – moderate
- 4 – strong (equivalent in degree to any other strong experience)
- 5 – extreme (more than any other time in my life and stronger than 4)

199. Sense of reverence.

- 0 – none; not at all
- 1 – so slight cannot decide
- 2 – slight
- 3 – moderate
- 4 – strong (equivalent in degree to any other strong experience)
- 5 – extreme (more than any other time in my life and stronger than 4)

200. Experience of timelessness.

- 0 – none; not at all
- 1 – so slight cannot decide
- 2 – slight
- 3 – moderate
- 4 – strong (equivalent in degree to any other strong experience)
- 5 – extreme (more than any other time in my life and stronger than 4)

201. You are convinced now, as you look back on your experience, that in it you encountered ultimate reality (i.e., that you "knew" and "saw" what was really real).

- 0 – none; not at all
- 1 – so slight cannot decide
- 2 – slight
- 3 – moderate
- 4 – strong (equivalent in degree to any other strong experience)
- 5 – extreme (more than any other time in my life and stronger than 4)

202. Feeling that you experienced something profoundly sacred and holy.

- 0 – none; not at all
- 1 – so slight cannot decide
- 2 – slight
- 3 – moderate
- 4 – strong (equivalent in degree to any other strong experience)
- 5 – extreme (more than any other time in my life and stronger than 4)

203. Awareness of the life or living presence in all things.

- 0 – none; not at all
- 1 – so slight cannot decide
- 2 – slight
- 3 – moderate
- 4 – strong (equivalent in degree to any other strong experience)
- 5 – extreme (more than any other time in my life and stronger than 4)

204. Experience of the fusion of your personal self into a larger whole.

- 0 – none; not at all
- 1 – so slight cannot decide
- 2 – slight
- 3 – moderate
- 4 – strong (equivalent in degree to any other strong experience)
- 5 – extreme (more than any other time in my life and stronger than 4)

205. Sense of awe or awesomeness.

- 0 – none; not at all
- 1 – so slight cannot decide
- 2 – slight
- 3 – moderate
- 4 – strong (equivalent in degree to any other strong experience)
- 5 – extreme (more than any other time in my life and stronger than 4)

206. Experience of unity with ultimate reality.

- 0 – none; not at all
- 1 – so slight cannot decide
- 2 – slight
- 3 – moderate
- 4 – strong (equivalent in degree to any other strong experience)
- 5 – extreme (more than any other time in my life and stronger than 4)

207. Feeling that it would be difficult to communicate your own experience to others who have not had similar experiences.

- 0 – none; not at all
- 1 – so slight cannot decide
- 2 – slight
- 3 – moderate
- 4 – strong (equivalent in degree to any other strong experience)
- 5 – extreme (more than any other time in my life and stronger than 4)

208. Feelings of joy.

- 0 – none; not at all
- 1 – so slight cannot decide
- 2 – slight
- 3 – moderate
- 4 – strong (equivalent in degree to any other strong experience)
- 5 – extreme (more than any other time in my life and stronger than 4)

The purpose of the following questions is to obtain your retrospective assessment of the psychedelic experience that led to your opioid use reduction, <u>and your contemplation of that experience.</u> For all questions on this page, please choose the highest rating that applies.

209. How personally meaningful were the psychedelic experience that led to your opioid use reduction, and your contemplation of that experience?

- ◯ No more than routine, everyday experiences
- ◯ Similar to meaningful experiences that occur on average once or more a week
- ◯ Similar to meaningful experiences that occur on average once a month
- ◯ Similar to meaningful experiences that occur on average once a year
- ◯ Similar to meaningful experiences that occur on average once every 5 years
- ◯ Among the 10 most meaningful experiences of my life
- ◯ Among the 5 most meaningful experiences of my life
- ◯ The single most meaningful experience of my life

210. Indicate the degree to which the psychedelic experience that led to your opioid use reduction, and your contemplation of that experience, were spiritually significant to you.

- ◯ Not at all
- ◯ Slightly
- ◯ Moderately
- ◯ Very much
- ◯ Among the 5 most spiritually significant experiences of my life
- ◯ The single most spiritually significant experience of my life

211. How psychologically challenging was the most psychologically challenging portion of the psychedelic experience that led to your opioid use reduction?

- ◯ No more than routine, everyday experiences
- ◯ Similar to difficult or challenging experiences that occur on average once or more a week
- ◯ Similar to difficult or challenging experiences that occur on average once a month
- ◯ Similar to difficult or challenging experiences that occur on average once a year
- ◯ Similar to difficult or challenging experiences that occur on average once every 5 years
- ◯ Among the 10 most difficult or challenging experiences of my life
- ◯ Among the 5 most difficult or challenging experiences of my life
- ◯ The single most difficult or challenging experience of my life

212. How psychologically insightful to you were the psychedelic experience that led to your opioid use reduction, and your contemplation of that experience?

- ◯ No more than routine, everyday psychologically insightful experiences
- ◯ Similar to psychologically insightful experiences that occur on average once or more a week
- ◯ Similar to psychologically insightful experiences that occur on average once a month
- ◯ Similar to psychologically insightful experiences that occur on average once a year
- ◯ Similar to psychologically insightful experiences that occur on average once every 5 years
- ◯ Among the 10 most psychologically insightful experiences of my life
- ◯ Among the 5 most psychologically insightful experiences of my life
- ◯ The single most psychologically insightful experience of my life

213. Do you believe that the psychedelic experience that led to your opioid use reduction, and your contemplation of that experience, have led to change in your current sense of personal well-being or life satisfaction?

- ◯ Strong positive change that I consider desirable
- ◯ Moderate positive change that I consider desirable
- ◯ Slight positive change that I consider desirable
- ◯ No change
- ◯ Slight negative change that I consider undesirable
- ◯ Moderate negative change that I consider undesirable
- ◯ Strong negative change that I consider undesirable

214. Please rank items in order of their importance to your psychedelic-associated opioid abstinence or reduction.

Rank	Item	
☐	Strengthening your belief in your own ability to quit.	☐ Not applicable
☐	Reducing stress involved with quitting.	☐ Not applicable
☐	Reframing quitting as a spiritual task.	☐ Not applicable
☐	Changing life priorities or values, such that using was no longer more important than quitting.	☐ Not applicable
☐	Changing your orientation toward the future, so that long-term benefits outweighed immediate desires.	☐ Not applicable
☐	Increasing space between the experience of craving and taking action.	☐ Not applicable

215. Please describe any additional reasons (not listed above) that your psychedelic experience may have contributed to your quitting or reducing your opioid use.

216. Did you experience any other behavioral changes after this psychedelic session? Check all that apply, and please provide any relevant details in the Comments box below.

- [ ] None
- [ ] Reduced or stopped using other drugs
- [ ] Started using other drugs more often / heavily
- [ ] Improved diet / nutrition
- [ ] Worsened diet / nutrition
- [ ] Increased physical activity / exercise
- [ ] Decreased physical activity / exercise
- [ ] Improved relationships with others
- [ ] Worsened relationships with others
- [ ] Improvements in career / work life
- [ ] Worsening of career / work life

Other / comments

217. Did you experience any persisting negative effects from this psychedelic experience? By persisting, we mean negative effects that lasted beyond the acute period of drug effects.

- ○ Yes
- ○ No
- ○ Not sure

218. Please describe any negative or potentially negative persisting effects you may have experienced as a result of this psychedelic session.

219. Overall, how would you rate the severity of these negative effects?

- Not at all severe
- Slightly severe
- Moderately severe
- Very severe
- Extremely severe

The following questions pertain to your opioid use <u>in the year prior to your psychedelic experience.</u> Your answers will remain confidential so please be honest. Select the options that best describe your answer to each question.

220. What was your opioid of choice and preferred route of administration?

	Drug	Route of Administration
Please choose only one from each menu:		

Other (please specify)

221. In the year prior to your psychedelic-associated opioid use reduction, how often did you use opioids?

- Never
- Monthly or less
- 2-4 times a month
- 2-3 times a week
- 4 or more times a week

222. In the year prior to your psychedelic-associated opioid use reduction, how many times would you take opioids on a typical day when you were using?

- 1 or 2
- 3 or 4
- 5 or 6
- 7 to 9
- 10 or more

223. During the year prior to your psychedelic-associated opioid use reduction...

	Frequency
How often in the year prior to your psychedelic experience did you use a noticeable psychoactive dose of opioids?	
How often in the year prior to your psychedelic experience did you find that you used more opioids than you thought you would?	
How often during the year prior to your psychedelic experience did you fail to do what was normally expected of you because of your opioid use?	
How often during the year prior to your psychedelic experience did you need to use first thing in the morning to get yourself going or to steady your nerves?	
How often during the year prior to your psychedelic experience did you have a feeling of guilt or remorse after using opioids?	
How often during the year prior to your psychedelic experience, were you unable to remember what happened the night before because of your opioid use?	

224. During the year prior to your psychedelic-associated opioid use reduction, were you or someone else injured because of your opioid use?

- No
- Yes

225. During the year prior to your psychedelic-associated opioid use reduction, was a relative, friend, doctor, or other health worker concerned about your opioid use, or suggest that you should cut down?

- ◯ No
- ◯ Yes

226. In the year prior to your psychedelic-associated opioid use reduction, please indicate whether each of the following was true or false regarding your opioid use.

	True	False
In the year prior to my psychedelic experience, I often used larger amounts of opioids or used over a longer period than intended.	◯	◯
In the year prior to my psychedelic experience, I had a persistent desire or unsuccessful efforts to cut down or control opioid use.	◯	◯
In the year prior to my psychedelic experience, I spent a great deal of time in activities necessary to obtain opioids, use opioids, or recover from their effects.	◯	◯
In the year prior to my psychedelic experience, I experienced cravings, or a strong desire or urge to use opioids.	◯	◯
In the year prior to my psychedelic experience, my recurrent opioid use resulted in a failure to fulfill major role obligations at work, school, or home.	◯	◯
In the year prior to my psychedelic experience, I continued using opioids despite having persistent or recurrent social or interpersonal problems caused or exacerbated by the effects of opioids.	◯	◯
In the year prior to my psychedelic experience, I gave up or reduced important social, occupational, or recreational activities because of opioid use.	◯	◯
In the year prior to my psychedelic experience, I engaged in recurrent opioid use in situations in which it was physically hazardous.	◯	◯
In the year prior to my psychedelic experience, I continued using opioids despite knowledge of having a persistent or recurrent physical or psychological problem that was likely to have been caused or exacerbated by opioids.	◯	◯
In the year prior to my psychedelic experience, I experienced opioid tolerance, as defined by either: A) a need for markedly increased amounts of opioids to achieve intoxication or desired effect; or B) markedly diminished effect with continued use of the same amount of opioids.	◯	◯
In the year prior to my psychedelic experience, I experienced opioid withdrawal, as defined by either: A) the presence of these symptoms upon cessation of opioid use: depression, anxiety, nausea or vomiting, muscle aches, lacrimation(watery eyes) or rhinorrhea (runny nose), pupil dilation, sweating, diarrhea, yawning, fever, or insomnia; or B) Opioids were taken to relieve or avoid such withdrawal symptoms.	◯	◯

227. Listed below are questions that ask about your feelings about using opioids <u>in the year prior to your psychedelic-associated opioid use reduction.</u> The word "using" refers to taking opioids in order to get high or to "stay regular." Please indicate how much you would have agreed or disagreed with each of the following statements in the year before your psychedelic experience by selecting one response for each question between STRONGLY DISAGREE and STRONGLY AGREE. Please complete every item. <u>We are interested in how you were thinking or feeling generally during the year prior to your psychedelic-associated opioid use reduction.</u>

	Response
All I would have wanted to do was to use opioids.	
I would not have needed to use opioids.	
It would have been difficult to turn down opioids.	
Using opioids would have made things seem perfect.	
I would have wanted to use opioids so bad, I could almost taste it.	
Nothing would have felt better than using opioids.	
If I had the chance to use opioids, I don't think I would have.	
I would have craved opioids.	

The following questions pertain to your opioid use <u>in the time since your psychedelic experience.</u> Your answers will remain confidential so please be honest. Select the options that best describe your answer to each question.

228. In the period of time since your psychedelic-associated opioid use reduction, how often did you use opioids?

- ◯ Never
- ◯ Monthly or less
- ◯ 2-4 times a month
- ◯ 2-3 times a week
- ◯ 4 or more times a week

229. In the period of time since your psychedelic-associated opioid use reduction, how many times did you take opioids on a typical day when you were using?

- ◯ 0, not applicable
- ◯ 1 or 2
- ◯ 3 or 4
- ◯ 5 or 6
- ◯ 7 to 9
- ◯ 10 or more

230. In the period of time since your psychedelic-associated opioid use reduction...

	Frequency
How often in the period of time since your psychedelic experience did you use a noticeable psychoactive dose of opioids?	
How often in the period of time since your psychedelic experience did you find that you were not able to stop using opioids once you had started?	
How often in the period of time since your psychedelic experience did you fail to do what was normally expected of you because of your opioid use?	
How often in the period of time since your psychedelic experience did you need to use opioids in the morning to get yourself going or to steady your nerves?	
How often in the period of time since your psychedelic experience did you have a feeling of guilt or remorse after using opioids?	
How often in the period of time since your psychedelic experience were you unable to remember what happened the night before because of your opioid use?	

231. In the period of time since your psychedelic-associated opioid use reduction, were you or someone else injured as a result of your opioid use?

- ◯ No
- ◯ Yes

232. In the period of time since your psychedelic-associated opioid use reduction, has a relative, friend, doctor, or other health worker been concerned about your opioid use, or suggest that you should cut down?

- ◯ No
- ◯ Yes

233. In the time since your psychedelic-associated opioid cessation or reduction, please indicate whether each of the following was true or false regarding your opioid use.

	True	False
In the time since my psychedelic experience, I often took opioids in larger amounts or over a longer period than was intended.	◯	◯
In the time since my psychedelic experience, I had a persistent desire or unsuccessful efforts to cut down or control my opioid use.	◯	◯
In the time since my psychedelic experience, I spent a great deal of time in activities necessary to obtain opioids, use opioids, or recover from their effects.	◯	◯
In the time since my psychedelic experience, I experienced cravings, or a strong desire or urge to use opioids.	◯	◯
In the time since my psychedelic experience, my recurrent opioid use resulted in a failure to fulfill major role obligations at work, school, or home.	◯	◯
In the time since my psychedelic experience, I continued using opioids despite having persistent or recurrent social or interpersonal problems caused or exacerbated by the effects of opioids.	◯	◯
In the time since my psychedelic experience, I gave up or reduced important social, occupational, or recreational activities because of opioid use.	◯	◯
In the time since my psychedelic experience, I engaged in recurrent opioid use in situations in which it was physically hazardous.	◯	◯
In the time since my psychedelic experience, I continued using opioids despite knowledge of having a persistent or recurrent physical or psychological problem that was likely to have been caused or exacerbated by opioids.	◯	◯
In the time since my psychedelic experience, I experienced opioid tolerance, as defined by either: A) a need for markedly increased amounts of opioids to achieve intoxication or desired effect; or B) markedly diminished effect with continued use of the same amount of opioids.	◯	◯
In the time since my psychedelic experience, I experienced opioid withdrawal, as defined by either: A) the presence of these symptoms upon cessation of opioid use: depression, anxiety, nausea or vomiting, muscle aches, lacrimation (watery eyes) or rhinorrhea (runny nose), pupil dilation, sweating, diarrhea, yawning, fever, or insomnia; or B) Opioids were taken to relieve or avoid such withdrawal symptoms	◯	◯

234. Listed below are questions that ask about your feelings about using opioids right now. The word "using" refers to taking an opioid to get high or "stay regular." Please indicate how much you agree or disagree with each of the following statements by selecting one response for each question between STRONGLY DISAGREE and STRONGLY AGREE. Please complete every item. We are interested in how you are thinking or feeling right now.

	Response
All I want to do now is use opioids.	
I do not need to use opioids right now.	
It would be difficult to turn down opioids this minute.	
Using opioids right now would make things seem perfect.	
I want to use opioids so bad I can almost taste it.	
Nothing would be better than using opioids right now.	
If I had the chance to use opioids now, I don't think I would.	
I crave opioids right now.	

235. Have you used any opioids...

	Yes	No
in the past 12 months?	○	○
in the past 6 months?	○	○
in the past month?	○	○
in the past week?	○	○
in the past 24 hours?	○	○

236. How old were you when you first started using opioids?

237. How many years do you think you had an "opioid problem" before quitting or reducing your opioid use?

Other (please specify)

238. Prior to your psychedelic experience, which of the following did you use in your efforts to quit or reduce opioid use? (Check all that apply):

- [ ] None
- [ ] Detoxification at a treatment center
- [ ] Professional counseling or psychotherapy
- [ ] Counseling provided by a "Quit-line" telephone number DEL
- [ ] Counseling provided by a website (for example, WebMD)
- [ ] Hypnosis DEL
- [ ] Acupuncture DEL
- [ ] Narcotics Anonymous (NA), or another support group
- [ ] Self-help manuals or books DEL
- [ ] Methadone (Methadose) DEL
- [ ] Naltrexone (Revia)
- [ ] Buprenorphine (Suboxone)
- [ ] Spiritual / religious practice (Church attendance, meditation, etc.)

Other (please specify)

239. Prior to your psychedelic-associated opioid use reduction, how many times in your life had you made a serious attempt to quit using (i.e., attempts lasting at least 1 day)?

240. Other than your psychedelic-associated opioid use reduction, what was the longest period of time that you were able to refrain from using opioids?

Other (please specify)

241. Some people report withdrawal symptoms (like the ones listed below) immediately upon stopping or reducing their regular opioid use. After your psychedelic-associated opioid use reduction, how serious were each of the following problems <u>in comparison to your previous quit attempts?</u>

	Not Applicable	Much Less Severe	Less Severe	Same	More Severe	Much More Severe
Lack of appetite	○	○	○	○	○	○
Tremors	○	○	○	○	○	○
Nausea and/or Vomiting	○	○	○	○	○	○
Diarrhea	○	○	○	○	○	○
Rhinorrhea (runny nose)	○	○	○	○	○	○
Increased Heart rate	○	○	○	○	○	○
Headaches	○	○	○	○	○	○
Drowsiness or Yawning	○	○	○	○	○	○
Depression or low mood	○	○	○	○	○	○
Lacrimation (watery eyes)	○	○	○	○	○	○
Fatigue	○	○	○	○	○	○
Insomnia	○	○	○	○	○	○
Difficulty concentrating	○	○	○	○	○	○
Muscle aches	○	○	○	○	○	○
Heart pounding, or sweating	○	○	○	○	○	○
Chills	○	○	○	○	○	○
Irritability	○	○	○	○	○	○
Restlessness	○	○	○	○	○	○
Seizures	○	○	○	○	○	○
Anxiety	○	○	○	○	○	○
Fever	○	○	○	○	○	○
Opioid craving	○	○	○	○	○	○

Comments

242. Would you say your quitting or reducing your opioid use was the result of a single or multiple psychedelic sessions?

- ◯ Single Session
- ◯ Multiple Sessions

243. If your opioid cessation or reduction was the result of multiple psychedelic sessions, please briefly describe the process, including substances ingested, number of sessions, and length of time between sessions.

For this section we would like you to <u>focus on just one psychedelic experience that you deemed to be most important in affecting a decrease in your stimulant use.</u> If you reduced your stimulant use after multiple psychedelic experiences, you will be able to provide more information on the substances, doses, and sequence of events in later sections.

244. What was your stimulant of choice and preferred route of administration?

	Drug	Route of Administration
Please choose only one from each menu:		

Other (please specify)

245. How long ago did the psychedelic experience that led to your quitting or reducing stimulants occur?

- Less than 1 week
- 1 - 2 weeks
- 3 - 4 weeks
- 1 - 3 months
- 4 - 6 months
- 7 - 12 months
- 1 - 2 years
- 3 - 5 years
- 6 - 10 years
- 11 - 20 years
- More than 20 years

246. Please characterize your stimulant use after this psychedelic experience.

- Stopped using completely since the experience (Total abstinence from stimulants).
- Greatly reduced using stimulants since the experience.
- Reduced using stimulants somewhat since the experience.
- Stopped using stimulants completely for a period of time, then returned to using the same amount as before.
- Reduced using stimulants for a period of time, then returned to using the same amount as before.

Other (please specify)

247. How long did your stimulant reduction or abstinence last?

- ◯ Less than 1 week
- ◯ 1 - 2 weeks
- ◯ 3 - 4 weeks
- ◯ 1 - 3 months
- ◯ 4 - 6 months
- ◯ 7 - 12 months
- ◯ 1 - 2 years
- ◯ 3 - 5 years
- ◯ 6 - 10 years
- ◯ 11 - 20 years
- ◯ More than 20 years

248. Which substance led to the psychedelic experience associated with your quitting or reducing your stimulant use?

- ◯ psilocybin mushrooms
- ◯ LSD
- ◯ morning glory seeds
- ◯ mescaline (pure compound)
- ◯ peyote cactus
- ◯ San Pedro cactus
- ◯ DMT (pure compound)
- ◯ Ayahuasca
- ◯ MDMA (ecstasy, Molly)

Other (please specify)

249. What was the approximate dose ingested to the best of your knowledge?

- ◯ Very low dose
- ◯ Low dose
- ◯ Moderate dose
- ◯ High dose
- ◯ Very high dose

250. How old were you when this experience took place?

251. Where did this experience take place? (Check all that apply):

- [ ] At home
- [ ] At a party
- [ ] In a public place (e.g., shopping mall, movie theater, etc.)
- [ ] At a concert or festival
- [ ] Outdoors in nature
- [ ] In a religious or spiritual setting (e.g., ceremony, retreat, etc.)

Other (please specify)

252. Were you alone or with other people during the majority of this psychedelic session?

- ○ Alone
- ○ With 1 other person
- ○ With a few other people (2 - 5)
- ○ With a small group of people (6 - 20)
- ○ At a medium-sized gathering (21 - 100)
- ○ At a large gathering (more than 100 people)

253. What was your intention in this particular instance for taking a psychedelic? (Check all that apply):

- [ ] I took a psychedelic only because other people were, but I did not have a serious intention.
- [ ] Curiosity without any other serious intention.
- [ ] Recreational (e.g., to enjoy the experience, which may or may not involve a social recreational event such as a concert).
- [ ] A serious intention for psychological self-exploration (e.g., resolve a personal issue or to increase self-understanding).
- [ ] A serious intention to explore spirituality or the sacred (e.g., to contemplate God, as you understand that word, or the nature of ultimate reality, and so on).
- [ ] A premeditated intention to quit or reduce your stimulant use.
- [ ] Other (please specify)

254. After your psychedelic experience, did you use any other methods to help maintain stimulant abstinence or reduction? (Check all that apply):

- [ ] None
- [ ] Detoxification at a treatment center
- [ ] Professional counseling or psychotherapy
- [ ] Counseling provided by a "Quit-line" telephone number DEL
- [ ] Counseling provided by a website (for example, WebMD)
- [ ] Hypnosis DEL
- [ ] Acupuncture DEL
- [ ] Narcotics Anonymous (NA), or another support group
- [ ] Self-help manuals or books
- [ ] Prescribed medication (please specify in comments section) DEL
- [ ] Spiritual / religious practice (Church attendance, meditation, etc.)
- [ ] Other / comments (please specify)

255. Please provide a brief description of the psychedelic experience that led to your quitting or reducing your stimulant use.

Looking back on the entirety of the psychedelic session that occurred prior to your stimulant reduction or cessation, please rate the degree to which at any time during that session you experienced the following phenomena. Answer each question according to your feelings, thoughts, and experiences at the time of the psychedelic session.

256. Loss of your usual sense of time.

- 0 – none; not at all
- 1 – so slight cannot decide
- 2 – slight
- 3 – moderate
- 4 – strong (equivalent in degree to any other strong experience)
- 5 – extreme (more than any other time in my life and stronger than 4)

257. Experience of amazement.

- 0 – none; not at all
- 1 – so slight cannot decide
- 2 – slight
- 3 – moderate
- 4 – strong (equivalent in degree to any other strong experience)
- 5 – extreme (more than any other time in my life and stronger than 4)

258. Sense that the experience cannot be described adequately in words.

- 0 – none; not at all
- 1 – so slight cannot decide
- 2 – slight
- 3 – moderate
- 4 – strong (equivalent in degree to any other strong experience)
- 5 – extreme (more than any other time in my life and stronger than 4)

259. Gain of insightful knowledge experienced at an intuitive level.

- 0 – none; not at all
- 1 – so slight cannot decide
- 2 – slight
- 3 – moderate
- 4 – strong (equivalent in degree to any other strong experience)
- 5 – extreme (more than any other time in my life and stronger than 4)

260. Feeling that you experienced eternity or infinity.

- 0 – none; not at all
- 1 – so slight cannot decide
- 2 – slight
- 3 – moderate
- 4 – strong (equivalent in degree to any other strong experience)
- 5 – extreme (more than any other time in my life and stronger than 4)

261. Experience of oneness or unity with objects and/or persons perceived in your surroundings.

- 0 – none; not at all
- 1 – so slight cannot decide
- 2 – slight
- 3 – moderate
- 4 – strong (equivalent in degree to any other strong experience)
- 5 – extreme (more than any other time in my life and stronger than 4)

262. Loss of your usual sense of space.

- 0 – none; not at all
- 1 – so slight cannot decide
- 2 – slight
- 3 – moderate
- 4 – strong (equivalent in degree to any other strong experience)
- 5 – extreme (more than any other time in my life and stronger than 4)

263. Feelings of tenderness and gentleness.

- 0 – none; not at all
- 1 – so slight cannot decide
- 2 – slight
- 3 – moderate
- 4 – strong (equivalent in degree to any other strong experience)
- 5 – extreme (more than any other time in my life and stronger than 4)

264. Certainty of encounter with ultimate reality (in the sense of being able to "know" and "see" what is really real at some point during your experience).

- 0 – none; not at all
- 1 – so slight cannot decide
- 2 – slight
- 3 – moderate
- 4 – strong (equivalent in degree to any other strong experience)
- 5 – extreme (more than any other time in my life and stronger than 4)

265. Feeling that you could not do justice to your experience by describing it in words.

- 0 – none; not at all
- 1 – so slight cannot decide
- 2 – slight
- 3 – moderate
- 4 – strong (equivalent in degree to any other strong experience)
- 5 – extreme (more than any other time in my life and stronger than 4)

266. Loss of usual awareness of where you were.

- 0 – none; not at all
- 1 – so slight cannot decide
- 2 – slight
- 3 – moderate
- 4 – strong (equivalent in degree to any other strong experience)
- 5 – extreme (more than any other time in my life and stronger than 4)

267. Feelings of peace and tranquility.

- 0 – none; not at all
- 1 – so slight cannot decide
- 2 – slight
- 3 – moderate
- 4 – strong (equivalent in degree to any other strong experience)
- 5 – extreme (more than any other time in my life and stronger than 4)

268. Sense of being "outside of" time, beyond past and future.

- 0 – none; not at all
- 1 – so slight cannot decide
- 2 – slight
- 3 – moderate
- 4 – strong (equivalent in degree to any other strong experience)
- 5 – extreme (more than any other time in my life and stronger than 4)

269. Freedom from the limitations of your personal self and feeling a unity or bond with what was felt to be greater than your personal self.

- 0 – none; not at all
- 1 – so slight cannot decide
- 2 – slight
- 3 – moderate
- 4 – strong (equivalent in degree to any other strong experience)
- 5 – extreme (more than any other time in my life and stronger than 4)

270. Sense of being at a spiritual height.

- 0 – none; not at all
- 1 – so slight cannot decide
- 2 – slight
- 3 – moderate
- 4 – strong (equivalent in degree to any other strong experience)
- 5 – extreme (more than any other time in my life and stronger than 4)

271. Experience of pure being and pure awareness (beyond the world of sense impressions).

- 0 – none; not at all
- 1 – so slight cannot decide
- 2 – slight
- 3 – moderate
- 4 – strong (equivalent in degree to any other strong experience)
- 5 – extreme (more than any other time in my life and stronger than 4)

272. Experience of ecstasy.

- 0 – none; not at all
- 1 – so slight cannot decide
- 2 – slight
- 3 – moderate
- 4 – strong (equivalent in degree to any other strong experience)
- 5 – extreme (more than any other time in my life and stronger than 4)

273. Experience of the insight that “all is One”.

- 0 – none; not at all
- 1 – so slight cannot decide
- 2 – slight
- 3 – moderate
- 4 – strong (equivalent in degree to any other strong experience)
- 5 – extreme (more than any other time in my life and stronger than 4)

274. Being in a realm with no space boundaries.

- 0 – none; not at all
- 1 – so slight cannot decide
- 2 – slight
- 3 – moderate
- 4 – strong (equivalent in degree to any other strong experience)
- 5 – extreme (more than any other time in my life and stronger than 4)

275. Experience of oneness in relation to an “inner world” within.

- 0 – none; not at all
- 1 – so slight cannot decide
- 2 – slight
- 3 – moderate
- 4 – strong (equivalent in degree to any other strong experience)
- 5 – extreme (more than any other time in my life and stronger than 4)

276. Sense of reverence.

- 0 – none; not at all
- 1 – so slight cannot decide
- 2 – slight
- 3 – moderate
- 4 – strong (equivalent in degree to any other strong experience)
- 5 – extreme (more than any other time in my life and stronger than 4)

277. Experience of timelessness.

- 0 – none; not at all
- 1 – so slight cannot decide
- 2 – slight
- 3 – moderate
- 4 – strong (equivalent in degree to any other strong experience)
- 5 – extreme (more than any other time in my life and stronger than 4)

278. You are convinced now, as you look back on your experience, that in it you encountered ultimate reality (i.e., that you "knew" and "saw" what was really real).

- 0 – none; not at all
- 1 – so slight cannot decide
- 2 – slight
- 3 – moderate
- 4 – strong (equivalent in degree to any other strong experience)
- 5 – extreme (more than any other time in my life and stronger than 4)

279. Feeling that you experienced something profoundly sacred and holy.

- 0 – none; not at all
- 1 – so slight cannot decide
- 2 – slight
- 3 – moderate
- 4 – strong (equivalent in degree to any other strong experience)
- 5 – extreme (more than any other time in my life and stronger than 4)

280. Awareness of the life or living presence in all things.

- 0 – none; not at all
- 1 – so slight cannot decide
- 2 – slight
- 3 – moderate
- 4 – strong (equivalent in degree to any other strong experience)
- 5 – extreme (more than any other time in my life and stronger than 4)

281. Experience of the fusion of your personal self into a larger whole.

- 0 – none; not at all
- 1 – so slight cannot decide
- 2 – slight
- 3 – moderate
- 4 – strong (equivalent in degree to any other strong experience)
- 5 – extreme (more than any other time in my life and stronger than 4)

282. Sense of awe or awesomeness.

- 0 – none; not at all
- 1 – so slight cannot decide
- 2 – slight
- 3 – moderate
- 4 – strong (equivalent in degree to any other strong experience)
- 5 – extreme (more than any other time in my life and stronger than 4)

283. Experience of unity with ultimate reality.

- 0 – none; not at all
- 1 – so slight cannot decide
- 2 – slight
- 3 – moderate
- 4 – strong (equivalent in degree to any other strong experience)
- 5 – extreme (more than any other time in my life and stronger than 4)

284. Feeling that it would be difficult to communicate your own experience to others who have not had similar experiences.

- 0 – none; not at all
- 1 – so slight cannot decide
- 2 – slight
- 3 – moderate
- 4 – strong (equivalent in degree to any other strong experience)
- 5 – extreme (more than any other time in my life and stronger than 4)

285. Feelings of joy.

- 0 – none; not at all
- 1 – so slight cannot decide
- 2 – slight
- 3 – moderate
- 4 – strong (equivalent in degree to any other strong experience)
- 5 – extreme (more than any other time in my life and stronger than 4)

The purpose of the following questions is to obtain your retrospective assessment of the psychedelic experience that led to your stimulant use reduction, <u>and your contemplation of that experience.</u> For all questions on this page, please choose the highest rating that applies.

286. How personally meaningful were the psychedelic experience that led to your stimulant use reduction, and your contemplation of that experience?

- No more than routine, everyday experiences
- Similar to meaningful experiences that occur on average once or more a week
- Similar to meaningful experiences that occur on average once a month
- Similar to meaningful experiences that occur on average once a year
- Similar to meaningful experiences that occur on average once every 5 years
- Among the 10 most meaningful experiences of my life
- Among the 5 most meaningful experiences of my life
- The single most meaningful experience of my life

287. Indicate the degree to which the psychedelic experience that led to your stimulant use reduction, and your contemplation of that experience, were spiritually significant to you.

- Not at all
- Slightly
- Moderately
- Very much
- Among the 5 most spiritually significant experiences of my life
- The single most spiritually significant experience of my life

288. How psychologically challenging was the most psychologically challenging portion of the psychedelic experience that led to your stimulant use reduction?

- No more than routine, everyday experiences
- Similar to difficult or challenging experiences that occur on average once or more a week
- Similar to difficult or challenging experiences that occur on average once a month
- Similar to difficult or challenging experiences that occur on average once a year
- Similar to difficult or challenging experiences that occur on average once every 5 years
- Among the 10 most difficult or challenging experiences of my life
- Among the 5 most difficult or challenging experiences of my life
- The single most difficult or challenging experience of my life

289. How psychologically insightful to you were the psychedelic experience that led to your stimulant use reduction, and your contemplation of that experience?

- No more than routine, everyday psychologically insightful experiences
- Similar to psychologically insightful experiences that occur on average once or more a week
- Similar to psychologically insightful experiences that occur on average once a month
- Similar to psychologically insightful experiences that occur on average once a year
- Similar to psychologically insightful experiences that occur on average once every 5 years
- Among the 10 most psychologically insightful experiences of my life
- Among the 5 most psychologically insightful experiences of my life
- The single most psychologically insightful experience of my life

290. Do you believe that the psychedelic experience that led to your stimulant use reduction, and your contemplation of that experience, have led to change in your current sense of personal well-being or life satisfaction?

- Strong positive change that I consider desirable
- Moderate positive change that I consider desirable
- Slight positive change that I consider desirable
- No change
- Slight negative change that I consider undesirable
- Moderate negative change that I consider undesirable
- Strong negative change that I consider undesirable

291. Please rank items in order of their importance to your psychedelic-associated stimulant abstinence or reduction.

[ ]	Strengthening your belief in your own ability to quit.	Not applicable
[ ]	Reducing stress involved with quitting.	Not applicable
[ ]	Reframing quitting as a spiritual task.	Not applicable
[ ]	Changing life priorities or values, such that using was no longer more important than quitting.	Not applicable
[ ]	Changing your orientation toward the future, so that long-term benefits outweighed immediate desires.	Not applicable
[ ]	Increasing space between the experience of craving and taking action.	Not applicable

292. Please describe any additional reasons (not listed above) that your psychedelic experience may have contributed to your quitting or reducing your stimulant use.

293. Did you experience any other behavioral changes after this psychedelic session? Check all that apply, and please provide any relevant details in the Comments box below.

- ☐ None
- ☐ Reduced or stopped using other drugs
- ☐ Started using other drugs more often / heavily
- ☐ Improved diet / nutrition
- ☐ Worsened diet / nutrition
- ☐ Increased physical activity / exercise
- ☐ Decreased physical activity / exercise
- ☐ Improved relationships with others
- ☐ Worsened relationships with others
- ☐ Improvements in career / work life
- ☐ Worsening of career / work life

Other / comments

294. Did you experience any persisting negative effects from this psychedelic experience? By persisting, we mean negative effects that lasted beyond the acute period of drug effects.

- ◯ Yes
- ◯ No
- ◯ Not sure

295. Please describe any negative or potentially negative persisting effects you may have experienced as a result of this psychedelic session.

296. Overall, how would you rate the severity of these negative effects?

- ◯ Not at all severe
- ◯ Slightly severe
- ◯ Moderately severe
- ◯ Very severe
- ◯ Extremely severe

The following questions pertain to your stimulant use <u>in the year prior to your psychedelic experience.</u> Your answers will remain confidential so please be honest. Select the options that best describe your answer to each question.

297. In the year prior to your psychedelic-associated stimulant use reduction, how often did you use stimulants?

- Never
- Monthly or less
- 2-4 times a month
- 2-3 times a week
- 4 or more times a week

298. In the year prior to your psychedelic-associated stimulant use reduction, how many times would you take stimulants on a typical day when you were using?

- 1 or 2
- 3 or 4
- 5 or 6
- 7 to 9
- 10 or more

299. During the year prior to your psychedelic-associated stimulant use reduction...

	Frequency
How often during the year prior to your psychedelic experience did you use a noticeable psychoactive dose of a stimulant drug?	
How often during the year prior to your psychedelic experience did you find that you used more stimulants than you thought you would?	
How often during the year prior to your psychedelic experience did you fail to do what was normally expected of you because of your stimulant use?	
How often during the year prior to your psychedelic experience did you need to use a stimulant first thing in the morning to get yourself going or to steady your nerves?	
How often during the year prior to your psychedelic experience did you have a feeling of guilt or remorse after using stimulants?	
How often during the year prior to your psychedelic experience were you unable to remember what happened the night before because of your stimulant use?	

300. During the year prior to your psychedelic-associated stimulant use reduction, were you or someone else injured as a result of your stimulant use?

- No
- Yes

301. During the year prior to your psychedelic-associated stimulant use reduction, was a relative, friend, doctor, or other health worker concerned about your stimulant use, or suggest that you should cut down?

- ◯ No
- ◯ Yes

302. In the year prior to your psychedelic-associated stimulant cessation or reduction, please indicate whether each of the following was true or false regarding your stimulant use.

	True	False
In the year prior to my psychedelic experience, I often used larger amounts of stimulants or used over a longer period than intended.	◯	◯
In the year prior to my psychedelic experience, I had a persistent desire or unsuccessful efforts to cut down or control my stimulant use.	◯	◯
In the year prior to my psychedelic experience, I spent a great deal of time in activities necessary to obtain stimulants, use stimulants, or recover from their effects.	◯	◯
In the year prior to my psychedelic experience, I experienced cravings, or a strong desire or urge to use stimulants.	◯	◯
In the year prior to my psychedelic experience, my recurrent stimulant use resulted in a failure to fulfill major role obligations at work, school, or home.	◯	◯
In the year prior to my psychedelic experience, I continued using stimulants despite having persistent or recurrent social or interpersonal problems caused or exacerbated by the effects of stimulants.	◯	◯
In the year prior to my psychedelic experience, I gave up or reduced important social, occupational, or recreational activities because of stimulant use.	◯	◯
In the year prior to my psychedelic experience, I engaged in recurrent stimulant use in situations in which it was physically hazardous.	◯	◯
In the year prior to my psychedelic experience, I continued using stimulants despite knowledge of having a persistent or recurrent physical or psychological problem that was likely to have been caused or exacerbated by stimulant use.	◯	◯
In the year prior to my psychedelic experience, I experienced stimulant tolerance, as defined by either: A) a need for markedly increased amounts of stimulants to achieve intoxication or desired effect; or B) markedly diminished effect with continued use of the same amount of stimulants.	◯	◯
In the year prior to my psychedelic experience, I experienced stimulant withdrawal, as defined by either: A) the presence of these symptoms upon cessation of stimulant use: fatigue, vivid, unpleasant dreams, insomnia or hypersomnia, increased appetite, psychomotor retardation (slowing of activity), psychomotor agitation (pacing, wringing hands, uncontrollable tongue movement, etc.); or B) Stimulants were taken to relieve or avoid such withdrawal symptoms.	◯	◯

303. Listed below are questions that ask about your feelings about using stimulants <u>in the year prior to your psychedelic-associated stimulant use reduction.</u> The word "using" refers to taking stimulants in order to get high or to "stay regular." Please indicate how much you would have agreed or disagreed with each of the following statements in the year before your psychedelic experience by selecting one response for each question between STRONGLY DISAGREE and STRONGLY AGREE. Please complete every item. <u>We are interested in how you were thinking or feeling generally during the year prior to your psychedelic-associated stimulant use reduction.</u>

	Response
All I would have wanted to do was to use stimulants.	
I would not have needed to use stimulants.	
It would have been difficult to turn down stimulants.	
Using stimulants would have made things seem perfect.	
I would have wanted to use stimulants so bad, I could almost taste it.	
Nothing would have felt better than using stimulants.	
If I had the chance to use stimulants, I don't think I would have.	
I would have craved stimulants.	

The following questions pertain to your stimulant use <u>in the time since your psychedelic experience.</u> Your answers will remain confidential so please be honest. Select the options that best describe your answer to each question.

304. In the period of time since your psychedelic-associated stimulant use reduction, how often did you use stimulants?

- ◯ Never
- ◯ Monthly or less
- ◯ 2-4 times a month
- ◯ 2-3 times a week
- ◯ 4 or more times a week

305. In the period of time since your psychedelic-associated stimulant use reduction, how many times did you take stimulants on a typical day when you were using?

- ◯ 0, not applicable
- ◯ 1 or 2
- ◯ 3 or 4
- ◯ 5 or 6
- ◯ 7 to 9
- ◯ 10 or more

306. In the period of time since your psychedelic-associated stimulant use reduction...

	Frequency
How often in the period of time since your psychedelic experience did you take a noticeable psychoactive dose of a stimulant drug?	
How often in the period of time since your psychedelic experience did you take more stimulants than you thought you would?	
How often in the period of time since your psychedelic experience did you fail to do what was normally expected of you because of your stimulant use?	
How often in the period of time since your psychedelic experience did you need to take stimulants first thing in the morning to get yourself going or to steady your nerves?	
How often in the period of time since your psychedelic experience did you have a feeling of guilt or remorse after using stimulants?	
How often in the period of time since your psychedelic experience were you unable to remember what happened the night before because of your stimulant use?	

307. In the period of time since your psychedelic-associated stimulant use reduction, were you or someone else injured because of your stimulant use?

◯ No

◯ Yes

308. In the period of time since your psychedelic-associated stimulant use reduction, has a relative, friend, doctor, or other health worker been concerned about your stimulant use, or suggest that you should cut down?

◯ No

◯ Yes

309. In the time since your psychedelic-associated stimulant use reduction, please indicate whether each of the following was true or false regarding your stimulant use.

	True	False
In the time since my psychedelic experience, I often consumed stimulants in larger amounts or over a longer period than intended.	◯	◯
In the time since my psychedelic experience, I had a persistent desire or unsuccessful efforts to cut down or control my stimulant use.	◯	◯
In the time since my psychedelic experience, I spent a great deal of time in activities necessary to obtain stimulants, use stimulants, or recover from their effects.	◯	◯
In the time since my psychedelic experience, I experienced craving, or a strong desire or urge to use stimulants.	◯	◯
In the time since my psychedelic experience, my recurrent stimulant use resulted in a failure to fulfill major role obligations at work, school, or home.	◯	◯
In the time since my psychedelic experience, I continued using stimulants despite having persistent or recurrent social or interpersonal problems caused or exacerbated by the effects of stimulants.	◯	◯
In the time since my psychedelic experience, I gave up or reduced important social, occupational, or recreational activities because of stimulant use.	◯	◯
In the time since my psychedelic experience, I engaged in recurrent stimulant use in situations in which it was physically hazardous.	◯	◯
In the time since my psychedelic experience, I continued using stimulants despite knowledge of having a persistent or recurrent physical or psychological problem that was likely to have been caused or exacerbated by stimulant use.	◯	◯
In the time since my psychedelic experience, I experienced stimulant tolerance, as defined by either: A) a need for markedly increased amounts of stimulants to achieve intoxication or desired effect; or B) markedly diminished effect with continued use of the same amount of stimulants.	◯	◯
In the time since my psychedelic experience, I experienced stimulant withdrawal, as defined by either: A) the presence of these symptoms upon cessation of stimulant use: fatigue, vivid, unpleasant dreams, insomnia or hypersomnia, increased appetite, psychomotor retardation (slowing of activity), psychomotor agitation (pacing, wringing hands, uncontrollable tongue movement, etc.); or B) Stimulants were taken to relieve or avoid such withdrawal symptoms.	◯	◯

310. Listed below are questions that ask about your feelings about using stimulants right now. The word "using" refers to taking a stimulant to get high or "stay regular." Please indicate how much you agree or disagree with each of the following statements by selecting one response for each question between STRONGLY DISAGREE and STRONGLY AGREE. Please complete every item. We are interested in how you are thinking or feeling right now.

	Response
All I want to do now is use stimulants.	
I do not need to use stimulants right now.	
It would be difficult to turn down stimulants this minute.	
Using stimulants right now would make things seem perfect.	
I want to use stimulants so bad I can almost taste it.	
Nothing would be better than using stimulants right now.	
If I had the chance to use stimulants now, I don't think I would.	
I crave stimulants right now.	

311. Have you had any stimulants...

	Yes	No
in the past 12 months?	○	○
in the past 6 months?	○	○
in the past month?	○	○
in the past week?	○	○
in the past 24 hours?	○	○

312. How old were you when you first started using stimulants?

313. How many years do you think you had a "stimulant problem" before quitting or reducing your stimulant use?

Other (please specify)

314. Prior to your psychedelic experience, which of the following did you use in your efforts to quit or reduce your stimulant use? (Check all that apply):

- [ ] None
- [ ] Detoxification at a treatment center
- [ ] Professional counseling or psychotherapy
- [ ] Counseling provided by a "Quit-line" telephone number DEL
- [ ] Counseling provided by a website (for example, WebMD)
- [ ] Hypnosis DEL
- [ ] Acupuncture DEL
- [ ] Narcotics Anonymous (NA), or another support group
- [ ] Self-help manuals or books
- [ ] Prescribed medications (please specify in comments section) DEL
- [ ] Spiritual / religious practice (Church attendance, meditation, etc.)

Other/comments (please specify)

315. Prior to your psychedelic-associated stimulant use reduction, how many times in your life had you made a serious attempt to quit taking stimulants (i.e., attempts lasting at least 1 day)?

316. Other than your psychedelic-associated stimulant use reduction, what was the longest period of time that you were able to refrain from taking stimulants?

Other (please specify)

317. Some people report withdrawal symptoms (like the ones listed below) immediately upon stopping or reducing their regular stimulant use. After your psychedelic-associated stimulant use reduction, how serious were each of these problems <u>in comparison to your previous quit attempts?</u>

	Not Applicable	Much Less Severe	Less Severe	Same	More Severe	Much More Severe
Increased appetite	◯	◯	◯	◯	◯	◯
Psychomotor retardation (physical difficulty performing routine tasks)	◯	◯	◯	◯	◯	◯
Psychomotor agitation (pacing, uncontrollable tongue movement, etc.)	◯	◯	◯	◯	◯	◯
Increased Heart rate	◯	◯	◯	◯	◯	◯
Headaches	◯	◯	◯	◯	◯	◯
Drowsiness	◯	◯	◯	◯	◯	◯
Depression or low mood	◯	◯	◯	◯	◯	◯
Confusion	◯	◯	◯	◯	◯	◯
Vivid, unpleasant dreams	◯	◯	◯	◯	◯	◯
Fatigue	◯	◯	◯	◯	◯	◯
Insomnia or Hypersomnia	◯	◯	◯	◯	◯	◯
Difficulty concentrating	◯	◯	◯	◯	◯	◯
Heart pounding, or sweating	◯	◯	◯	◯	◯	◯
Irritability	◯	◯	◯	◯	◯	◯
Restlessness	◯	◯	◯	◯	◯	◯
Anxiety	◯	◯	◯	◯	◯	◯
Fever	◯	◯	◯	◯	◯	◯
Stimulant craving	◯	◯	◯	◯	◯	◯

Comments

318. Would you say your quitting or reducing stimulants was the result of a single or multiple psychedelic sessions?

- ◯ Single Session
- ◯ Multiple Sessions

319. If your stimulant cessation or reduction was the result of multiple psychedelic sessions, please briefly describe the process, including substances ingested, number of sessions, and length of time between sessions.

For this section we would like you to focus on just one psychedelic experience that you deemed to be most important in affecting a decrease in your cannabis use. If you reduced your cannabis use after multiple psychedelic experiences, you will be able to provide more information on the substances, doses, and sequence of events in later sections.

320. How long ago did the psychedelic experience that led to your quitting or reducing cannabis occur?

- Less than 1 week
- 1 - 2 weeks
- 3 - 4 weeks
- 1 - 3 months
- 4 - 6 months
- 7 - 12 months
- 1 - 2 years
- 3 - 5 years
- 6 - 10 years
- 11 - 20 years
- More than 20 years

321. Please characterize your cannabis consumption after this psychedelic experience.

- Stopped smoking completely since the experience (Total abstinence from cannabis).
- Greatly reduced smoking cannabis since the experience.
- Reduced smoking cannabis somewhat since the experience.
- Stopped smoking cannabis completely for a period of time, then returned to smoking the same amount as before.
- Reduced smoking cannabis for a period of time, then returned to smoking the same amount as before.

Other (please specify)

322. How long did your cannabis reduction or abstinence last?

- Less than 1 week
- 1 - 2 weeks
- 3 - 4 weeks
- 1 - 3 months
- 4 - 6 months
- 7 - 12 months
- 1 - 2 years
- 3 - 5 years
- 6 - 10 years
- 11 - 20 years
- More than 20 years

323. Which substance led to the psychedelic experience associated with your quitting or reducing cannabis?

- psilocybin mushrooms
- LSD
- morning glory seeds
- mescaline (pure compound)
- peyote cactus
- San Pedro cactus
- DMT (pure compound)
- Ayahuasca
- MDMA (ecstasy, Molly)

Other (please specify)

324. What was the approximate dose ingested to the best of your knowledge?

- Very low dose
- Low dose
- Moderate dose
- High dose
- Very high dose

325. How old were you when this experience took place?

326. Where did this experience take place? (Check all that apply):

- [ ] At home
- [ ] At a party
- [ ] In a public place (e.g., shopping mall, movie theater, etc.)
- [ ] At a concert or festival
- [ ] Outdoors in nature
- [ ] In a religious or spiritual setting (e.g., ceremony, retreat, etc.)

Other (please specify)

327. Were you alone or with other people during the majority of this psychedelic session?

- ( ) Alone
- ( ) With 1 other person
- ( ) With a few other people (2 - 5)
- ( ) With a small group of people (6 - 20)
- ( ) At a medium-sized gathering (21 - 100)
- ( ) At a large gathering (more than 100 people)

328. What was your intention in this particular instance for taking a psychedelic? (Check all that apply):

- [ ] I took a psychedelic only because other people were, but I did not have a serious intention.
- [ ] Curiosity without any other serious intention.
- [ ] Recreational (e.g., to enjoy the experience, which may or may not involve a social recreational event such as a concert).
- [ ] A serious intention for psychological self-exploration (e.g., resolve a personal issue or to increase self-understanding).
- [ ] A serious intention to explore spirituality or the sacred (e.g., to contemplate God, as you understand that word, or the nature of ultimate reality, and so on).
- [ ] A premeditated intention to quit or reduce your cannabis use.
- [ ] Other (please specify)

329. After your psychedelic experience, did you use any other methods to help maintain cannabis abstinence or reduction? (Check all that apply):

- [ ] None
- [ ] Detoxification at a treatment center
- [ ] Professional counseling or psychotherapy
- [ ] Counseling provided by a "Quit-line" telephone number DEL
- [ ] Counseling provided by a website (for example, WebMD)
- [ ] Prescribed medication (please specify in Comments section)
- [ ] Hypnosis DEL
- [ ] Acupuncture DEL
- [ ] Support group DEL (Marijuana Anonymous, etc.)
- [ ] Self-help manuals or books DEL
- [ ] Spiritual / religious practice (Church attendance, meditation, etc.)
- [ ] Other / comments (please specify)

330. Please provide a brief description of the psychedelic experience that led to your quitting or reducing cannabis.

Looking back on the entirety of the psychedelic session that occurred prior to your cannabis reduction or cessation, please rate the degree to which at any time during that session you experienced the following phenomena. Answer each question according to your feelings, thoughts, and experiences at the time of the psychedelic session.

331. Loss of your usual sense of time.

- 0 – none; not at all
- 1 – so slight cannot decide
- 2 – slight
- 3 – moderate
- 4 – strong (equivalent in degree to any other strong experience)
- 5 – extreme (more than any other time in my life and stronger than 4)

332. Experience of amazement.

- 0 – none; not at all
- 1 – so slight cannot decide
- 2 – slight
- 3 – moderate
- 4 – strong (equivalent in degree to any other strong experience)
- 5 – extreme (more than any other time in my life and stronger than 4)

333. Sense that the experience cannot be described adequately in words.

- 0 – none; not at all
- 1 – so slight cannot decide
- 2 – slight
- 3 – moderate
- 4 – strong (equivalent in degree to any other strong experience)
- 5 – extreme (more than any other time in my life and stronger than 4)

334. Gain of insightful knowledge experienced at an intuitive level.

- 0 – none; not at all
- 1 – so slight cannot decide
- 2 – slight
- 3 – moderate
- 4 – strong (equivalent in degree to any other strong experience)
- 5 – extreme (more than any other time in my life and stronger than 4)

335. Feeling that you experienced eternity or infinity.

- 0 – none; not at all
- 1 – so slight cannot decide
- 2 – slight
- 3 – moderate
- 4 – strong (equivalent in degree to any other strong experience)
- 5 – extreme (more than any other time in my life and stronger than 4)

336. Experience of oneness or unity with objects and/or persons perceived in your surroundings.

- 0 – none; not at all
- 1 – so slight cannot decide
- 2 – slight
- 3 – moderate
- 4 – strong (equivalent in degree to any other strong experience)
- 5 – extreme (more than any other time in my life and stronger than 4)

337. Loss of your usual sense of space.

- 0 – none; not at all
- 1 – so slight cannot decide
- 2 – slight
- 3 – moderate
- 4 – strong (equivalent in degree to any other strong experience)
- 5 – extreme (more than any other time in my life and stronger than 4)

338. Feelings of tenderness and gentleness.

- 0 – none; not at all
- 1 – so slight cannot decide
- 2 – slight
- 3 – moderate
- 4 – strong (equivalent in degree to any other strong experience)
- 5 – extreme (more than any other time in my life and stronger than 4)

339. Certainty of encounter with ultimate reality (in the sense of being able to "know" and "see" what is really real at some point during your experience).

- 0 – none; not at all
- 1 – so slight cannot decide
- 2 – slight
- 3 – moderate
- 4 – strong (equivalent in degree to any other strong experience)
- 5 – extreme (more than any other time in my life and stronger than 4)

340. Feeling that you could not do justice to your experience by describing it in words.

- 0 – none; not at all
- 1 – so slight cannot decide
- 2 – slight
- 3 – moderate
- 4 – strong (equivalent in degree to any other strong experience)
- 5 – extreme (more than any other time in my life and stronger than 4)

341. Loss of usual awareness of where you were.

- 0 – none; not at all
- 1 – so slight cannot decide
- 2 – slight
- 3 – moderate
- 4 – strong (equivalent in degree to any other strong experience)
- 5 – extreme (more than any other time in my life and stronger than 4)

342. Feelings of peace and tranquility.

- 0 – none; not at all
- 1 – so slight cannot decide
- 2 – slight
- 3 – moderate
- 4 – strong (equivalent in degree to any other strong experience)
- 5 – extreme (more than any other time in my life and stronger than 4)

343. Sense of being "outside of" time, beyond past and future.

- 0 – none; not at all
- 1 – so slight cannot decide
- 2 – slight
- 3 – moderate
- 4 – strong (equivalent in degree to any other strong experience)
- 5 – extreme (more than any other time in my life and stronger than 4)

344. Freedom from the limitations of your personal self and feeling a unity or bond with what was felt to be greater than your personal self.

- 0 – none; not at all
- 1 – so slight cannot decide
- 2 – slight
- 3 – moderate
- 4 – strong (equivalent in degree to any other strong experience)
- 5 – extreme (more than any other time in my life and stronger than 4)

345. Sense of being at a spiritual height.

- 0 – none; not at all
- 1 – so slight cannot decide
- 2 – slight
- 3 – moderate
- 4 – strong (equivalent in degree to any other strong experience)
- 5 – extreme (more than any other time in my life and stronger than 4)

346. Experience of pure being and pure awareness (beyond the world of sense impressions).

- 0 – none; not at all
- 1 – so slight cannot decide
- 2 – slight
- 3 – moderate
- 4 – strong (equivalent in degree to any other strong experience)
- 5 – extreme (more than any other time in my life and stronger than 4)

347. Experience of ecstasy.

- 0 – none; not at all
- 1 – so slight cannot decide
- 2 – slight
- 3 – moderate
- 4 – strong (equivalent in degree to any other strong experience)
- 5 – extreme (more than any other time in my life and stronger than 4)

348. Experience of the insight that “all is One”.

- 0 – none; not at all
- 1 – so slight cannot decide
- 2 – slight
- 3 – moderate
- 4 – strong (equivalent in degree to any other strong experience)
- 5 – extreme (more than any other time in my life and stronger than 4)

349. Being in a realm with no space boundaries.

- 0 – none; not at all
- 1 – so slight cannot decide
- 2 – slight
- 3 – moderate
- 4 – strong (equivalent in degree to any other strong experience)
- 5 – extreme (more than any other time in my life and stronger than 4)

350. Experience of oneness in relation to an “inner world” within.

- 0 – none; not at all
- 1 – so slight cannot decide
- 2 – slight
- 3 – moderate
- 4 – strong (equivalent in degree to any other strong experience)
- 5 – extreme (more than any other time in my life and stronger than 4)

351. Sense of reverence.

- 0 – none; not at all
- 1 – so slight cannot decide
- 2 – slight
- 3 – moderate
- 4 – strong (equivalent in degree to any other strong experience)
- 5 – extreme (more than any other time in my life and stronger than 4)

352. Experience of timelessness.

- 0 – none; not at all
- 1 – so slight cannot decide
- 2 – slight
- 3 – moderate
- 4 – strong (equivalent in degree to any other strong experience)
- 5 – extreme (more than any other time in my life and stronger than 4)

353. You are convinced now, as you look back on your experience, that in it you encountered ultimate reality (i.e., that you "knew" and "saw" what was really real).

- 0 – none; not at all
- 1 – so slight cannot decide
- 2 – slight
- 3 – moderate
- 4 – strong (equivalent in degree to any other strong experience)
- 5 – extreme (more than any other time in my life and stronger than 4)

354. Feeling that you experienced something profoundly sacred and holy.

- 0 – none; not at all
- 1 – so slight cannot decide
- 2 – slight
- 3 – moderate
- 4 – strong (equivalent in degree to any other strong experience)
- 5 – extreme (more than any other time in my life and stronger than 4)

355. Awareness of the life or living presence in all things.

- ◯ 0 – none; not at all
- ◯ 1 – so slight cannot decide
- ◯ 2 – slight
- ◯ 3 – moderate
- ◯ 4 – strong (equivalent in degree to any other strong experience)
- ◯ 5 – extreme (more than any other time in my life and stronger than 4)

356. Experience of the fusion of your personal self into a larger whole.

- ◯ 0 – none; not at all
- ◯ 1 – so slight cannot decide
- ◯ 2 – slight
- ◯ 3 – moderate
- ◯ 4 – strong (equivalent in degree to any other strong experience)
- ◯ 5 – extreme (more than any other time in my life and stronger than 4)

357. Sense of awe or awesomeness.

- ◯ 0 – none; not at all
- ◯ 1 – so slight cannot decide
- ◯ 2 – slight
- ◯ 3 – moderate
- ◯ 4 – strong (equivalent in degree to any other strong experience)
- ◯ 5 – extreme (more than any other time in my life and stronger than 4)

358. Experience of unity with ultimate reality.

- ◯ 0 – none; not at all
- ◯ 1 – so slight cannot decide
- ◯ 2 – slight
- ◯ 3 – moderate
- ◯ 4 – strong (equivalent in degree to any other strong experience)
- ◯ 5 – extreme (more than any other time in my life and stronger than 4)

359. Feeling that it would be difficult to communicate your own experience to others who have not had similar experiences.

- 0 – none; not at all
- 1 – so slight cannot decide
- 2 – slight
- 3 – moderate
- 4 – strong (equivalent in degree to any other strong experience)
- 5 – extreme (more than any other time in my life and stronger than 4)

360. Feelings of joy.

- 0 – none; not at all
- 1 – so slight cannot decide
- 2 – slight
- 3 – moderate
- 4 – strong (equivalent in degree to any other strong experience)
- 5 – extreme (more than any other time in my life and stronger than 4)

The purpose of the following questions is to obtain your retrospective assessment of the psychedelic experience that led to your cannabis use reduction, <u>and your contemplation of that experience.</u> For all questions on this page, please choose the highest rating that applies.

361. How personally meaningful were the psychedelic experience that led to your cannabis use reduction, and your contemplation of that experience?

- No more than routine, everyday experiences
- Similar to meaningful experiences that occur on average once or more a week
- Similar to meaningful experiences that occur on average once a month
- Similar to meaningful experiences that occur on average once a year
- Similar to meaningful experiences that occur on average once every 5 years
- Among the 10 most meaningful experiences of my life
- Among the 5 most meaningful experiences of my life
- The single most meaningful experience of my life

362. Indicate the degree to which the psychedelic experience that led to your cannabis use reduction, and your contemplation of that experience, were spiritually significant to you.

- Not at all
- Slightly
- Moderately
- Very much
- Among the 5 most spiritually significant experiences of my life
- The single most spiritually significant experience of my life

363. How psychologically challenging was the most psychologically challenging portion of the psychedelic experience that led to your cannabis use reduction?

- No more than routine, everyday experiences
- Similar to difficult or challenging experiences that occur on average once or more a week
- Similar to difficult or challenging experiences that occur on average once a month
- Similar to difficult or challenging experiences that occur on average once a year
- Similar to difficult or challenging experiences that occur on average once every 5 years
- Among the 10 most difficult or challenging experiences of my life
- Among the 5 most difficult or challenging experiences of my life
- The single most difficult or challenging experience of my life

364. How psychologically insightful to you were the psychedelic experience that led to your cannabis use reduction, and your contemplation of that experience?

- No more than routine, everyday psychologically insightful experiences
- Similar to psychologically insightful experiences that occur on average once or more a week
- Similar to psychologically insightful experiences that occur on average once a month
- Similar to psychologically insightful experiences that occur on average once a year
- Similar to psychologically insightful experiences that occur on average once every 5 years
- Among the 10 most psychologically insightful experiences of my life
- Among the 5 most psychologically insightful experiences of my life
- The single most psychologically insightful experience of my life

365. Do you believe that the psychedelic experience that led to your cannabis use reduction, and your contemplation of that experience, have led to change in your current sense of personal well-being or life satisfaction?

- Strong positive change that I consider desirable
- Moderate positive change that I consider desirable
- Slight positive change that I consider desirable
- No change
- Slight negative change that I consider undesirable
- Moderate negative change that I consider undesirable
- Strong negative change that I consider undesirable

366. Please rank items in order of their importance to your psychedelic-associated cannabis abstinence or reduction.

	Strengthening your belief in your own ability to quit.	Not applicable
	Reducing stress involved with quitting.	Not applicable
	Reframing quitting as a spiritual task.	Not applicable
	Changing life priorities or values, such that using cannabis was no longer more important than quitting.	Not applicable
	Changing your orientation toward the future, so that long-term benefits outweighed immediate desires.	Not applicable
	Increasing space between the experience of craving and taking action.	Not applicable

367. Please describe any additional reasons (not listed above) that your psychedelic experience may have contributed to your quitting or reducing your cannabis use.

368. Did you experience any other behavioral changes after this psychedelic session? Check all that apply, and please provide any relevant details in the Comments box below.

- [ ] None
- [ ] Reduced or stopped using other drugs
- [ ] Started using other drugs more often / heavily
- [ ] Improved diet / nutrition
- [ ] Worsened diet / nutrition
- [ ] Increased physical activity / exercise
- [ ] Decreased physical activity / exercise
- [ ] Improved relationships with others
- [ ] Worsened relationships with others
- [ ] Improvements in career / work life
- [ ] Worsening of career / work life

Other / comments

369. Did you experience any persisting negative effects from this psychedelic experience? By persisting, we mean negative effects that lasted beyond the acute period of drug effects.

- Yes
- No
- Not sure

370. Please describe any negative or potentially negative persisting effects you may have experienced as a result of this psychedelic session.

371. Overall, how would you rate the severity of these negative effects?

- Not at all severe
- Slightly severe
- Moderately severe
- Very severe
- Extremely severe

The following questions pertain to your cannabis use <u>in the year prior to your psychedelic experience.</u> Your answers will remain confidential so please be honest. Select the options that best describe your answer to each question.

372. In the year prior to your psychedelic-associated cannabis use reduction, how often did you smoke/consume cannabis?

- ◯ Never
- ◯ Monthly or less
- ◯ 2-4 times a month
- ◯ 2-3 times a week
- ◯ 4 or more times a week

373. In the year prior to your psychedelic-associated cannabis use reduction, how many times did you smoke/consume cannabis on a typical day when you were using?

- ◯ 1 or 2
- ◯ 3 or 4
- ◯ 5 or 6
- ◯ 7 to 9
- ◯ 10 or more

374. During the year prior to your psychedelic-associated cannabis use reduction...

	Frequency
How often during the year prior to your psychedelic-associated cannabis use or reduction did you smoke/consume a noticeable psychoactive dose of cannabis?	
How often during the year prior to your psychedelic-associated cannabis use reduction did you find that once you started smoking you couldn't stop?	
How often during the year prior to your psychedelic-associated cannabis use reduction did you fail to do what was normally expected of you because of your cannabis use?	
How often during the year prior to your psychedelic-associated cannabis use reduction did you need to smoke/consume cannabis first thing in the morning to get yourself going or to steady your nerves?	
How often during the year prior to your psychedelic-associated cannabis use reduction did you have a feeling of guilt or remorse after smoking/consuming cannabis?	
How often during the year prior to your psychedelic-associated cannabis use reduction were you unable to remember what happened the night before because of your cannabis use?	

375. During the year prior to your psychedelic-associated cannabis use reduction, were you or someone else injured because of your cannabis use?

- ◯ No
- ◯ Yes

376. During the year prior to your psychedelic-associated cannabis use reduction, was a relative, friend, doctor, or other health worker concerned about your cannabis use, or suggest that you should cut down?

- ◯ No
- ◯ Yes

377. In the year prior to your psychedelic-associated cannabis use reduction, please indicate whether each of the following was true or false regarding your cannabis use.

	True	False
In the year prior to my psychedelic experience, I often smoked/consumed cannabis in larger amounts or over a longer period than intended.	◯	◯
In the year prior to my psychedelic experience, I had a persistent desire or unsuccessful efforts to cut down or control my cannabis use.	◯	◯
In the year prior to my psychedelic experience, I spent a great deal of time in activities necessary to obtain cannabis, use cannabis, or recover from its effects.	◯	◯
In the year prior to my psychedelic experience, I experienced craving, or a strong desire or urge to use cannabis.	◯	◯
In the year prior to my psychedelic experience, my recurrent cannabis use resulted in a failure to fulfill major role obligations at work, school, or home.	◯	◯
In the year prior to my psychedelic experience, I continued using cannabis despite having persistent or recurrent social or interpersonal problems caused or exacerbated by the effects of cannabis.	◯	◯
In the year prior to my psychedelic experience, I gave up or reduced important social, occupational, or recreational activities because of cannabis use.	◯	◯
In the year prior to my psychedelic experience, I engaged in recurrent cannabis use in situations in which it was physically hazardous.	◯	◯
In the year prior to my psychedelic experience, I continued using cannabis despite knowledge of having a persistent or recurrent physical or psychological problem that was likely to have been caused or exacerbated by cannabis.	◯	◯
In the year prior to my psychedelic experience, I experienced cannabis tolerance, as defined by either: A) a need for markedly increased amounts of cannabis to achieve intoxication or desired effect; or B) markedly diminished effect with continued use of the same amount of cannabis.	◯	◯
In the year prior to my psychedelic experience, I experienced cannabis withdrawal, as defined by either: A) the presence of these symptoms upon cessation of cannabis use: irritability, anger or aggression, nervousness or anxiety, sleep difficulty (e.g., insomnia, disturbing dreams), decreased appetite or weight loss, restlessness, depressed mood, abdominal pain, shakiness/tremors, sweating, fever, chills, or headache; or B) Cannabis was taken to relieve or avoid such withdrawal symptoms.	◯	◯

378. Listed below are questions that ask about your feelings about using cannabis in the year prior to your psychedelic-associated cannabis use reduction. The word "using" refers to smoking cannabis or eating anything containing cannabis in order to get high or to "stay regular." Please indicate how much you would have agreed or disagreed with each of the following statements in the year before your psychedelic experience by selecting one response for each question between STRONGLY DISAGREE and STRONGLY AGREE. Please complete every item. We are interested in how you were thinking or feeling generally during the year prior to your psychedelic-associated cannabis use reduction.

	Response
All I would have wanted to do was to use cannabis.	
I would not have needed to use cannabis.	
It would have been difficult to turn down cannabis.	
Using cannabis would have made things seem perfect.	
I would have wanted to use cannabis so bad, I could almost taste it.	
Nothing would have felt better than using cannabis.	
If I had the chance to use cannabis, I don't think I would have.	
I would have craved cannabis.	

The following questions pertain to your cannabis use in the time since your psychedelic experience. Your answers will remain confidential so please be honest. Select the options that best describe your answer to each question.

379. In the period of time since your psychedelic-associated cannabis use reduction, how often did you smoke/consume cannabis?

- ◯ Never
- ◯ Monthly or less
- ◯ 2-4 times a month
- ◯ 2-3 times a week
- ◯ 4 or more times a week

380. In the period of time since your psychedelic-associated cannabis use reduction, how many times did you smoke/consume cannabis on a typical day when you were using?

- ◯ 0, not applicable
- ◯ 1 or 2
- ◯ 3 or 4
- ◯ 5 or 6
- ◯ 7 to 9
- ◯ 10 or more

381. In the period of time since your psychedelic-associated cannabis use reduction...

	Frequency
How often in the period of time since your psychedelic experience did you smoke/consume a noticeable psychoactive dose of cannabis?	
How often in the period of time since your psychedelic experience did you find that once you started smoking you couldn't stop?	
How often in the period of time since your psychedelic experience did you fail to do what was normally expected of you because of your cannabis use?	
How often in the period of time since your psychedelic experience did you need to smoke/consume cannabis first thing in the morning to get yourself going or to steady your nerves?	
How often in the period of time since your psychedelic experience did you have a feeling of guilt or remorse after smoking/consuming cannabis?	
How often in the period of time since your psychedelic experience were you unable to remember what happened the night before because of your cannabis use?	

382. In the period of time since your psychedelic-associated cannabis use reduction, were you or someone else injured as a result of your cannabis use?

- ◯ No
- ◯ Yes

383. In the period of time since your psychedelic-associated cannabis use reduction, has a relative, friend, doctor, or other health worker been concerned about your cannabis use, or suggest that you should cut down?

- ◯ No
- ◯ Yes

384. In the time since your psychedelic-associated cannabis use reduction, please indicate whether each of the following was true or false regarding your cannabis use.

	True	False
In the time since my psychedelic experience, I often smoked/consumed cannabis in larger amounts or over a longer period than intended.	◯	◯
In the time since my psychedelic experience, I had a persistent desire or unsuccessful efforts to cut down or control my cannabis use.	◯	◯
In the time since my psychedelic experience, I spent a great deal of time in activities necessary to obtain cannabis, use cannabis, or recover from its effects.	◯	◯
In the time since my psychedelic experience, I experienced craving, or a strong desire or urge to use cannabis.	◯	◯
In the time since my psychedelic experience, my recurrent cannabis use resulted in a failure to fulfill major role obligations at work, school, or home.	◯	◯
In the time since my psychedelic experience, I continued using cannabis despite having persistent or recurrent social or interpersonal problems caused or exacerbated by the effects of cannabis.	◯	◯
In the time since my psychedelic experience, I gave up or reduced important social, occupational, or recreational activities because of my cannabis use.	◯	◯
In the time since my psychedelic experience, I engaged in recurrent cannabis use in situations in which it was physically hazardous.	◯	◯
In the time since my psychedelic experience, I continued using cannabis despite knowledge of having a persistent or recurrent physical or psychological problem that was likely to have been caused or exacerbated by cannabis.	◯	◯
In the time since my psychedelic experience, I experienced cannabis tolerance, as defined by either: A) a need for markedly increased amounts of cannabis to achieve intoxication or desired effect; or B) markedly diminished effect with continued use of the same amount of cannabis.	◯	◯
In the time since my psychedelic experience, I experienced cannabis withdrawal, as defined by either: A) the presence of these symptoms upon cessation of cannabis use: irritability, anger, or aggression, nervousness or anxiety, sleep difficulty (e.g., insomnia, disturbing dreams), decreased appetite or weight loss, restlessness, depressed mood, abdominal pain, shakiness/tremors, sweating, fever, chills, or headache; or B) Cannabis was taken to relieve or avoid such withdrawal symptoms.	◯	◯

385. Listed below are questions that ask about your feelings about using cannabis right now. The word "using" refers to smoking cannabis, or eating anything containing cannabis in order to get high or "stay regular." Please indicate how much you agree or disagree with each of the following statements by selecting one response for each question between STRONGLY DISAGREE and STRONGLY AGREE. Please complete every item. We are interested in how you are thinking or feeling right now.

	Response
All I want to do now is use cannabis.	
I do not need to use cannabis right now.	
It would be difficult to turn down cannabis this minute.	
Using cannabis right now would make things seem perfect.	
I want to use cannabis so bad I can almost taste it.	
Nothing would be better than using cannabis right now.	
If I had the chance to use cannabis now, I don't think I would.	
I crave cannabis right now.	

386. Have you used cannabis...

	Yes	No
in the past 12 months?	○	○
in the past 6 months?	○	○
in the past month?	○	○
in the past week?	○	○
in the past 24 hours?	○	○

387. How old were you when you first started using cannabis?

388. How many years do you think you had a "cannabis problem" before quitting or reducing your cannabis use?

Other (please specify)

389. Prior to your psychedelic experience, which of the following did you use in your efforts to quit or reduce your cannabis consumption? (Check all that apply):

- [ ] None
- [ ] Detoxification at a treatment center
- [ ] Professional counseling or psychotherapy
- [ ] Counseling provided by a "Quit-line" telephone number DEL
- [ ] Counseling provided by a website (for example, WebMD)
- [ ] Hypnosis DEL
- [ ] Acupuncture DEL
- [ ] Marijuana Anonymous (MA), or another support group
- [ ] Self-help manuals or books
- [ ] Prescribed medication (please specify in comments section below) DEL
- [ ] Spiritual / religious practice (Church attendance, meditation, etc.)

Other/comments (please specify)

390. Prior to your psychedelic-associated cannabis use reduction, how many times in your life had you made a serious attempt to quit using cannabis (i.e., attempts lasting at least 1 day)?

391. Other than your psychedelic-associated cannabis use reduction, what was the longest period of time that you were able to refrain from smoking/consuming cannabis?

Other (please specify)

392. Some people report withdrawal symptoms (like the ones listed below) immediately upon stopping or reducing their regular cannabis use. After your psychedelic-associated cannabis use reduction, how serious were each of these problems <u>in comparison to your previous quit attempts?</u>

	Not Applicable	Much Less Severe	Less Severe	Same	More Severe	Much More Severe
Decreased appetite or weight loss	○	○	○	○	○	○
Shakiness/tremors	○	○	○	○	○	○
Nausea and/or vomiting	○	○	○	○	○	○
Increased Heart rate	○	○	○	○	○	○
Headaches	○	○	○	○	○	○
Drowsiness	○	○	○	○	○	○
Depression or low mood	○	○	○	○	○	○
Confusion	○	○	○	○	○	○
Fatigue	○	○	○	○	○	○
Sleep difficulty (e.g., insomnia, disturbing dreams)	○	○	○	○	○	○
Difficulty concentrating	○	○	○	○	○	○
Heart pounding, or sweating	○	○	○	○	○	○
Irritability, anger, or aggression	○	○	○	○	○	○
Restlessness	○	○	○	○	○	○
Hallucinations	○	○	○	○	○	○
Seizures	○	○	○	○	○	○
Chills	○	○	○	○	○	○
Fever	○	○	○	○	○	○
Nervousness or anxiety	○	○	○	○	○	○
Cannabis craving	○	○	○	○	○	○

Comments

393. Would you say your quitting or reducing your cannabis use was the result of a single or multiple psychedelic sessions?

- ◯ Single Session
- ◯ Multiple Sessions

394. If your cannabis cessation or reduction was the result of multiple psychedelic sessions, please briefly describe the process, including substances ingested, number of sessions, and length of time between sessions.

Great! You've completed the majority of the main survey. Just a few more pages to go. These next two sections will assess some personality and dispositional tendencies that may be associated with drug use and altered states of consciousness.

Select whether each item is true or false for you <u>regarding your general experiences in day to day life.</u>

395. Sometimes I feel and experience things as I did when I was a child.

- ◯ True
- ◯ False

396. I can be greatly moved by eloquent or poetic language.

- ◯ True
- ◯ False

397. While watching a movie, a TV show, or a play, I may become so involved that I may forget about myself and my surroundings and experience the story as if it were real and as if I were taking part in it.

- True
- False

398. If I stare at a picture and then look away from it, I can sometimes "see" an image of the picture almost as if I were still looking at it.

- True
- False

399. Sometimes I feel as if my mind could envelop the whole world.

- True
- False

400. I like to watch cloud shapes change in the sky.

- True
- False

401. If I wish I can imagine (or daydream) some things so vividly that they hold my attention as a good movie or story does.

- True
- False

402. I think I really know what some people mean when they talk about mystical experiences.

- True
- False

403. I sometimes "step outside" my usual self and experience an entirely different state of being.

- True
- False

404. Textures- such as wool, sand, wood - sometimes remind me of colors or music.

- True
- False

405. Sometimes I experience things as if they were doubly real.

- True
- False

406. When I listen to music I can get so caught up in it that I don't notice anything else.

- ◯ True
- ◯ False

407. If I wish I can imagine that my body is so heavy that I could not move it if I wanted to.

- ◯ True
- ◯ False

408. I can often somehow sense the presence of another person before I actually see her/him.

- ◯ True
- ◯ False

409. The crackle and flames of a wood fire stimulate my imagination.

- ◯ True
- ◯ False

410. It is sometimes possible for me to be completely immersed in nature or in art and feel as if my whole state of consciousness has somehow been temporarily altered.

- ◯ True
- ◯ False

411. Different colors have distinctive and special meanings for me.

- ◯ True
- ◯ False

412. I am able to wander off into my thoughts while doing a routine task and actually forget that I am doing the task, and then find a few minutes later that I have completed it.

- ◯ True
- ◯ False

413. I can sometimes recollect certain past experiences in my life with such clarity and vividness that it is like living them again or almost so.

- ◯ True
- ◯ False

414. Things that might seem meaningless to others often make sense to me.

- ◯ True
- ◯ False

415. While acting in a play I think I could really feel the emotions of the character and "become" her/him for the time being, forgetting both myself and the audience.

- ◯ True
- ◯ False

416. My thoughts often don't occur as words but as visual images.

- ◯ True
- ◯ False

417. I often take delight in small things (like the five-pointed star shape that appears when you cut an apple across the core or the colors in soap bubbles).

- ◯ True
- ◯ False

418. When listening to organ music or other powerful music I sometimes feel as if I am being lifted into the air.

- ◯ True
- ◯ False

419. Sometimes I can change noise into music by the way that I listen to it.

- ◯ True
- ◯ False

420. Some of my most vivid memories are called up by scents and smells.

- ◯ True
- ◯ False

421. Some music reminds me of pictures or changing color patterns.

- ◯ True
- ◯ False

422. I often know what someone is going to say before he or she says it.

- ◯ True
- ◯ False

423. I often have "physical memories;" for example, after I have been swimming I may still feel as if I am still in the water.

- ◯ True
- ◯ False

424. The sound of a voice can be so fascinating to me that I can just go on listening to it.

- True
- False

425. At times I somehow feel the presence of someone who is not there.

- True
- False

426. Sometimes thoughts and images come to me without the slightest effort on my part.

- True
- False

427. I find that different odors have different colors.

- True
- False

428. I can be deeply moved by a sunset.

- True
- False

Using the scale provided as a guide, indicate how much you agree or disagree with each of the following statements. Give only one answer for each statement.

429. I am often confused about what emotion I am feeling.

430. It is difficult for me to find the right words for my feelings.

431. I have physical sensations that even doctors don't understand.

432. I am able to describe my feelings easily.

433. I prefer to analyze problems rather than just describe them.

434. When I am upset, I don't know if I am sad, frightened, or angry.

435. I am often puzzled by sensations in my body.

436. I prefer to just let things happen rather than to understand why they turned out that way.

437. I have feelings that I can't quite identify.

438. Being in touch with emotions is essential.

439. I find it hard to describe how I feel about people.

440. People tell me to describe my feelings more.

441. I don't know what's going on inside me.

442. I often don't know why I am angry.

443. I prefer talking to people about their daily activities rather then their feelings.

444. I prefer to watch "light" entertainment shows rather than psychological dramas.

445. It is difficult for me to reveal my innermost feelings, even to close friends.

446. I can feel close to someone, even in moments of silence.

447. I find examination of my feelings useful in solving personal problems.

448. Looking for hidden meanings in movies or plays distracts from enjoyment.

449. Did you have any computer problems that prevented you from answering some of the questions on this survey? If so, please explain in Comment box below.

- ◯ Yes
- ◯ No

Comments (please specify)

450. Did you have trouble understanding any of the questions on this survey? If so, please explain in the Comment box below.

- ◯ Yes
- ◯ No

Comments (please specify)

451. Do you have anything to add about psychedelics or your experiences using them that you think would be valuable to know?

452. Thank you very much for your time and participation! You have completed the initial part of the survey, providing us with critical information about the experience of quitting or reducing alcohol or other drug use after a psychedelic experience. At this point you are invited to continue to answer some additional questions that could further help us to characterize this phenomenon. These questions will ask you to make some decisions regarding money and health. If you are interested in continuing with this optional part of the survey, which should take about 10 more minutes to complete, please choose Yes. If not, please choose No to submit your previous responses, and officially exit and complete the survey. If you begin the next part of the survey and then decide to quit before completing it, your previous answers will still be used for our research. Thank you!

- ◯ Yes, I want to continue on to the optional questions.
- ◯ No, I would like to stop now and submit my previous responses.

For each of the next 9 choices, please choose which reward you would prefer: the smaller reward today, or the larger reward in the specified number of days. Please take the choices seriously. Although the choices are pretend and you won't receive the money, please make each decision as if it were really for money.

453. Would you prefer $19 today, or $25 in 53 days?

- ◯ $19 today
- ◯ $25 in 53 days

454. Would you prefer $14 today, or $25 in 19 days?

- ◯ $14 today
- ◯ $25 in 19 days

455. Would you prefer $15 today, or $35 in 13 days?

- ◯ $15 today
- ◯ $35 in 13 days

456. Would you prefer $11 today, or $30 in 7 days?

- ◯ $11 today
- ◯ $30 in 7 days

457. Would you prefer $34 today, or $35 in 186 days?

- ◯ $34 today
- ◯ $35 in 186 days

458. Would you prefer $24 today, or $35 in 29 days?

- ◯ $24 today
- ◯ $35 in 29 days

459. Would you prefer $28 today, or $30 in 179 days?

- ◯ $28 today
- ◯ $30 in 179 days

460. Would you prefer $25 today, or $30 in 80 days?

- ◯ $25 today
- ◯ $30 in 80 days

461. Would you prefer $22 today, or $25 in 136 days?

- ◯ $22 today
- ◯ $25 in 136 days

For each of the next 9 choices, please choose which reward you would prefer: the smaller reward today, or the larger reward in the specified number of days. Please take the choices seriously. Although the choices are pretend and you won't receive the consequences, please make each decision as if it were for real consequences.

For these questions you will be asked about your preference for durations of improved health. By improved health, we mean that you are less likely to catch a cold or other infection, you get better quality sleep, you feel more well rested and energetic, you have less of a cough, and you feel more fit overall.

462. Would you prefer 19 days of improved health starting today, or 25 days of improved health starting in 53 days?

- ◯ 19 days of improved health starting today
- ◯ 25 days of improved health starting in 53 days

463. Would you prefer 14 days of improved health starting today, or 25 days of improved health starting in 19 days?

- ◯ 14 days of improved health starting today
- ◯ 25 days of improved health starting in 19 days

464. Would you prefer 15 days of improved health starting today, or 35 days of improved health starting in 13 days?

- ◯ 15 days of improved health starting today
- ◯ 35 days of improved health starting in 13 days

465. Would you prefer 11 days of improved health starting today, or 30 days of improved health starting in 7 days?

- ◯ 11 days of improved health starting today
- ◯ 30 days of improved health starting in 7 days

466. Would you prefer 34 days of improved health starting today, or 35 days of improved health starting in 186 days?

- ◯ 34 days of improved health starting today
- ◯ 35 days of improved health starting in 186 days

467. Would you prefer 24 days of improved health starting today, or 35 days of improved health starting in 29 days?

- ◯ 24 days of improved health starting today
- ◯ 35 days of improved health starting in 29 days

468. Would you prefer 28 days of improved health starting today, or 30 days of improved health starting in 179 days?

- ◯ 28 days of improved health starting today
- ◯ 30 days of improved health starting in 179 days

469. Would you prefer 25 days of improved health starting today, or 30 days of improved health starting in 80 days?

- ◯ 25 days of improved health starting today
- ◯ 30 days of improved health starting in 80 days

470. Would you prefer 22 days of improved health starting today, or 25 days of improved health starting in 136 days?

- ◯ 22 days of improved health starting today
- ◯ 25 days of improved health starting in 136 days

Thank you very much for your time and participation. You have completed the survey, providing us with critical information about the experience of quitting or reducing alcohol or other drug use after a psychedelic experience. Please feel free to share this survey information with any other interested parties you may know. Have a nice day!
